# Spatially and Seasonally Resolved Predictions Reveal Widespread Ecotoxicological Risk from Pharmaceutical Mixtures in German (Saxon) Rivers

**DOI:** 10.1021/acs.est.5c01639

**Published:** 2025-08-15

**Authors:** Shixue Wu, Björn Helm, Geovanni Teran-Velasquez, Peter Krebs, Rohini Kumar

**Affiliations:** † Department of Computational Hydrosystems, Helmholtz Centre for Environmental Research—UFZ, 04318 Leipzig, Germany; ‡ Institute of Urban and Industrial Water Management, Technische Universität Dresden, 01069 Dresden, Germany

**Keywords:** pharmaceutical pollution, transport and fate modeling, node-link network, ecotoxicological risk assessment, mixture toxicity, German rivers

## Abstract

Pharmaceutical pollution is escalating due to the increasing prevalence of diseases driven by an aging population and socioeconomic and hydroclimatic changes, challenging the EU’s goal of achieving a toxic-free environment. To comprehensively assess pharmaceutical pollution in rivers, we developed a spatially resolved model to predict pharmaceutical concentrations and associated ecological risks across 1 km river stretches in Saxony, Germany. We focused on five pharmaceuticals: two antiepileptics (carbamazepine, gabapentin), two antibiotics (ciprofloxacin, sulfamethoxazole), and one antidiabetic (metformin); and their toxicity to three aquatic species: algae, daphnia, and fish. Model evaluation demonstrated a good level of accuracy, with 95–100% of simulations aligning within 1 order of magnitude of observed values across spatial and temporal scales (2008–2014). Pharmaceutical-wise, low environmental concentrations led to a reduced performance of ciprofloxacin, whereas frequent observations of carbamazepine demonstrated its improved model skill. Further, ecological risk assessments for single toxicity indicated significant risks in over half of the Saxon rivers, with exposure frequencies reaching up to 80% for the analyzed pharmaceuticals. For mixture toxicity, the risk frequency increased to 99%, revealing widespread ecotoxicological risks. Our framework identifies transport trajectories and risk hotspots of pharmaceutical pollution, enabling spatiotemporal predictions under global change conditions to support proactive measures for a healthier planet.

## Graphical abstract

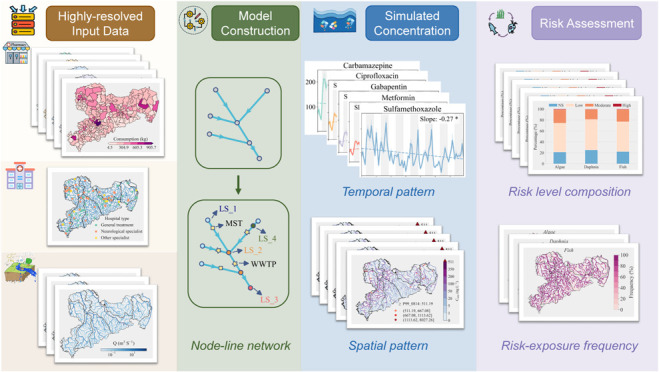

## 1 Introduction

During the last decades, the European Union has launched a series of legislation and strategies on water protection regarding chemical pollution.^1–3^ However, according to one of the latest reports from the European Environmental Agency,^4^ only 38% of surface water bodies in the EU have achieved good chemical status. Of special concern, pharmaceuticals are one of the emerging and concerning chemicals, polluting surface waters in European rivers.^5^ The life stages of human pharmaceuticals involve manufacturing, distribution, consumption, wastewater treatment plant removal, and subsequent entry into the environment. Among their pathways, wastewater treatment plants (WWTPs) are expected to remove pharmaceutical residues to safeguard surface waters.^6^ However, studies indicate that these plants alone cannot fully safeguard the receiving river water.^7–9^ Effluents from WWTPs are documented as the primary contributor to pharmaceutical pollution in surface waters.^4,10,11^ As the world strives for a healthier planet, it is crucial to quantify pharmaceutical pollution in rivers. This is essential for monitoring and improving the resilience of water bodies against chemical pollution.

Process-based modeling is a practical tool for exploring and understanding exposure pathways and characterizing the spatiotemporal distribution of pharmaceuticals in rivers.^12^ Moreover, it addresses the limited availability of monitoring data by extending its applicability across larger spatial domains.^13^ Model-based analyses rely on both accurate representation of environmental processes (e.g., in-stream retention, WWTP removals) and adequate data sets (e.g., consumption patterns, WWTP connections).^12,14,15^ Current models often differ in their representation of environmental processes. For instance, models like HydroFATE^13^ or HydroROUT^16^ use an effective removal rate to represent natural attenuation processes. Recent advancements, such as a study on the Yangtze River, have further disaggregated this overall rate into specific natural attenuation pathways—biodegradation, photolysis, and hydrolysis—to estimate pharmaceutical concentrations.^17^ Other models, including GREAT-ER,^18,19^ ePiE,^20^ STREAM-EU,^21^ and CMFA,^22^ incorporate additional processes like sedimentation and volatilization in their analyses. Yet in common, the above modeling studies utilized annual national-level consumption information as their input data and only focused on the contribution of centralized wastewater treatment plants. The contribution of decentralized wastewater treatment systems (DWTSs) remains less explored.^13,23^ DWTSs generally showed insufficient pharmaceutical removal capability due to a lack of advanced technologies,^24,25^ causing a non-negligible share of contamination from rural dwellings. Applying process-based models at a regional scale (e.g., subnational or river basin level) with detailed representation of processes and improved input data can enable more reliable assessments of pollution levels and associated ecotoxicological risks.^26^

Furthermore, many of the above-mentioned modeling studies have focused on tracing the fate of individual pharmaceuticals and evaluating their toxicity to single species. However, growing evidence suggests that interactions among pharmaceutical mixtures, such as additive, synergistic, or antagonistic effects, can lead to cocktail toxicity, significantly amplifying threats to ecosystems.^27–30^ This underscores the need to consider mixture effects, preferably by applying the conservative concentration addition approach to ensure a precautionary and protective assessment under real-world conditions.^31,32^ Thus, understanding the detailed transport trajectories of pharmaceuticals in rivers and assessing the ecological risks of their mixtures on multiple species over varying temporal scales, such as seasonal variations and long-term dynamics, are crucial for a comprehensive evaluation of pharmaceutical stress on ecosystems. In particular, pharmaceutical residues have shown a long-standing presence in rivers of Saxony, Germany, over the past two decades, raising concerns about ecological risks.^12,33,34^ To address this concern, here, a spatially explicit modeling framework was introduced to track the fate of pharmaceuticals from their sources to receiving environments. This study focuses on five pharmaceuticals (two antiepileptics, two antibiotics, and one antidiabetic) and assesses their fate in Saxon rivers (Germany), covering approximately 10,000 km of river length using data from 2008 to 2014. Weekly spatial consumption data at the postcode level from the primary care sector are used as inputs, supplemented with hospital sector data and emissions from upstream trans-boundary basins. The model considers effluents from both centralized and decentralized wastewater treatment systems. Using the spatially refined modeling framework (≈ 1 km), the ecological risks posed by both single pharmaceutical pollution and the mixture pollution on multiple species (algae, daphnia, and fish) were demonstrated. The findings of this study aim to support the EU’s “Zero Pollution Action Plan”^35^ by assessing the status and risks of pharmaceutical pollution in Saxony rivers, Germany.

## 2 Materials and Methods

### 2.1 Study Area

Our analysis encompasses river stretches located in the eastern federal state of Germany, Saxony, which has nearly 4.08 million inhabitants (in 2022 ^36^). The main river basins in Saxony include the Elbe, Schwarze Elster, Mulde, Weißer Elster, Spree, and Lausitzer Neiße rivers (Figure 1A). There are a total of 700 centralized wastewater treatment plants (WWTPs) in operation and 117,000 decentralized wastewater treatment systems (DWTSs) in Saxony (Figure 1C).^36^ Around 91% of the total inhabitants in Saxony are connected to WWTPs, from which all wastewater is discharged into surface water (Figure 1E). The remaining 9% of inhabitants are connected to DTWSs, from which 80% discharge treated wastewater into surface water and the remaining 20% discharge into groundwater (Figure 1F,G).

**Figure 1:**
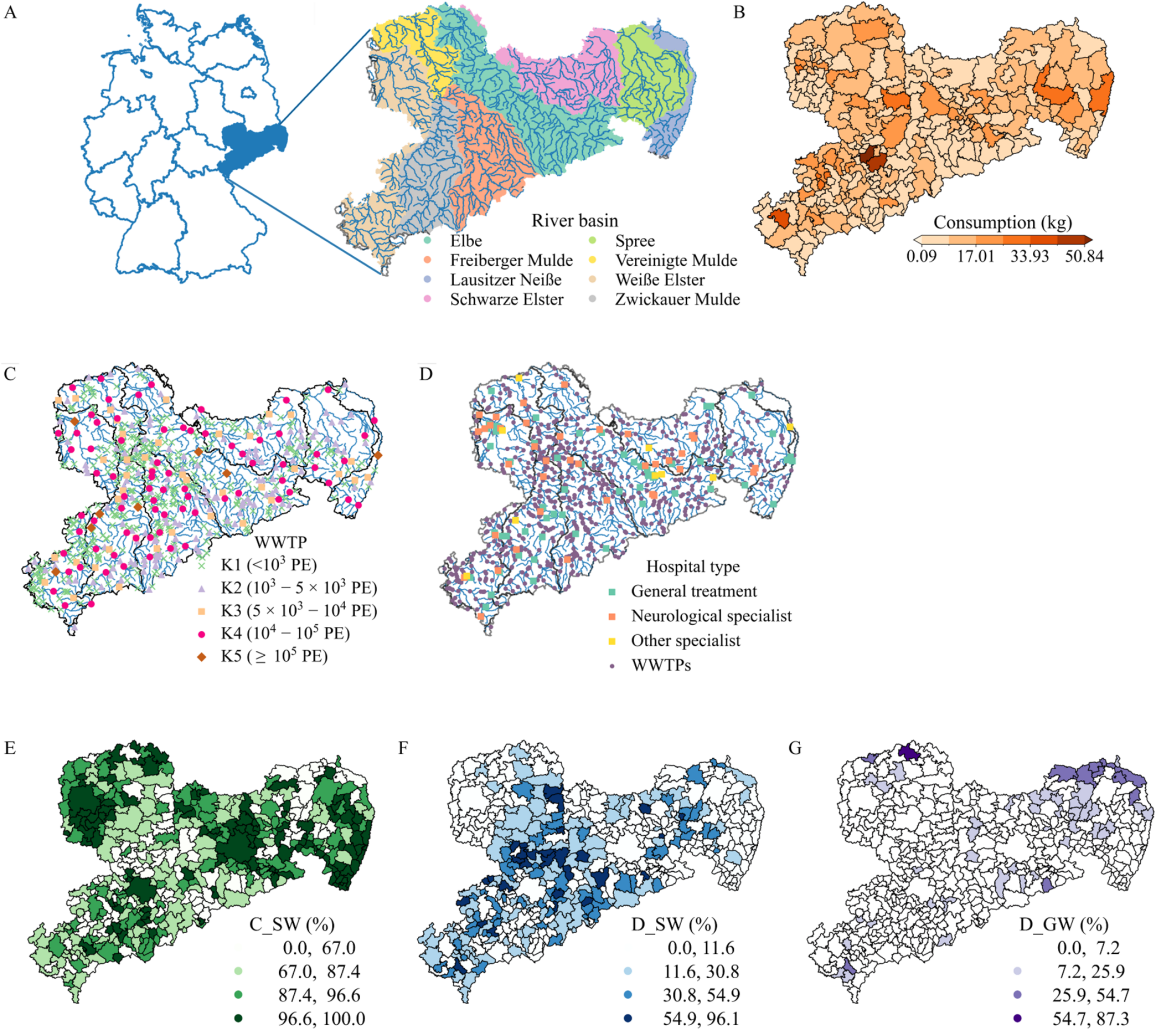
Research area: (A) river basin of Saxony; (B) postcode area of Saxony; (C) locations of WWTPs (centralized wastewater treatment plants, K: class); (D) locations of hospitals; and (E–G) population connectivity to wastewater treatment systems at the spatial resolution of communities. PE: population equivalent. Panels E, F, and G show (E) the percentage of the population connected to WWTPs discharging to surface water (C_SW), (F) the percentage connected to decentralized wastewater treatment systems (DWTSs) discharging to surface water (D_SW), and (G) the percentage connected to DWTSs discharging to groundwater (D_GW). The values in panel B are the averaged annual carbamazepine consumption from the primary care sector at each postcode during 2008 and 2014.

### 2.2 Data Set

All data included in our analysis are listed in Supporting Table S1.

#### 2.2.1 Pharmaceutical Consumption

Five pharmaceuticals were selected here due to their ubiquity and potential toxicity, including two antiepileptics (carbamazepine and gabapentin), two antibiotics (ciprofloxacin and sulfamethoxazole), and one antidiabetic (metformin). Pharmaceutical consumption data for these pharmaceuticals, spanning 2008–2014, were obtained from the “MikroModell” project,^37^ ensuring a comprehensive coverage of Saxony’s postcode area level information (Figure 1B). The data set, originally sourced from the statutory health insurance provider AOK Plus,^38^ includes consumption from both the household (primary care sector) and hospitals (hospital sector). The granularity of this data set enables spatially refined model simulations (see Section 2.3). However, it is limited to 2014 due to its nonpublic nature and data privacy constraints. Comparable data beyond 2015 is unavailable owing to stricter data security policies from AOK Plus.^38^ Although coarser, annual national-level data from WIdO^39^ could be available, it requires disaggregation to both finer temporal (e.g., weekly interval) and spatial (e.g., postcode area) resolutions, which could introduce extra uncertainties and potentially compromise the model’s predictive accuracy. Emissions from upstream trans-boundary basins were also incorporated into the analysis:

(1) **Primary care sector:** household consumption was estimated by combining prescription rates with population demographics by age group (<20, 20–60, ≥ 60 years) to account for the difference between the AOK Plus ensured population and the total population. Prescription rates (grams per 1,000 inhabitants per day) were derived from primary care sector prescription data, available at weekly intervals and postcode resolution (186 areas in Saxony; Figure 1B), and disaggregated to the community level (419 communities) using client number demographics (age groups: < 20, 20–60, ≥ 60 years). To calculate age-specific prescription rates, total prescriptions in each postcode area were first allocated to the three age groups using the following proportions: for metformin, 0.04% (<20 years), 23.57% (20–60 years), and 76.39% (G60 years);^69^ for other pharmaceuticals, 5%, 31%, and 64%, respectively.^70^ Figure S1 shows the corresponding spatial and temporal development of prescription amounts for the analyzed pharmaceuticals in Saxony.
(2) **Hospital sector:** hospital consumption was calculated using monthly consumption rates (grams per bed) and bed counts for each hospital. Data from eight hospitals (2008–2014; bed numbers: 50–1765) were used to interpolate consumption rates, which were applied to 101 hospitals (neurological and general) with georeferenced locations from the German Hospital Directory.^40^ Other specialist hospitals with unrelated treatments were excluded from analysis (shown in yellow in Figure 1D).
(3) **Emissions from upstream trans-boundary basin:** emissions from upstream trans-boundary basins (ubb) included cross-border and interstate river basins (e.g., Thuringia, Saxony-Anhalt, Czech Republic, Poland). Data from 26 upstream trans-boundary stations were provided by the Saxon State Agency for Environment, Agriculture and Geology (LfULG),^36^ with locations referenced to the corresponding river network.^41^ Locations of all upstream trans-boundary stations are presented in Figure S2 and Table S2. Emissions were mainly calculated as,
1 $$E_{\mathrm{ub}b_{i}}=C_{\mathrm{ub}b_{i}}\times Q_{\mathrm{ub}b_{i}}\times 10^{-3}$$
  where *E*_ubb*i*_ is emission (mg s^–1^), *C*_ubb*i*_ is the observed concentration (ng L^–1^), and *Q*_ubb*i*_ is the river discharge (m^3^ s^–1^). However, due to observational data availability, emissions were processed under four different conditions. Details are provided in Supporting Text S1 and Figure S3.

#### 2.2.2 River Network, WWTP Information, and Demographic Data

Saxon river networks were obtained from the LfULG.^36^ Rivers were delineated with a contributing area exceeding 10 km^2^. LfULG^36^ provides the georeferenced locations and designed population equivalent of WWTPs in Saxony (Figure 1C). The served population number of each WWTP, along with the corresponding community information, was obtained by the “MikroModell” project.^37^ Information regarding the population numbers connected to DWTSs and whether they discharged into surface or groundwater was acquired from the LfULG.^36^ Demographic data were acquired from the Statistical Agency of the Free State of Saxony^42^ (see Table S1 for corresponding references of these data sets).

#### 2.2.3 Water Quality Data

The monitoring campaigns were conducted by the LfULG,^36^ with starting years varying for each pharmaceutical: carbamazepine from 2008, gabapentin and sulfamethoxazole from 2010, ciprofloxacin from 2011, and metformin from 2012. Monthly measurements varied depending on the pharmaceutical, ranging from a total of 2989 (metformin) to 12079 (carbamazepine) measurements over the analyzed study period 2008–2014. These measurements were used for the calibration of modeling factors and to evaluate the model performance.

#### 2.2.4 River Discharge

Monthly river discharge data were provided by the mesoscale Hydrologic Model (mHM).^43,44^ mHM is a spatially explicit modeling framework designed to simulate terrestrial hydrological processes like soil moisture, evapotranspiration and runoff at a grid scale, and flow routing in rivers.^45^ mHM has proven effective in regional and large-scale hydrological modeling (see https://mhm-ufz.org and references therein). This study leverages the German-wide mHM setup,^46^ using a spatial resolution of 0.015625° (≈ 1.2 km). The model is driven by gridded meteorological data from the German Weather Service for the period 1980–2018 and parameterized in a regional setting encompassing multiple basins. Extended cross-validation experiments demonstrate the model’s high skill in capturing observed streamflow dynamics, with a median Kling–Gupta efficiency exceeding 0.75 across more than 200 basins.^46^ For further details, see Boeing et al.^46^ and https://mhm-ufz.org. Figure S4 presents the model skill across different river gauges in Saxony. Figure S5 illustrates the mean seasonal discharge across the Saxon rivers along with the monthly variation at selected gauging stations.

### 2.3 Modeling Framework

A geographically informed pharmaceutical model was developed to track their fate from the source to the receiving environment to estimate the riverine concentrations. Figure 2 provides a schematic overview of the modeling framework, outlining the key steps involved in integrating various components of the modeling framework. Four key components are included to comprehensively assess the pharmaceutical fate and ecological risks. First, it links geographically distributed pharmaceutical consumption data to demographic structures and sector-specific distributions (Figure 2b). It accounts for emissions from different sectors, such as hospitals and primary care, and routes them to various emission pathways, including centralized and decentralized wastewater treatment plants. Second, it considers removal processes to quantify how incoming pharmaceutical loads are removed or discharged into river systems based on respective treatment types (Figure 2c). Third, it simulates riverine processes, accounting for varying hydrological conditions and natural attenuation in rivers (Figure 2a). Finally, the framework performs environmental risk assessment to evaluate potential ecological impacts (Figure 2d).

**Figure 2:**
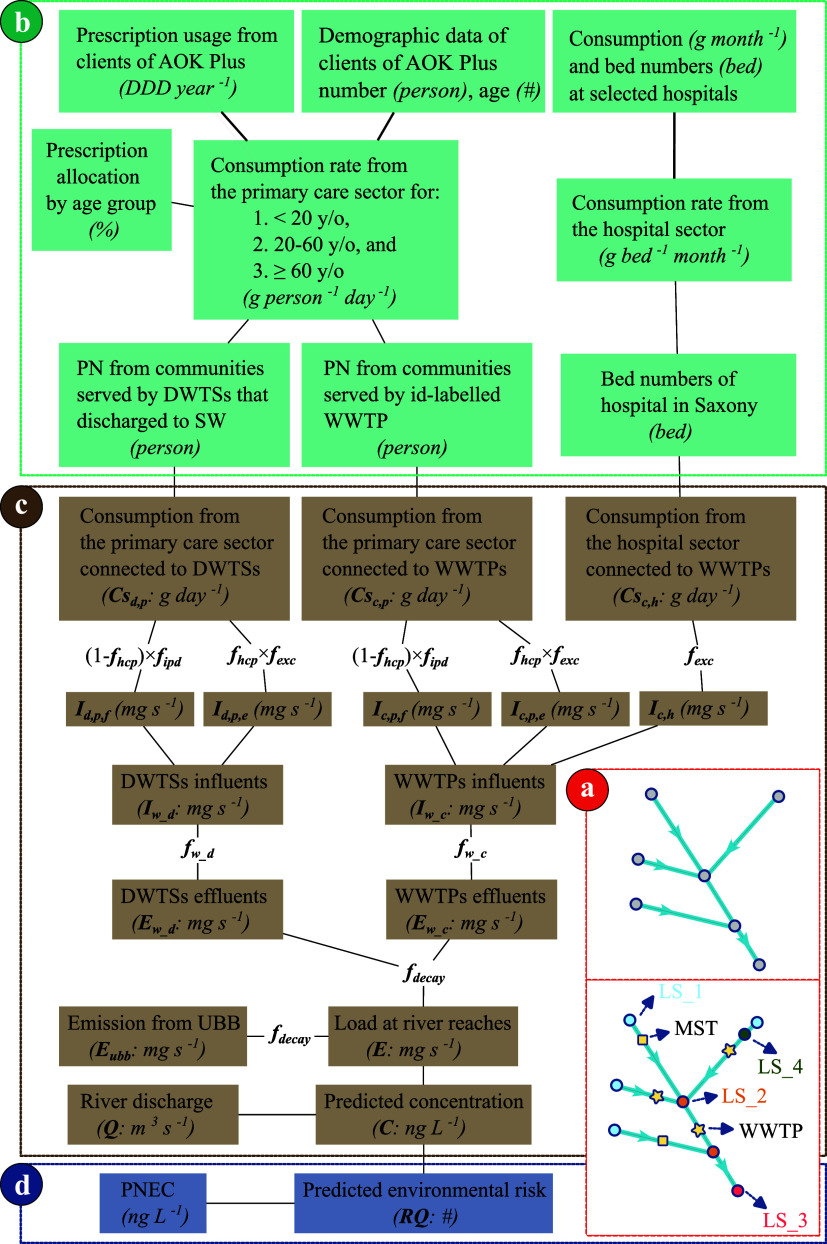
Schematic diagram of the network model development. (a) Conceptual overview of the node-link network; (b) input data; (c) model structure and output data; and (d) risk assessment. LS: linestring; MST: water quality monitoring station; WWTP(s): centralized wastewater treatment plant(s); DDD: daily defined dose; y/o: years old; DWTSs: decentralized wastewater treatment systems; PN: population number; SW: surface water; UBB: upstream trans-boundary basin; PNEC: predicted no-effect concentration; *f*_exc_: human excretion rate; *f*_hcp_: human compliance rate; *f*_ipd_: improper disposal rate; *f*_w_c_: removal efficiency in WWTPs; *f*_w_d_: removal efficiency in DWTSs; *f*_decay_: in-stream decay rates, which includes *f*_bio_: biodegradation rate; *f*_hyd_: hydrolysis rate; *f*_pho_: photolysis rate.

#### 2.3.1 Model Development

The model simulated pharmaceutical concentrations at every 1 km of river segment, resulting in a total of 9136 segments covering the entire Saxon rivers. The river segment is represented as a node-link network (Figure 2a). Each link is connected by two nodes. We classified nodes into three groups: a water quality monitoring station (MST, *n* = 1786), a linestring node (LS, *n* = 6691), and a centralized wastewater treatment plant (WWTP, *n* = 684). Linestring nodes are further divided into four types: (i) LS_1: source node of each river link, including trans-boundary nodes; (ii) LS_2: junction node of links; (iii) LS_3: outlet node of river basin; and (iv) LS_4: the rest of the node type.

In the model, the net emission of pharmaceuticals can be discharged into river node *i* from three sources: effluents from WWTPs (E_w_c_), effluents from DWTSs (*E*_w_d_), and emission from upstream trans-boundary basins (*E*_ubb_) given as

2 $$E_{i}=E_{ubb,i}+E_{w\_c,i}+E_{w\_d,i}$$

$$E_{w\_c}=I_{w\_c}\times f_{w\_c}=\sum \left(I_{c,p,e}+I_{c,p,f}+I_{c,h}\right)\times f_{w\_c}$$ 3

$$E_{w\_d}=I_{w\_d}\times f_{w\_d}=\sum \left(I_{d,p,e}+I_{d,p,f}\right)\times f_{w\_d}$$ 4

$$I_{x,p,e}=Cs_{x,p}\times f_{hcp}\times f_{exc}\;(x=c\;or\;d)$$ 5

6 $$I_{x,p,f}=Cs_{x,p}\times \left(1-f_{hcp}\right)\times f_{ipd}\;\left(x=c\;or\;d\right)$$

7 $$I_{c,h}=Cs_{c,h}\times f_{\mathrm{exc}}$$

where *E*_w_c_ and *E*_w_d_ are the net emissions (effluents) from WWTP and DWTS, respectively (mg s^–1^); *I*_w_c_ and *I*_w_d_ are the gross emissions (influents) from WWTP and DWTS, respectively (mg s^–1^); *I*_c,p,e_ is the influent from the consumed (eaten) pharmaceuticals in the primary care sector to the WWTP node (mg s^–1^); *I*_c,p,f_ is the influent from the flushed pharmaceutical in the primary care sector to the WWTP node (mg s^–1^); *I*_c,h_ denotes the influent from the pharmaceutical in the hospital sector to the WWTP node *(*mg s*^–1^*), which is considered entirely consumed without any improper disposal; *I*_d,p,e_ is the influent from the consumed pharmaceuticals in the primary care sector to the DWTS node (mg s^–1^); *I*_d,p,f_ is the influent from the flushed pharmaceutical in the primary care sector to the DWTS node (mg s^–1^); Cs_*x*,p_ is the consumption from the primary care sector to *x*, which represents *c* (WWTP node) or *d* (DWTS node) (mg s^–1^). Cs_c,h_ is the consumption from the hospital sector to the WWTP node, which is connected only to WWTPs. *f*_exc_ represents the excretion rate from the human body (unitless, −); *f*_hcp_ is the human compliance rate (−); *f*_ipd_ is the improper disposal rate (−), which denotes the percentage of unused prescribed pharmaceuticals that are being flushed into the sink or toilet (−); and *f*_w_c_ and *f*_w_d_ are the removal efficiencies from WWTPs and DWTSs, respectively (both −). Further, we also considered DWTSs that release treated wastewater into surface waters.

The pharmaceutical load at node *i* comprises two components: (1) the net emission discharged from the WWTP and/or DWTS and/or upstream trans-boundary basin (ubb) directly into node *i* and (2) the accumulated emissions from all upstream nodes *j* ∈*U*(*i*), which undergo in-stream decay during their transport to node *i*. During the river transport process, such as from node *j* to node *i*, pharmaceutical residues experience (first-order) attenuation through biodegradation (*f*_bio_), hydrolysis (*f*_hyd_), and photolysis (*f*_pho_). The resulting concentration of a given pharmaceutical at node *i* can be expressed as

8 $$C_{i}=\frac{E_{i}+\sum_{j\in U\left(i\right)}E_{j}\cdot e^{-\left(\sum_{m=1}^{3}k_{m,d_{j-i}}\right)\cdot d_{j-i}/v_{d_{j-i}}}}{Q_{i}}\times 10^{3}$$

where *C*_*i*_ is the concentration at node *i* (ng L^–1^); *E*_*i*_ is the net emission discharged from WWTP and/or DWTS and/or ubb to node *i* (mg s^–1^); $E_{j}\cdot e^{-\left(\sum_{m=1}^{3}k_{m,d_{j-i}}\right)\cdot d_{j-i}/v_{d_{j-i}}}$ is the decayed upstream emission from node *j*, which is upstream from node *i* (mg s^–1^); *U*(*i*) is the set of all upstream nodes of node *i*; *d*_*j*–*i*_ is the river length for the link from node *j* to node *i* (*m*); v_*d*_*j*–*i*__ is the flow velocity over the distance *d*_*j*–*i*_ (m s^–1^); *k*_*m*_ are the decay rates, which include biodegradation, hydrolysis, and photolysis (s^–1^); *Q*_*i*_ is the river discharge at node *i* (m^3^ s^–1^). v_*d*_*j*–*i*__ was calculated using the Manning equation.^47^ The required landscape information (e.g., channel slopes) was retrieved from respective elevation data (see Table S1 for details). Further, given the lack of geoinformation on DWTSs, we assumed every modeling node gets contributions from DWTS, with the constraint that aggregated DWTS contributions for each pharmaceutical at the community level align with the corresponding consumption data.

The presented model considers eight unknown factors, which are categorized into two main groups: five anthropogenic factors and three environmental factors (see Table S3 for details). For instance, removal efficiencies such as *f*_w_c_ and *f*_w_d_ are considered anthropogenic factors, while in-stream decay rates (*f*_bio_, *f*_hyd_, and *f*_pho_) represent environmental factors. We examine the sensitivity of these factors affecting the space–time behavior of modeled concentrations using a global sensitivity analysis^48^ (see Supporting Text S2 for a detailed discussion).

#### 2.3.2 Model Parameterization and Evaluation

A stepwise calibration method was implemented to reduce the interactions between anthropogenic and environmental factors. We therefore first estimated the three environmental factors (decay rates). Then, we fixed the environmental factors and calibrated the five anthropogenic factors. Initial ranges for these environmental factors for the five pharmaceuticals were obtained from previous studies (Table S3). The calibration was conducted separately for the cool season (November–April) and the warm season (May–October) to reflect the underlying seasonal variability.^49^ We used measured concentrations from two consecutive monitoring stations without WWTPs in between for estimating environmental factors as

9 $$E_{1}=E_{0}\times \frac{d_{0-1}}{v_{d_{0-1}}}\times \sum_{m=1}^{3}k_{m,d_{0-1}}$$

10 $$E_{i}=C_{i}\times Q_{i}\times 10^{-3}$$

where *E*_0_ and *E*_1_ are the mass loads at the upstream and downstream monitoring stations, respectively (mg s^–1^). *C*_*i*_ is the measured concentration at the monitoring station *i* (ng L^–1^). *Q*_*i*_ is the river discharge at the station *i* (m^3^ s^–1^). *d*_0–1_ is the river length between the upstream and downstream stations (*m*). v_*d*_0–1__ is the river flow velocity between them (m s^–1^). The total number of paired stations ranged from 4 for ciprofloxacin to 53 for carbamazepine, with the respective number of paired measurements ranging between 12 and 246. The data at the paired stations were further quality-controlled for plausibility, such that the emission at a downstream station (*E*_1_) is lower than that of the corresponding upstream station (*E*_0_).

Next, the anthropogenic factors (Table S3) were estimated in a regional setting by applying the model across the entire study region. The three environmental factors, established in the previous step, were fixed, and the model was then run iteratively, refining the anthropogenic factors until achieving a solution that provided the best regional model performance. Depending on the pharmaceutical, the number of measurement stations ranged from 118 (ciprofloxacin) to 611 (carbamazepine), resulting in the total number of measurements varying, respectively, between 198 and 2784.

The model performance was evaluated by comparing simulated concentrations (*C*_sim_) with observed concentrations (*C*_obs_) using two metrics: the Spearman’s rank correlation coefficient (ρ) and the normalized root-mean-square error (nRMSE). Unlike the Pearson correlation, ρ is a nonparametric correlation measure that is robust against outliers. To ensure comparability across different pharmaceuticals, a scale-independent nRMSE, calculated based on respective concentration ranges, is used to describe model performance. We also report the percentage of simulation points falling within 1 order of magnitude of the observations to reflect the overall well-fitted behavior of model simulation. A 1-order-of-magnitude boundary in modeling error could be considered as acceptable performance, given the inherent uncertainties in model simulations arising from inputs such as prescriptions, WWTP removal efficiencies, discharge estimates, and observational records. Furthermore, such a boundary is widely used in risk assessment (e.g., risk quotient; see Section 2.4) to classify chemical risks and guide monitoring priorities. Therefore, allowing for 1 order of magnitude accommodates inherent uncertainties while maintaining risk classification accuracy, supporting informed decision-making based on model outputs.

### 2.4 Environmental Risk Assessment

The risk quotient (RQ) method^50–52^ was used to assess the environmental risk of aquatic ecosystem to single chemicals (eqs 11 and 12). Three aquatic species groups, namely, algae, daphnia, and fish—representing primary producers, primary consumers, and secondary consumers, respectively^53^ were assessed for ecological risk

11 $$\mathrm{RQ}=\frac{\mathrm{PEC}}{\mathrm{PNEC}}$$

12 $$\mathrm{PNEC}=\frac{EC_{50}}{\mathrm{AF}}$$

where RQ is the risk quotient values (−), PEC is the predicted environmental concentration (ng L^–1^), PNEC is the predicted no-effect concentration (ng L^–1^), AF is the assessment factor, adopted here as 1000 for the acute toxicity,^50^ and EC_50_ standing for the median effect concentration were adopted from the peer-reviewed paper,^54^ which is based on a curated data set of effect concentrations. The corresponding values are listed in Table S4.

Next, the mixture toxicity (RQ_mix_) was estimated as the additive effect of mixture chemicals, assuming that the five target pharmaceuticals (*n* = 5) share a similar mode of action^55,56^

13 $$RQ_{\mathrm{mix}}=\sum_{i=1}^{n}RQ_{i}=\sum_{i=1}^{n}\left(\frac{\mathrm{PE}C_{i}}{E{C_{50}}_{i}\times \frac{1}{AF_{i}}}\right)$$

Finally, we classified the ecological risks into four tiers,^51,52^ namely, nonsignificant risk, low risk, moderate risk, and high risk, with the range of RQ < 0.01, 0.01 ≤ RQ < 0.1, 0.1 ≤ RQ < 1, and RQ ≥ 1, respectively. Despite the nonconcurrent monitoring of the pharmaceuticals, single and mixture toxicity assessments relied on simulated concentrations spanning 2008–2014. This approach was necessary to overcome gaps in observational data and enable a more comprehensive temporal analysis.

## 3 Results and Discussion

### 3.1 Model Performance

Figure 3 depicts the model skill in terms of Spearman’s rank correlation coefficients (ρ) and normalized root-mean-square errors (nRMSEs) for the five analyzed pharmaceuticals over the period 2008–2014. The performance evaluation confirmed that the model captures the spatial and temporal gradients of pharmaceutical concentrations across Saxon river basins at an acceptable level despite some outliers. ρ values ranged from 0.73 (ciprofloxacin) to 0.88 (gabapentin), indicating strong simulation–observation agreement for estimating environmental (in-stream decay) factors (Figure 3A; refer to Figures S6–S9 for details on model train-and-test performance, excluding ciprofloxacin due to limited training data). nRMSE values were 0.50, 0.08, 0.19, 3.63, and 0.15 for carbamazepine, ciprofloxacin, gabapentin, metformin, and sulfamethoxazole, respectively, indicating deviations of 50%, 8%, 20%, 363%, and 15%. However, removing an outlier from the observational metformin data set reduced nRMSE substantially from 3.63 to 0.75, improving model reliability. In general, 97% of simulations were within 1 order of magnitude of observations, with ciprofloxacin achieving 100% and metformin 95%.

**Figure 3:**
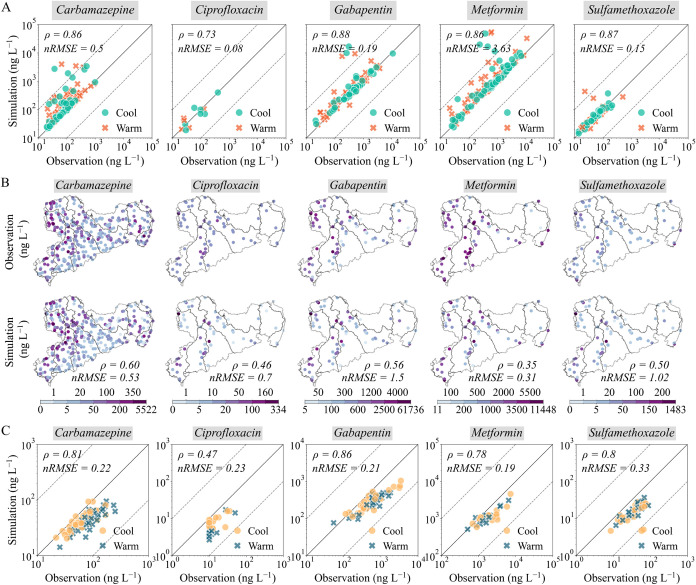
Performance evaluation of the model simulations corresponding to the estimation of environmental (in-stream decay) factors (A), of all model factors across space (B), and of all model factors over time (C). The solid line represents the 1:1 line, and the dashed lines represent a difference of 1 order of magnitude. ρ and nRMSE represent Spearman’s rank correlation coefficient and the normalized root-mean-square error between model simulations and observations, respectively. All correlation coefficients are at the *p*-value < 0.01 significance level.

Figure 3B,C evaluates model performance spatially and temporally, incorporating both modeling factors. Spatially, ρ values ranged from 0.35 (metformin) to 0.60 (carbamazepine) and nRMSE ranged from 0.31 (metformin) to 1.50 (gabapentin). The main Elbe River basin performed best with a ρ of 0.79, an nRMSE of 0.08, and a 94% well-fitted coverage, while a lower but still good coverage (>73%) was observed for the Lausitzer Neiße River (Table S5). Temporally, ρ values varied from 0.47 (ciprofloxacin) to 0.86 (gabapentin), with the nRMSE ranging from 0.19 (metformin) to 0.33 (sulfamethoxazole). Almost all pharmaceuticals achieved a 100% well-fitted coverage, except sulfamethoxazole (98%).

In summary, ciprofloxacin exhibited lower performance, likely due to its low environmental concentrations^20^ 73% of space-harmonized and 78% of time-harmonized concentrations at or near the limit of quantification (10 ng L^–1^; Figure 3B,C). In contrast, carbamazepine showed a consistent and stable performance in both space and time, likely supported by the greater availability of observations. Metformin demonstrated high temporal performance but low spatial correlation due to underestimations in upstream regions of the Zwickauer Mulde River (Figure 3B,C), highlighting the need for additional sampling in smaller streams. Despite discrepancies, overall, the model effectively captured the observed spatial and temporal behavior of in-stream pharmaceutical concentrations.

### 3.2 Spatiotemporal Evolution of Pharmaceutical Occurrence

Figure 4A illustrates the spatial evolution of flow-weighted mean pharmaceutical concentrations (*fwmc*) during 2008–2014 across Saxon rivers. Median fwmc were 27 ng L^–1^ (p05–p95:0.5–180 ng L^–1^), 7.7 ng L^–1^ (0.2–78.9 ng L^–1^), 218.3 ng L^–1^ (5.7–19,18.4 ng L^–1^), 516.5 ng L^–1^ (13.6–3285.8 ng L^–1^), and 10.3 ng L^–1^ (0.3–101.4 ng L^–1^) for carbamazepine, ciprofloxacin, gabapentin, metformin, and sulfamethoxazole, respectively. Regionally, the Zwickauer Mulde River exhibited the highest fwmc for most pharmaceuticals except metformin (Figure S10), consistent with its high reliance on DWTS (second-highest DWTS connection rate; Figure S11). This implies a relatively higher contribution of net pharmaceutical emissions from DWTS in this river basin. Wiegel et al.^33^ investigated the contamination levels of pharmaceuticals in the Elbe River and reported the highest concentration in the Vereinigte Mulde River in 2004, indicating a shift in the hotspots of pharmaceutical pollution. The Lausitzer Neiße River had the lowest concentrations for most pharmaceuticals, except ciprofloxacin, which was lowest in the Spree River. The Neiße River flows along the Poland–Germany boundary (Figure 1A), and the lack of a detailed Polish contributing basin may have led to underestimations of pharmaceutical effluents.

**Figure 4:**
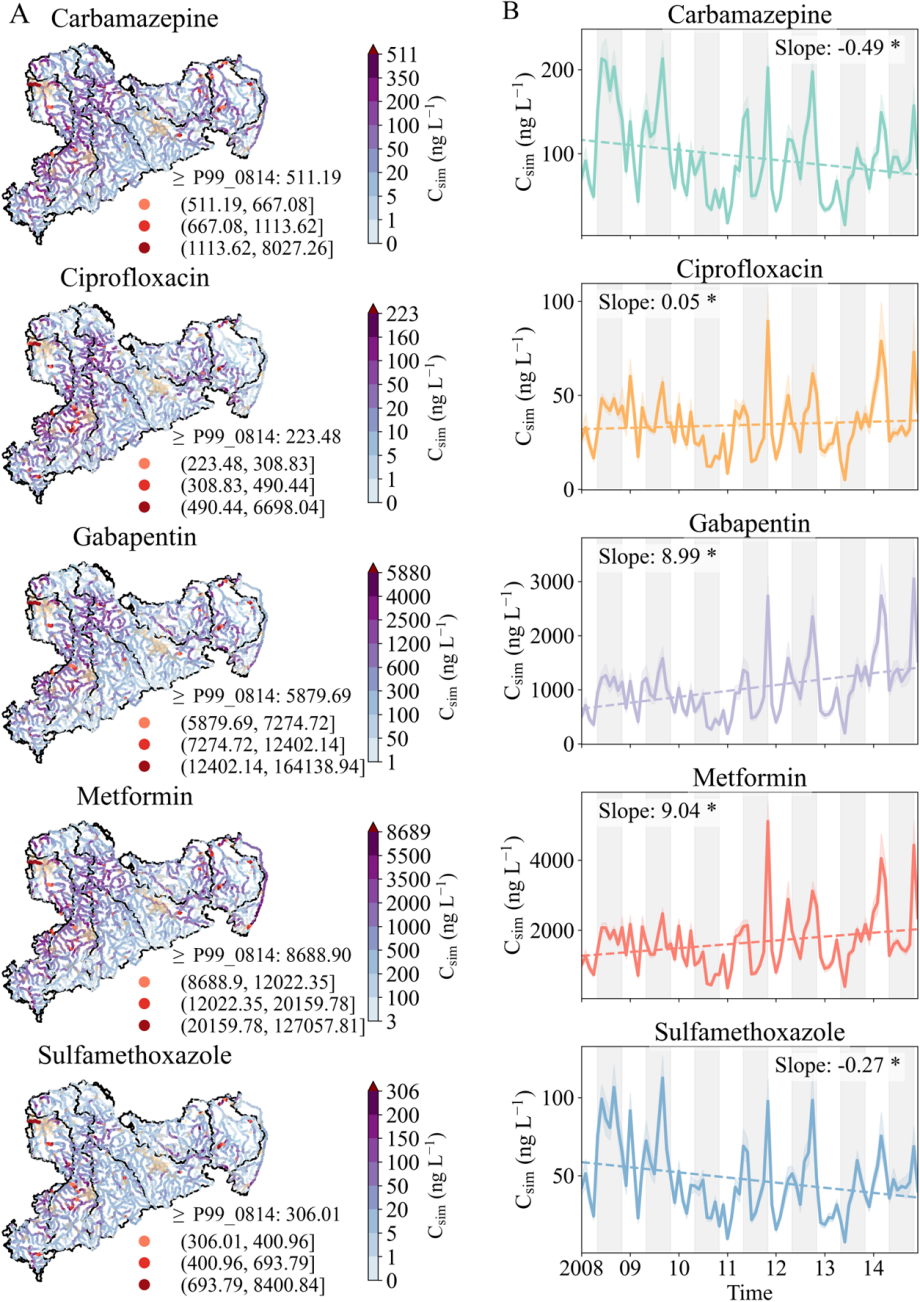
Spatial distribution (A) and temporal trend (B) of simulated concentrations (*C*_sim_) in Saxon rivers. In panel A, P99_0814 was provided to indicate extreme concentrations, which represents the 99th percentile of the simulations between 2008 and 2014. The orange-shaded areas are populated urban regions from the LfULG.^36^ In panel B, the dashed line indicates the fitted linear trend in the respective pharmaceutical concentration. Areas without a background color represent cool seasons (from November to April), while gray-shaded background areas represent warm seasons (from May to October). Significant linear trend slopes (*C*_sim_: ng L^–1^ month^–1^) are indicated with * at *p-*value < 0.01.

Figure 4B shows the temporal dynamics of simulated pharmaceutical concentrations estimated across the Saxon rivers. Concentrations displayed strong variability, ranging from <100 to >200 ng L^–1^ for carbamazepine and from <100 to >2000 ng L^–1^ for gabapentin over the study period (2008–2014). Significant temporal trends were observed: carbamazepine concentrations decreased (−0.49 ng L^–1^ month^–1^), while gabapentin (8.99 ng L^–1^ month^–1^) and metformin (9.04 ng L^–1^ month^–1^) concentrations increased. This trend aligns with that of gross Saxon-wide emissions to WWTPs and DWTSs (Figure S1), suggesting that prescription or consumption can provide an indication of the likely trend for in-stream concentrations of gabapentin, more strongly compared to those of carbamazepine. The increasing trend for metformin is also consistent with its increasing consumption during the study period (Figure S1). The Saxon-wide ciprofloxacin concentrations increased slightly (0.05 ng L^–1^ month^–1^) despite reduced consumption, while sulfamethoxazole concentrations decreased (−0.27 ng L^–1^ month^–1^) over time. Overall, these analyses highlight significant variations in pharmaceutical distribution across the Saxon rivers and over time. Furthermore, the increasing concentrations of gabapentin and metformin highlight the need for long-term, substance-specific monitoring, especially in hotspot areas identified by the model, to support timely management interventions and inform future regulatory frameworks.

### 3.3 Ecological Risk Assessment

Figure 5A and Table S6 show the composition of rivers exposed to different risk levels from single and mixture pharmaceutical pollution. Algae were the most sensitive species to pharmaceutical mixtures, with 77% of rivers at-risk (low, moderate, and high) in the cool season and 80% in the warm season. For single toxicity, the percentage of at-risk rivers for algae ranged from 6% (sulfamethoxazole) to 59% (gabapentin) in the cool season and from 10% (ciprofloxacin, sulfamethoxazole) to 60% (gabapentin) in the warm season. Fish showed similar risks from pharmaceutical mixtures (76% of rivers in the cool season and 80% in the warm season). For single toxicity, at-risk rivers ranged from 11% (ciprofloxacin, sulfamethoxazole) to 60% (metformin) in the cool season and from 14% (ciprofloxacin) to 63% (metformin) in the warm season. Daphnia was less vulnerable to pharmaceutical mixtures, with 73% of rivers at-risk in the cool season and 77% in the warm season. For single toxicity, at-risk rivers ranged from 15% (ciprofloxacin) to 49% (gabapentin) in the cool season and from 17% to 51% in the warm season. For all species, moderate-to-high-risk river segments from single pharmaceuticals ranged from 0 to 11%, but this increased significantly for pharmaceutical mixtures (17–24% in the cool season and 21–28% in the warm season; Figure 5A). This highlights the amplifying risks associated with mixture pharmaceutical pollution.

**Figure 5:**
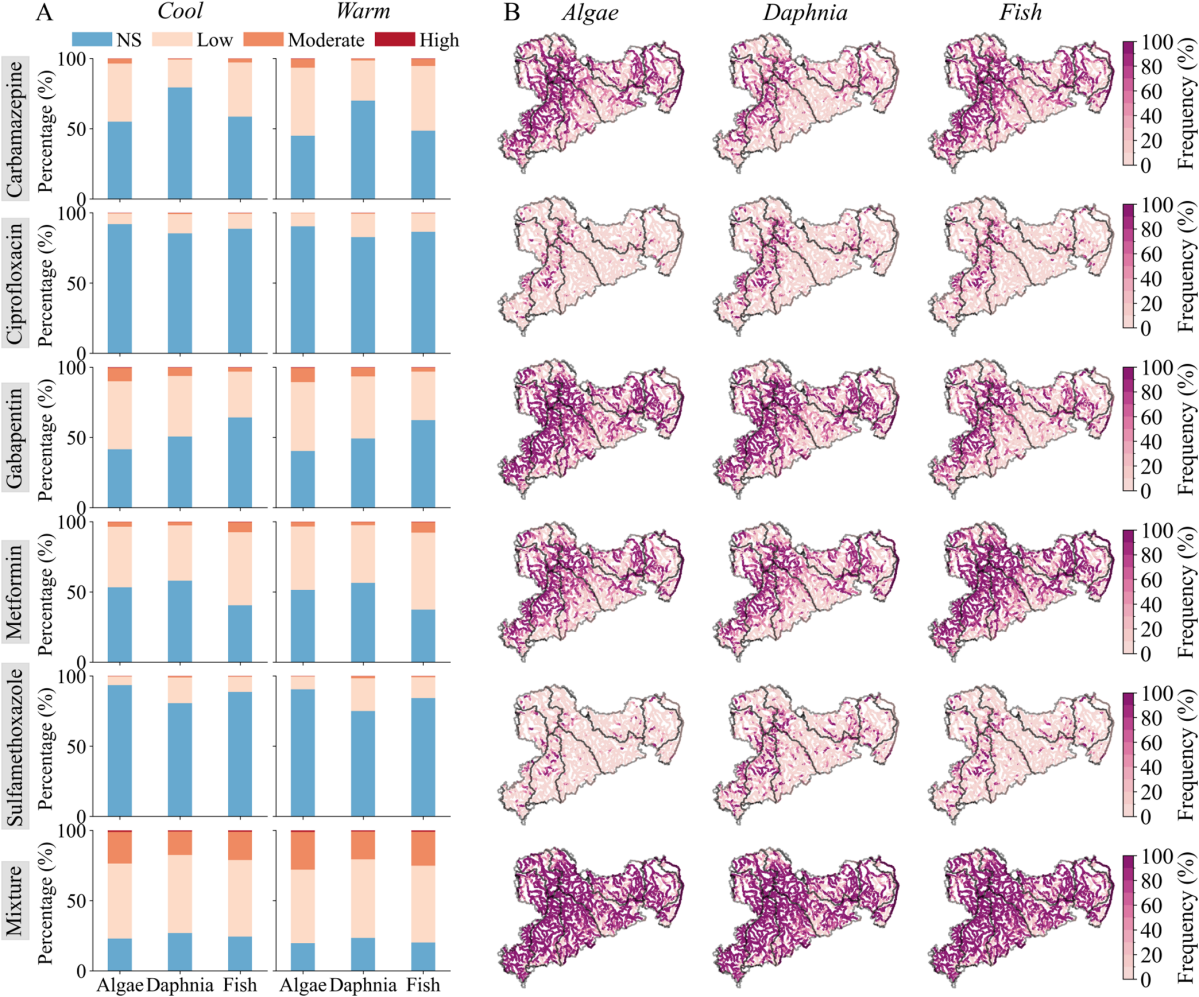
(A) Percentage of river segments exposed to four ecological risk levels for three species for the warm and cool seasons; (B) spatial distribution of the frequency of at-risk ecological toxicity caused by single pharmaceuticals and their mixture. Values shown in panel (A) represent seasonal mean percentages based on monthly simulations (2008–2014); corresponding uncertainty estimates (95% confidence intervals) are provided in the Supporting Information (Text S4, Table S7, and Figure S13). The frequency in panel (B) represents at-risk (aggregation of low, moderate, and high risk) as a proportion of the total simulation time series, excluding nonsignificant cases. NS, low, moderate, and high risks were classified with respective thresholds of 0.01, 0.1, and 1 times the predicted no-effect concentration (PNEC), respectively.

The Tukey HSD test revealed seasonal differences in risk quotient values. Carbamazepine and sulfamethoxazole posed higher risks to algae in the warm season than in the cool season (*p-*value < 0.01), while ciprofloxacin and metformin were more toxic in the cool season (*p-*value < 0.01). Gabapentin showed no significant seasonal differences. The seasonal risk patterns for algae were consistent with those for daphnia and fish. However, mixture toxicity was significantly higher in the warm season for algae (*p-*value < 0.01) but showed no significant difference for daphnia and fish. This seasonal difference is mainly driven by hydrological conditions, as supported by both the sensitivity analysis and the Damköhler number evaluation (Supporting Text S3), which consistently demonstrated that river discharge had a stronger influence on pharmaceutical concentrations than temperature-dependent degradation processes (i.e., biodegradation and photolysis).

Spatially, Figure 5B and Table S8 show the frequency of at-risk rivers for each pharmaceutical and mixture, based on monthly simulations from 2008 to 2014. At-risk exposure frequencies increased downstream, reflecting the accumulation behavior. Gabapentin posed the greatest single-toxic risk to algae (median 76%) and daphnia (55%), while metformin posed the highest risk to fish (median 80%). Mixture toxicity was widespread, exposing 99%, 96%, and 98% of rivers to risks for algae, daphnia, and fish, respectively (Figure 5B; bottommost panel). Regionally, the Zwickauer Mulde River showed the highest exposure frequency for most pharmaceuticals and mixtures, while metformin’s highest exposure frequency occurred in the Weiße Elster subcatchment. Even in the least polluted subcatchments (Lausitzer Neiße), mixture toxicity remained high (93–96%), posing widespread chronic risks to the aquatic ecosystem. These findings highlight the importance of assessing both single and mixture toxicities, as interactions among pharmaceuticals can amplify adverse effects.^27,57,58^ Regarding the single toxicity, our results show that metformin poses the greatest risk to fish, while gabapentin has the highest impact on algae and daphnia. Ciprofloxacin and sulfamethoxazole pose limited risks to the ecosystem. Mixture toxicity significantly increases ecological risks, emphasizing the need for stronger regulatory measures.

The risk estimates discussed above are based solely on toxicity and assume additive effects for mixture toxicity. For a more comprehensive assessment, additional factors, such as bioaccumulation and other nonadditive interactions, including synergistic or antagonistic effects^29,30^ should also be considered. However, data on these aspects are often scarce. To address this, we conducted a preliminary risk assessment incorporating four criteria: occurrence (O), persistence (P), bioaccumulation (B), and toxicity (T).^59,60^ Details of this OPBT-score method and results are provided in Supporting Text S5. While the main conclusion remains unchanged that the mixture consistently posed higher risks to the ecosystem compared to single pharmaceuticals, presenting results from two complementary approaches highlights the importance of examining ecological risk from multiple perspectives (see Text S5 and resulting analysis for details).

Another consideration is the choice of predicted no-effect concentration (PNEC) values. We used data-model-curated effect concentrations available for all pharmaceuticals and species considered. While this ensures coverage, it may underestimate the actual risks. A sensitivity analysis using the fifth percentile of experimental effect concentrations (Figure S12) showed only minor differences in identifying at-risk regions or time periods. Nonetheless, more consistent experimental effect concentration data are needed to improve future PNEC derivation.

Considering initiatives, such as the EU’s “Zero Pollution Action Plan”, which aims to achieve a toxic-free environment, and the “Water Framework Directive”, which seeks to ensure good chemical and ecological status,^1,35^ the current pollution levels in Saxony fall short of these targets. It underscores the importance of strengthening regulations to protect ecosystems from pharmaceutical pollution.

## 4 Limitations, Improvements, and Implications

In this study, we developed a spatially refined pharmaceutical model to track the fate of five pharmaceuticals from their sources to receiving rivers across Saxony, Germany. Unlike previous models focusing on small-scale hydraulic and detailed landscape components^61,62^ or large-scale analyses with coarse input data,^20,63^ our approach balances spatial extent and data resolution (see Table S9). By leveraging highly resolved input data and simplifying some environmental processes, we analyzed the spatiotemporal behavior of pharmaceuticals across river networks, bridging the gap between local and large-scale analyses. Simplifying processes, however, required assumptions, which likely contributed to potential discrepancies between the simulations and observations. First, due to missing geoinformation, 8–12% of emissions from DWTSs were uniformly distributed across river nodes, potentially introducing spatial bias. To assess this, scenario analyses (no DWTS and discharge augmented DWTS) were conducted and compared to the uniform distribution results. The findings underscore the importance of including DWTS emissions in riverine pharmaceutical fate modeling and highlight the influence of their spatial allocation on local concentration patterns (Supporting Text S6). Second, emissions from neighboring states or countries were estimated via interpolation or from concentration detection limits where monitoring stations were unavailable, adding uncertainty to trans-boundary river inputs. Third, calculating mass loads by combining grab sampling concentrations with monthly mean river discharge can lead to under- or overestimations, especially during extreme hydrological events. Finally, modeled river discharge, despite using a well-established hydrological model, carries its own uncertainties (Figures S4 and S5).

Future improvements could address input data quality and model refinement. For instance, (1) acquiring detailed DWTS locations would improve resolution of emissions, especially in rural areas (see Supporting Text S6 for details on the scenario analysis on DWTS locations); (2) adopting removal efficiencies specific to wastewater treatment levels would better reflect net emissions,^64,65^ particularly for pharmaceuticals sensitive to WWTP performance (see Supporting Text S7 for details on the scenario analysis using size-specific removal rates), and further highlights the need for broader monitoring and reporting of removal efficiency; (3) enhancing monitoring strategies with regular, long-term measurements and cooperative monitoring for cross-border rivers would reduce input data uncertainties^66^ and increase model robustness; (4) refining the model to include solar radiation effects on in-stream decay rates, as well as sedimentation and volatilization, would allow for more refined simulations.^21,22^ However, incorporating additional processes also necessity relevant data (e.g., pharmaceutical residues in streambed) to establish robust model parameterizations; and (5) incorporating pharmaceutical legacy in rivers, such as those persistent in water^67^ or exhibiting high sediment sorption,^68^ could enhance the understanding of pharmaceutical fate in river systems.

Despite the mentioned challenges, our modeling framework successfully captured the spatial and temporal variations in pharmaceutical concentrations and their cocktail effects on ecological species. In this first regional assessment, despite being limited to five pharmaceuticals, we demonstrated the widespread prevalence of ecotoxicological risks from pharmaceutical mixtures, highlighting the need to complement single toxicity evaluations with mixture toxicity assessments for a comprehensive understanding of environmental risks.^57,58^ Further, the spatially resolved risk maps presented here could serve as a practical tool to inform management strategies, such as identifying pollution hotspots (e.g., in the Zwickauer Mulde River basin) and locations for new infrastructure or upgrading existing WWTPs to combat pharmaceutical pollution. Our presented model, while applied to five pharmaceuticals from diverse therapeutic classes, is designed to be broadly adaptable through chemical-specific parameterization of both environmental and anthropogenic factors. Its modular node-link structure allows for the extension to other pharmaceuticals, supporting its use as a generalizable framework for fate modeling in river systems across different geographic regions and time periods. To this end, coupling the pharmaceutical fate module with hydrologic model outputs (e.g., continuous discharge simulations) provides a valuable tool for investigating the emerging risk of pharmaceutical pollution under changing hydroclimatic and socioeconomic conditions, including demographic and economic shifts. The framework can support assessments related to environmental goals outlined in the EU’s “Zero Pollution Action Plan”,^35^ identifying pollution gaps and informing proactive actions to achieve a toxic-free environment for future generations.

## Supplementary Material



## Notes

Additional methodological details, supporting analyses such as global sensitivity analysis, scenario analyses on the allocation of decentralized wastewater treatment systems, scenario analyses on centralized wastewater treatment performance, and relevant supporting tables and figures (PDF)
