# Supplementary material for: Spatially and Seasonally Resolved Predictions Reveal Widespread Ecotoxicological Risk from Pharmaceutical Mixtures in German (Saxon) Rivers
Source: Environ Sci Technol. 2025 Aug 15;59(33):17722–34. doi: 10.1021/acs.est.5c01639 (PMC12392725; doi:10.1021/acs.est.5c01639)
Supplement: Supplementary file 1 [file es5c01639_si_001.pdf]

Supporting Information for "Spatially and Seasonally Resolved Predictions Reveal Widespread Ecotoxicological Risk from Pharmaceutical Mixtures in German (Saxon) Rivers" †

Shixue Wu,*,‡,¶ Björn Helm,¶ Geovanni Teran-Velasquez,¶ Peter Krebs,¶ and Rohini Kumar*,‡

‡*Department of Computational Hydrosystems, Helmholtz Centre for Environmental Research–UFZ, 04318 Leipzig, Germany*

¶*Institute of Urban and Industrial Water Management, Technische Universität Dresden, 01069 Dresden, Germany*

E-mail: shixue.wu@ufz.de; rohini.kumar@ufz.de

†**Summary:** 73 Pages, 42 Figures, 10 Tables.

1 Supporting Texts

1.1 Text S1: Emission from Upstream Trans-boundary Basin

Pharmaceutical emissions from 26 upstream trans-boundary stations (Figure S2) during the period 2008–2014 were estimated based on the availability of pharmaceutical concentration data, following four methodological pathways:

(i) **LOQ – No data due to non-monitoring station:** For trans-boundary stations not included in the water quality monitoring network of the Saxon State Agency for Environment, Agriculture and Geology (LfULG),[1] no direct observations were available. In these cases, concentrations were substituted using half of the parameter-specific limit of quantification (LOQ), as provided by LfULG[1].

(ii) **OBS – Observed data available:** When a trans-boundary station belonged to the LfULG monitoring network and had valid measurements for the target month, observed concentrations and simulated river discharge were directly used in the emission calculation.

(iii) **INT – Interpolation using basin-specific ratios:** For LfULG monitoring stations lacking data in a specific month, but where the year fell within or after the pharmaceutical's monitoring period, concentrations were interpolated using a basin-informed ratio method. Two long-term reference stations were selected: OBF00200 (Elbe River, S11 in this study) and OBF49500 (Weiße Elster River, S5 in this study). Each monitoring station was assigned to one of these basins based on spatial metadata.

For each pharmaceutical, monthly observed concentrations at individual monitoring stations were aggregated into seasonal means for each year, with cool (November–April) and warm (May–October) periods representing seasonal variability. If a station

lacked all data for a given pharmaceutical within a specific season and year, concentrations were substituted using the pharmaceutical- and date-specific LOQ; this procedure corresponds to and is named to the "LOQ" method.

For stations with partial data, missing values were interpolated using year- and season-specific mean concentrations, based on average concentration ratios relative to the appropriate reference station. For stations located within the Elbe or Weiße Elster basin, the ratio was calculated against the corresponding reference station for each year and season. For stations outside both basins, interpolations were performed independently using ratios to both reference stations, and the final estimate was taken as the average of the two interpolated values. This approach preserved spatial consistency by incorporating basin-specific seasonal concentration dynamics across years.

(iv) **RSC – Relative scaling calculation before monitoring start:** If the time point predated the beginning of pharmaceutical monitoring and no concentration measurements were available, emissions were estimated indirectly using a relative scaling calculation. Specifically, the mean seasonal relative contribution of each station to total pharmaceutical emissions from centralized wastewater treatment plants (WWTPs) and decentralized wastewater treatment systems (DWTSs) in Saxony was determined based on monitoring-period data. This contribution factor was then scaled by the total gross emissions in years prior to pharmaceutical-specific monitoring to estimate trans-boundary emissions. This approach assumes comparable pharmaceutical consumption patterns in upstream trans-boundary basins and Saxony.

The procedure and methodology are summarized in the schematic diagram shown in Figure S3.

1.2 Text S2: Global Sensitivity Analysis (GSA)

Method

We conducted the Global Sensitivity Analysis (GSA) using the distribution-based PAWN method to characterize the uncertainty of eight (unknown) model factors affecting model simulations.[2,3] The goal of the PAWN method is to measure the difference between the cumulative distribution function (CDF) of output y from unconstrained input x_i (unconditional CDF, denotes as $F_y(y)$) and the CDFs of output y from constrained input $x_{i,k}$ (conditional CDF, denotes as $F_{y|x_{i,k}}\ (y|x_{i,k})$). In our case, y reflects the simulations for a given pharmaceutical concentration with a given set of x_i, where x_i represents the i-th input (or modeling) factor. A total number of N unconstrained x_i values are sampled from the entire range of the $i-th$ input factor. Table S3 provides details on initial ranges of input factors and their selection based on previous literature. Within GSA, the N unconstrained x_i samples are further split into n equiprobable intervals and consist of the constrained $x_{i,k}\,(k = 1, 2, 3, \ldots, n)$. The Kolmogorov-Smirnov (KS) statistic is applied to quantify the difference between unconditional CDF and conditional CDFs, which calculates the maximum vertical distance between $F_y(y)$ and $F_{y|x_{i,k}}\ (y|x_{i,k})$ (Eq. S1).

$$KS(x_i) = \max_y |F_y(y) - F_{y|x_{i,k}}(y|x_{i,k})| \tag{S1}$$

We defined the median KS values among n intervals as the PAWN sensitivity index ($S^i_{PAWN}(-)$) to assess the sensitivity of the $i-th$ input factor.

$$S^i_{PAWN}(-) = \underset{X_i}{Q_{50}} KS_{(x_i)} \tag{S2}$$

Here, we generated 10,000 input x_i sets (N = 10,000) using Latin Hypercube Sampling (LHS) method with uniform prior distribution (ranges shown in Table S3). Ten equiprobable

intervals ($n = 10$) were split for each $i - th$ input factor.

To examine the robustness of the sensitivity index, we bootstrapped the $F_y(y)$ for 1,000 times to compute the mean ($S^i_{PAWN\_}mn$) and the 95% confidence interval (CI) of the PAWN index. The sensitivity levels are decided by $S^i_{PAWN\_}mn$. Two thresholds were given at 0.05 and 0.1 to classify low, moderate, and high sensitivity level. The GSA was implemented by the Python version of SAFE toolbox.[4] We conducted the GSA for each of the five pharmaceuticals across all river reaches in Saxony to investigate the sensitivity of model parameterization, along with its spatial and temporal variations (warm and cool seasons).

Results

We investigated the uncertainty of our model by assessing the sensitivity of simulated pharmaceutical concentrations to input factors. Supporting CDF plots (Figures S14–S18) at four different nodes show that the dominant input factor causing model uncertainty varied across river nodes and pharmaceuticals. For example, conditional and unconditional CDFs of $p4 - f_{w\_c}$ (centralized wastewater treatment plant (namely WWTP) removal efficiency) varied greatly at WWTP nodes (Figure S14c, S14f), while $p8 - f_{pho}$ (photolysis rate) showed a similar trend. However, at MST (Figure S14b, S14e) and Linestring nodes (Figure S14a, S14d), $p5 - f_{w\_d}$ (decentralized wastewater treatment plant (namely DWTS) removal efficiency) contributed more to uncertainty than $p4 - f_{w\_c}$. Further, the PAWN index quantified sensitivity to input factors shows 1.4% of river nodes (n = 123) with zero sensitivity, as these nodes were upstream of WWTPs or DWTSs connections, with no emissions. Boxplots (Figures S19-S21) illustrate PAWN sensitivity indices and 95% bootstrap confidence intervals at three exemplary nodes. $p4 - f_{w\_c}$ had the highest sensitivity at WWTP nodes, while $p5 - f_{w\_d}$ dominated at other node types. These findings confirm that the dominant input factor changes across nodes, supporting the conclusions from the CDF analysis.

Figure S22 shows the percentage of rivers classified under different sensitivity levels for each input factor during the warm and cool seasons. Results indicate that pharmaceutical concentrations were relatively insensitive to human excretion rate ($p1 - f_{exc}$), human compliance rate ($p2 - f_{hcp}$), improper disposal rate ($p3 - f_{ipd}$), and biodegradation ($p6 - f_{bio}$). Removal efficiencies in WWTPs ($p4 - f_{w\_c}$) and DWTSs ($p5 - f_{w\_d}$) were the dominant anthropogenic factors influencing simulations for all five pharmaceuticals. Sensitivity to environmental factors varied. For example, metformin concentrations showed high sensitivity to hydrolysis ($p7 - f_{hyd}$) in 49% (cool) and 40% (warm) of rivers, while other pharmaceuticals were highly sensitive to photolysis ($p8 - f_{pho}$) in 58–89% of rivers. Sensitivity to $p5 - f_{w\_d}$ exceeded that of $p4 - f_{w\_c}$, as DWTSs (n = 117,000) are more numerous and spatially distributed than WWTPs (n = 700).[5] The modeling assumption that DWTS emissions are distributed across all model nodes, further emphasizing the importance of acquiring accurate DWTS information to enhance model accuracy.

Seasonally, the mean difference in the percentages of river length under high sensitivity ($S_{PAWN} > 0.1$) for the same factor between cool and warm seasons was $1.1\% \pm 1.9\%$, $3.6\% \pm 8.1\%$, $6.9\% \pm 20.5\%$, $0.7\% \pm 3.4\%$, and $-0.5\% \pm 1.8\%$ for carbamazepine, ciprofloxacin, gabapentin, metformin, and sulfamethoxazole, respectively (Figure S22). This indicates that most rivers showed similar sensitivity across seasons. However, gabapentin's sensitivity to hydrolysis ($p7 - f_{hyd}$) was high in 59% of rivers during cool seasons but fell to 0.9% in the warm seasons. Similarly, ciprofloxacin's sensitivity to compliance rate ($p3 - f_{hcp}$) decreased in 81% of rivers, shifting from moderate to low levels. These results highlight seasonal variability in sensitivity depending on pharmaceuticals and modeling factors.

Spatially, sensitivity also varied across the study region. Figures S23-S30 show the spatial distribution of PAWN indices for eight factors. Spatially, anthropogenic factors like $p1 - f_{exc}$, $p2 - f_{hcp}$, $p3 - f_{ipd}$, and $p5 - f_{w\_d}$ showed declining sensitivity from upstream to downstream, consistent with a dilution effect with increasing discharge. However, $p4 -$

f_{w_c} (WWTP removal efficiency) retained moderate to high sensitivity ($S_{PAWN} > 0.05$) downstream, likely due to continuous WWTP discharges. Another potential explanation for the high sensitivity along rivers is the long-distance impact of WWTPs on water quality.[6] Environmental factors $p7 - f_{hyd}$ and $p8 - f_{pho}$ followed similar spatial trends as $p4 - f_{w\_c}$, increasing sensitivity downstream, while $p6 - f_{bio}$ showed no spatial pattern. Overall, the findings of the sensitivity analysis allowed us to pinpoint critical factors (e.g., $p4 - f_{w\_c}$ or f_{pho} for photolysis) influencing simulations of pharmaceutical concentrations, with sensitivity varying seasonally and spatially.

1.3 Text S3: Concentrations Response to Hydrological and Climatic Changes

Sensitivity Analysis

To investigate the increase in algal risk in the warm season, a targeted sensitivity analysis was conducted to disentangle the relative effects of hydrological and climatic/temperature conditions on pharmaceutical concentrations in rivers. River discharge (Q) was used as a proxy for hydrological variation, while biodegradation and photolysis rates were treated as direct proxies for climatic influences. In contrast, hydrolysis, which being less directly affected by climatic variation, was held the same during model simulations to isolate the effects of the other two in-stream decay pathways. River discharge and decay rates were systematically varied across seven different levels: −99%, −66%, −33%, 0%, +33%, +66%, and +99%, resulting in 49 model combinations. For each combination, pharmaceutical concentrations across all Saxon rivers were simulated over the study period, resulting in a total of 20,580 runs. The resulting simulations for each simulations were then summarized as the respective flow-weighted mean concentrations ($fwmc$). The relative changes (%) in pharmaceutical $fwmc$ were calculated across all model combinations to assess the effect of hydrological and climatic conditions on pharmaceutical concentration, using the baseline model simulations as a reference.

The results (Figures S31–S35) indicate that, in our study, hydrological conditions have a stronger impact on pharmaceutical concentrations and associated ecotoxicological risks than climatic factors. This conclusion is supported by model combinations where only one factor (either degradation rate or river discharge) was varied while the other was remained constant. For example, as shown in Figure S31, in the long-term annual case, when climatic conditions were fixed to constant (0% ΔT) and hydrological conditions varied from –99% to

+99% (ΔQ), the resulting changes in $fwmc$ ranged from –48% to +7731% (middle vertical line). In contrast, when hydrological conditions were fixed (0% ΔQ) and climatic conditions varied across the same range (ΔT), the $fwmc$ changes were much smaller, ranging from –15% to +16% (middle horizontal line). These patterns were consistent across both warm and cool seasons (Figure S31) and were similarly observed for the other four pharmaceuticals examined (Figures S32–S35).

Damköhler Number Analysis

To further quantify the interplay between transport processes (hydraulic residence time) and reactive processes (decay rate) in each river reach, we employed a Damköhler number (Da) analysis.[7] The Damköhler number can be presented as:

$$Da = \frac{\text{Reactive time}}{\text{Transport time}} \tag{S3}$$

A Da value greater than 1 indicates that the time available for reactions is longer than the time it takes water to traverse the reach, meaning reaction processes dominate the fate of pharmaceuticals (reaction-dominated conditions). Conversely, a Da less than 1 suggests that water moves through the reach faster than reactions can significantly reduce pharmaceutical concentrations, meaning transport processes dominate (transport-dominated conditions). This analysis examines how hydrologic transport and in-stream decay jointly shape the concentration behavior in each river reach (see Eq. 8 in the main text). In that equation, the term $\sum_{m=1}^{3} k_{m,d_{i-j}}$ represents the effective decay rate (the inverse of the reaction timescale), whereas the ratio $\frac{d_{i-j}}{v_{d_{i-j}}}$ gives the hydrologic travel time between nodes i and j.

Our analysis reveals that, under most simulated conditions, Da remains below 1 (Figure S36), highlighting the dominance of hydrological processes in controlling pharmaceutical concentrations. This finding supports the results of the sensitivity analysis on the pharma-

ceutical concentration response to changes in hydrological and decay rate variations.

1.4 Text S4: Uncertainty of Risk Level Composition

To quantify the uncertainty associated with the composition of risk levels in the Saxon river network, we employed a non-parametric bootstrapping approach. The analysis focused on four fractional components, representing the percentage contributions of different ecotoxicological risk levels (from single and mixture pharmaceutical exposures) to three representative aquatic taxa: algae, Daphnia, and fish. For each group and season, 10,000 bootstrap resamples were drawn with replacement from the original time series data (n = 42), representing six months of data per year from 2008 to 2014. For each sample, the mean values of the four risk fractions were calculated. The distribution of these bootstrap means was then used to retrieved group-wise 95% confidence intervals, which is reported as the uncertainty of the mean values in Figure 5A of the main text. The results of risk level composition uncertainty are presented in Figure S13 and Table S7. This nonparametric approach employed here provides a reliable estimate of central tendency and uncertainty without assuming any specific underlying distribution.

1.5 Text S5: Risk Assessment by OPBT-Score

Method

To assess the comprehensive environmental risk posed by pharmaceuticals in aquatic ecosystems, we employed a scoring method based on four OPBT criteria, which are Occurrence (O), Persistence (P), Bioaccumulation (B), and Toxicity (T), using proxy indicators.[8,9] Each indicator was assigned a separate score from 1 (lowest concern) to 5 (highest concern), according to threshold values defined in Table S10.

- **Occurrence (O)** was evaluated using the simulated flow-weighted mean concentration in each model node (Concentration, in $ng\,L^{-1}$).
- **Persistence (P)** was based on the removal efficiency (R, in %) in centralized wastewater treatment plants (WWTPs), with values parameterized from the model.
- **Bioaccumulation (B)** was estimated using the logarithm of the octanol–water partition coefficient ($log\,K_{ow}$), serving as a proxy for the potential of pharmaceuticals to (bio)accumulate in aquatic organisms. The underlying information were retrieved from PubChem[10].
- **Toxicity (T)** was assessed using the predicted no-effect concentration (PNEC, in $ng\,L^{-1}$) for ecosystem species, based on the values listed in Table S4.

Each proxy was classified into one of five score categories according to predefined thresholds. If no value was available for a given proxy, the highest score (5) was assigned by default to reflect precautionary prioritization. The total OPBT score for each pharmaceutical was calculated as the sum of the individual scores across the four criteria. Additionally, individual indicator scores were aggregated to assess the potential cumulative risk from pharmaceutical mixtures.

Results

The spatial distribution of OPBT scores showed relatively a low variability across Saxon rivers for carbamazepine, ciprofloxacin, gabapentin, and sulfamethoxazole (Figures S37–S38). This homogeneous pattern was found in both season and all three species for the same pharmaceuitcal. In contrast, metformin exhibited higher OPBT scores in the Zwickauer Mulde River and downstream of the Elbe River during the cool season. A similar pattern was also found for the pharmaceutical mixture, which showed higher scores in both seasons in these river basins.

Seasonal differences were also observed in the total OPBT scores. For ciprofloxacin, scores were significantly higher in the cool season compared to the warm season across all three species (p-*value* < 0.01), which aligns with the ecotoxicological risk assessment assessed by the risk quotient (RQ) method. However, in contrast to RQ method results, total OPBT scores for the mixture were significantly higher in the cool season than in the warm season for all three species (p-*value* < 0.01). These findings highlight the importance of conducting risk assessments from multiple perspectives, or at least clearly stating the conceptual framework from which risk is being evaluated. Since the OPBT scoring criteria for bioaccumulation (based on $\log K_{\mathrm{ow}}$) and toxicity (based on $PNEC$) remain constant between seasons, observed seasonal difference in total OPBT scores are mainly driven by changes in occurrence (concentrations) and persistence (removal efficiency in WWTPs), and should therefore be interpreted accordingly.

Figure S39 presents the median stacked scores for occurrence, persistence, bioaccumulation, and toxicity for each pharmaceutical and the mixture across species and seasons. The results indicate that persistence was the main contributor to total OPBT risk for both antibiotics (ciprofloxacin and sulfamethoxazole) and for gabapentin. In the case of carbamazepine, risk was more evenly distributed among persistence, bioaccumulation, and toxicity. For met-

formin, high scores were mainly resulted from the high concentrations in the environment. These findings provide insights into potential strategies for reducing pharmaceutical risks to aquatic ecosystems, such as minimizing emissions at the source or improving removal efficiencies during wastewater treatment.

1.6 Text S6: DWTS Emission Sensitivity

Method

To assess the potential spatial bias from uniformly distributed decentralized wastewater treatment system (DWTS) emissions within each community, we implemented two more scenarios to evaluate the robustness of the spatial allocation method.

(1) No DWTS emission scenario

In this scenario, emissions from DWTSs were entirely omitted from the model. River node only received gross emission from centralized wastewater treatment plant (WWTPs) and emission from the trans-boundary basins.

(2) Discharge augmented (Q-weighted) DWTS allocation scenario

In this scenario, emissions from DWTSs were preferentially allocated to river nodes with higher discharge, under the assumption that such locations have a greater likelihood of receiving treated wastewater. For each node $i \in C$, where C is the set of nodes within a community, we calculated the mean river discharge Q_i over the period 2008–2014. The community-level mean discharge $\bar{Q}_C$ was computed as the average of all Q_i values within that community. A relative discharge factor for each node was then defined as:

$$k_i = \frac{Q_i}{\bar{Q}_C} \tag{S4}$$

This factor was normalized across all nodes in the community to yield a weighting coefficient:

$$w_i = \frac{k_i}{\sum_{i \in C} k_i} \tag{S5}$$

These weights were applied to the gross pharmaceutical emissions from DWTSs, previously estimated at the community level, to proportionally allocate emissions to indi-

vidual model nodes.

Results

In the scenario of no DWTS emission (left panel in Figure S40), compared to uniformly distributed gross emission from DWTS at each community, the flow-weighted mean concentration decreased in all river reaches. The median relative changes across Saxon reaches were −75% ($p05-p95$: −100% to −2%), −88% (−100% to −4%), −76% (−100% to −2%), −86% (−100% to −2%), −74% (−100% to −2%) for carbamazepine, ciprofloxacin, gabapentin, metformin, and sulfamethoxazole, respectively.

In the scenario of discharge augmented (Q-weighted) DWTS emission (right panel in Figure S40), pharmaceutical concentrations remained unchanged at $0\,ng\,L^{-1}$ in 0.9% of Saxon river reaches. In 0.5% of reaches, concentrations increased from $0\,\mathrm{ng\,L^{-1}}$ to various levels depending on the pharmaceutical. The median absolute increases in these reaches were $0.16\,ng\,L^{-1}$ for carbamazepine, $0.19\,ng\,L^{-1}$ for ciprofloxacin, $2.51\,ng\,L^{-1}$ for gabapentin, $5.54\,ng\,L^{-1}$ for metformin, and $0.14\,ng\,L^{-1}$ for sulfamethoxazole. For the remaining river network, the median relative changes in concentrations compared to the uniform distribution scenario were moderate: −16% ($p05 - p95$: −99% to 9%) for carbamazepine, −16% (−99% to 26%) for ciprofloxacin, −15% (−99% to 12%) for gabapentin, −19% (−99% to 13%) for metformin, and −13% (−99% to 13%) for sulfamethoxazole. River segments with higher concentrations relative to the uniform distribution scenario were predominantly located along main rivers, which are characterized by higher river discharges.

In summary, the high concentration difference in the no DWTS scenario compared to uniformly distributed DWTS emission highlights the importance of including DWTSs in the modeling framework, as their omission leads to a clear underestimation of pharmaceutical emissions in all river reaches. The Q-weighted DWTS scenario further demonstrates that,

while the spatial redistribution of DWTS emissions based on river discharge affects local concentration patterns, these differences do not significantly alter the overall regional trends. In our study, the uniform distribution of DWTS emissions may lead to underestimation of concentrations in main rivers with higher discharge and overestimation in smaller tributaries. Therefore, we emphasize the need for more accurate spatial data on DWTS locations to reduce uncertainties in modeling the fate and transport of pharmaceuticals in rivers.

1.7 Text S7: Assessment of Model Sensitivity to WWTP Performance Variability

Method

To assess the potential impact of different removal efficiencies in centralized wastewater treatment plant (WWTP) on model outcomes, we evaluated how changes in assumed treatment performance influence pharmaceutical concentrations in Saxon rivers during 2008–2014.

In the absence of detailed information on WWTP treatment technologies, WWTPs were classified into five categories (K1 to K5) based on their design capacity, expressed in population equivalents (PE), following criteria adapted from Yang et al.[11], DEFoJ[12]: K1 for <1,000 PE, K2 for 1,000–5,000 PE, K3 for 5,000–10,000 PE, K4 for 10,000–100,000 PE, and K5 for ≥100,000 PE.

Two scenarios were considered:

- **Scenario 1:** Based on the currently parameterized removal efficiencies in WWTPs, adjustments of –10%, –5%, 0%, +5%, and +10% were applied to each pharmaceutical for WWTPs K1, K2, K3, K4, and K5, respectively, across all seasons.
- **Scenario 2:** Based on the currently parameterized removal efficiencies in WWTPs, adjustments of –20%, –10%, 0%, +10%, and +20% were applied to each pharmaceutical for WWTPs K1, K2, K3, K4, and K5, respectively, across all seasons.

Results

Results of this sensitivity analysis are presented as spatial distribution of simulated concentrations (Figure S41) and their relative differences (Figure S42) compared to the baseline scenario (as presented in the manuscript). In the **baseline scenario**, median

flow-weighted mean concentration ($fwmc$) estimated over the Saxon rivers varied between 27 $ng\,L^{-1}$ ($p05-p95$: $0.5-180\,ng\,L^{-1}$), 7.7 $ng\,L^{-1}$ ($0.2-78.9\,ng\,L^{-1}$), 218.3 $ng\,L^{-1}$ ($5.7-1918.4\,ng\,L^{-1}$), 516.5 $ng\,L^{-1}$ ($13.6-3285.8\,ng\,L^{-1}$), and 10.3 $ng\,L^{-1}$ ($0.3-101.4\,ng\,L^{-1}$) for carbamazepine, ciprofloxacin, gabapentin, metformin, and sulfamethoxazole, respectively. Whereas these values under the **Scenario 1** (Figure S41A) ranged from 26.8 $ng\,L^{-1}$ ($p05-p95$: $0.5-181.7\,ng\,L^{-1}$), 7.7 $ng\,L^{-1}$ ($p05-p95$: $0.2-79.1\,ng\,L^{-1}$), 218.3 $ng\,L^{-1}$ ($p05-p95$: $5.7-1925.6\,ng\,L^{-1}$), 546.4 $ng\,L^{-1}$ ($p05-p95$: $13.6-3420.8\,ng\,L^{-1}$), and 10.3 $ng\,L^{-1}$ ($p05-p95$: $0.3-101.7\,ng\,L^{-1}$) for respective pharmaceuticals. Under the **Scenario 2** (Figure S41B), $fwmc$ estimated from 26.9 $ng\,L^{-1}$ ($p05-p95$: $0.5-183.7\,ng\,L^{-1}$), 7.7 $ng\,L^{-1}$ ($p05-p95$: $0.2-79.5\,ng\,L^{-1}$), 218.7 $ng\,L^{-1}$ ($p05-p95$: $5.7-1936.4\,ng\,L^{-1}$), 576.4 $ng\,L^{-1}$ ($p05-p95$: $13.7-3622.1\,ng\,L^{-1}$), and 10.4 $ng\,L^{-1}$ ($p05-p95$: $0.3-101.4\,ng\,L^{-1}$) for carbamazepine, ciprofloxacin, gabapentin, metformin, and sulfamethoxazole, respectively. Further, the spatial variability of $fwmc$ across Saxon rivers for each pharmaceuticals in both scenarios follows that of the baseline scenario (see Figure 4 in the main text). In terms of differences, metformin exhibited a relatively higher sensitivity to removal efficiencies in WWTPs. This is more evident in the analysis of relative changes (%) as shown in Figure S42.

Under the Scenario 1, the relative changes in $fwmc$ ($p05$–$p95$) ranged from −0.7% to 1.8%, −1.2% to 1.6%, −2.8% to 2.8%, −3.5% to 22.5%, and −2.1% to 2.1% for carbamazepine, ciprofloxacin, gabapentin, metformin, and sulfamethoxazole, respectively. Under the Scenario 2, the corresponding changes were −1.4% to 3.5%, −1.6% to 3.5%, −5.6% to 5.5%, −6.8% to 45.0%, and −4.2% to 4.2%, respectively. Metformin showed the greatest variation, with an increase of up to +45.0% in the Scenario 2, whereas changes for the other pharmaceuticals were relatively small. These findings highlight the importance of incorporating technology-specific removal efficiencies in wastewater treatment plants, particularly for pharmaceuticals with high removal rates such as metformin, when tracking their fate in receiving rivers.

2 Supporting Tables

Table S1: Description of data set and data source.

Data type	Spatial resolution	Time period	Temporal resolution	Data source
Consumption from the primary care sector	Weekly	2008 – 2014	Postal code area	[13]
Consumption from the hospital sector	Monthly	2008 – 2014	– [1]	[13]
Hospital bed number	–	2014	–	[13,14]
Hospital location	–	2023	–	[14]
Community inhabitants share of each WWTP [2]	–	2014	Community	[13]
Inhabitant-specific wastewater disposal practices	–	2021	Community	[1,5]
Demographic data [3]	Yearly	2008 – 2014	Community	[15]
Saxon topographic data	–	–	20-meter grid	[16]
Global elevation data	–	–	7.5-arc-second	[17]
Pharmaceutical concentration	Monthly	2008 – 2018 [4]	–	[1]
River discharge	Monthly	1980 – 2018	1.2 km	[18,19]
WWTPs locations	–	–	–	[1]
European river networks	–	–	–	[20]
Aquatic life benchmarks	–	–	–	[21]

[1] unitless; [2] Centralized wastewater treatment plant;
[3] includes population number and age composition; [4] Data the entire period was used for parameterization of environmental (in-stream decay) factors, including for training (calibration) and testing (validation) purposes. For other investigations, i.e, parameterization of anthropogenic factors, model evaluation assessment, and calculation of emission from upstream trans-boundary basins, data from 2008 to 2014 was used.

Table S2: Correspondence between LfULG water quality station IDs (ID_LfULG) and the trans-boundary station IDs defined in this study (ID_UBB) and observations count during 2008–2014.

ID_LfULG [1]	ID_UBB [2]	Count	ID_LfULG	ID_UBB	Count
OBF49310	S1	44	OBF04700	S14	34
OBF54260	S2	30	OBF05401	S15	29
OBF40700	S3	4	OBF05300	S16	4
Node_24148 [3]	S4	0	OBF03400	S17	3
OBF49500	S5	187	OBF02950	S18	31
OBF50550	S6	32	OBF16800	S19	63
OBF53250	S7	6	Node_23872 [3]	S20	0
OBF50930	S8	34	OBF18500	S21	49
OBF40621	S9	0	OBF36400	S22	40
OBF02855	S10	4	OBF37700	S23	0
OBF00200	S11	270	Node_23787 [3]	S24	0
OBF03800	S12	16	OBF09800	S25	26
OBF03505	S13	3	OBF31200	S26	4

[1] Identifier of water quality station provided by the Saxon State Agency for Environment, Agriculture and Geology (LfULG) for calculating emission from upstream trans-boundary basins; [2] Identifier of the trans-boundary stations in this study; [3] The identifiers used refer to model nodes rather than official LfULG station IDs. As there are no water quality monitoring stations on the boarder of this trans-boundary river, the trans-boundary stations were defined based on the river network in Saxony[1] and in the EU.[20] Due to the absence of observed concentration data, concentrations were assumed to be half the limit of quantification for the purpose of calculating emissions from the upstream trans-boundary basins.

Table S3: Description and ranges of the model factors. The first five factors are classified as anthropogenic factors, while the remaining three are categorized as environmental factors.

Factor	Description	Pharmaceutical	Calibrated value Cool / Warm season	Unit	Lower bound	Upper bound	References
		Carbamazepine	0.18 / 0.16		0.15	0.2	
f_{exc}	Human excretion rate	Ciprofloxacin	0.76 / 0.69	(−)	0.40	0.80	22–26
		Gabapentin	1.00/ 0.99		0.99	1.00	
		Metformin	0.91 / 0.88		0.86	0.95	
		Sulfamethoxazole	0.21/ 0.21		0.19	0.25	
		Carbamazepine	0.80 / 0.92		0.70	1.00	
f_{hcp}	Human compliance rate	Ciprofloxacin	0.97 / 0.77	(−)	0.72	0.99	27–30
		Gabapentin	0.99/ 0.98		0.98	1.00	
		Metformin	0.92 / 0.92		0.91	1.00	
		Sulfamethoxazole	0.97/ 0.83		0.83	0.99	
		Carbamazepine	0.20 / 0.19		0.16	0.28	
f_{ipd}	Improper disposal rate	Ciprofloxacin	0.23 / 0.25	(−)	0.16	0.28	27–30
		Gabapentin	0.21/ 0.22		0.16	0.28	
		Metformin	0.19 / 0.22		0.16	0.28	
		Sulfamethoxazole	0.23/ 0.24		0.16	0.28	
		Carbamazepine	0.27 / 0.26		-0.50	0.50	
f_{w_c}	Removal efficiency in WWTPs [1]	Ciprofloxacin	0.03 / 0.68	(−)	-1.00	1.00	31–41
		Gabapentin	-1.35/ -1.28		-2.00	0.50	
		Metformin	0.75 / 0.90		0.72	1.00	
		Sulfamethoxazole	-0.47/ -1.09		-2.00	0.90	
		Carbamazepine	0.23 / -0.13		-0.63	0.38	
f_{w_d}	Removal efficiency in DWTSs [2]	Ciprofloxacin	-0.27 / -2.08	(−)	-3.00	0.75	31–41
		Gabapentin	-1.95/ -1.47		-2.50	0.38	
		Metformin	0.64 / 0.55		0.49	0.75	
		Sulfamethoxazole	-0.72/ -1.29		-1.88	0.68	
		Carbamazepine	$1.74/2.88 \times 10^{-8}$		1.14×10^{-9}	1.21×10^{-7}	
f_{bio}	Biodegradation rate	Ciprofloxacin	$3.77/4.70 \times 10^{-8}$	s^{-1}	2.49×10^{-9}	4.15×10^{-7}	42,43
		Gabapentin	$2.17/2.81 \times 10^{-8}$		3.61×10^{-9}	4.01×10^{-7}	
		Metformin	$3.82/3.79 \times 10^{-8}$		3.61×10^{-9}	4.41×10^{-7}	
		Sulfamethoxazole	$2.89/3.34 \times 10^{-8}$		1.34×10^{-7}	1.38×10^{-5}	
		Carbamazepine	$6.25/3.56 \times 10^{-10}$		8.35×10^{-11}	1.67×10^{-8}	
f_{hyd}	Hydrolysis rate	Ciprofloxacin	$1.32/1.07 \times 10^{-9}$	s^{-1}	1.39×10^{-10}	3.39×10^{-8}	42,43
		Gabapentin	$4.32/3.66 \times 10^{-6}$		3.33×10^{-7}	7.32×10^{-5}	
		Metformin	$9.30/7.88 \times 10^{-6}$		1.65×10^{-7}	1.73×10^{-5}	
		Sulfamethoxazole	$1.34/1.13 \times 10^{-9}$		7.43×10^{-10}	9.90×10^{-8}	
		Carbamazepine	$6.16/7.82 \times 10^{-6}$		4.27×10^{-7}	4.43×10^{-5}	
f_{pho}	Photolysis rate	Ciprofloxacin	$7.69/8.66 \times 10^{-5}$	s^{-1}	1.81×10^{-5}	3.02×10^{-3}	42,43
		Gabapentin	$1.87/2.15 \times 10^{-5}$		1.00×10^{-6}	1.03×10^{-4}	
		Metformin	$4.42/4.70 \times 10^{-7}$		5.49×10^{-9}	5.83×10^{-7}	
		Sulfamethoxazole	$3.61/3.94 \times 10^{-5}$		1.67×10^{-6}	1.72×10^{-4}	

[1] Centralized wastewater treatment plants; [2] Decentralized wastewater treatment systems.

Table S4: Toxic effect concentration and PNEC concentration for selected pharmaceuticals.

Parameter	Species	Endpoint	Effect concentration[21] (ng L^{-1})	AF [1]	PNEC [2] (ng L^{-1})
Carbamazepine	Algae	EC_{50}	2.83×10^6	1000	2833
Ciprofloxacin	Algae	EC_{50}	7.77×10^6	1000	7769
Gabapentin	Algae	EC_{50}	1.69×10^7	1000	16850
Metformin	Algae	EC_{50}	6.23×10^7	1000	62320
Sulfamethoxazole	Algae	EC_{50}	1.00×10^7	1000	10020
Carbamazepine	Daphnia	EC_{50}	7.53×10^6	1000	7530
Ciprofloxacin	Daphnia	EC_{50}	4.47×10^6	1000	4467
Gabapentin	Daphnia	EC_{50}	2.47×10^7	1000	24740
Metformin	Daphnia	EC_{50}	7.43×10^7	1000	74290
Sulfamethoxazole	Daphnia	EC_{50}	3.74×10^6	1000	3743
Carbamazepine	Fish	LC_{50}	3.26×10^6	1000	3263
Ciprofloxacin	Fish	LC_{50}	5.75×10^6	1000	5752
Gabapentin	Fish	LC_{50}	4.26×10^7	1000	42570
Metformin	Fish	LC_{50}	3.73×10^7	1000	37320
Sulfamethoxazole	Fish	LC_{50}	6.25×10^6	1000	6253

[1] Assessment factor; [2] Predicted no-effect concentration.

Table S5: Model performance metrics among river basins.

River basin	N [1]	ρ [2]	*p-value*	nRMSE [3]	Well-fitted range (%) [4]
Elbe	154	0.79	< 0.01	0.08	93.5
Freiberger Mulde	92	0.55	< 0.01	0.31	84.8
Lausitzer Neiße	15	0.82	< 0.01	0.16	73.3
Schwarze Elster	34	0.79	< 0.01	0.07	76.5
Spree	41	0.77	< 0.01	0.10	75.6
Vereinigte Mulde	45	0.70	< 0.01	0.47	84.4
Weiße Elster	119	0.77	< 0.01	0.80	83.2
Zwickauer Mulde	85	0.76	< 0.01	0.20	89.4

[1] N: number of measurements; [2] ρ: Spearman's rank correlation coefficient; [3] nRMSE: normalized root-mean-square error; [4] Well-fitted range: the percentage of simulations within 1 order of magnitude of observations.

Table S6: Monthly percentage of rivers exposed to varying ecological risk categories for three trophic level species.

Parameter	Risk level [1]	Algae (%)				Daphnia (%)				Fish (%)			
		Min [2]	Max [3]	Median	Mean	Min	Max	Median	Mean	Min	Max	Median	Mean
Carbamazepine	NS	41.0	60.5	47.5	50.0	67.5	83.2	72.5	74.7	44.4	63.7	50.9	53.5
Carbamazepine	Low	36.3	52.2	46.1	44.9	16.2	31.0	26.3	24.2	33.8	50.1	43.9	42.4
Carbamazepine	Moderate	2.1	7.4	5.5	4.8	0.6	1.5	1.1	1.0	1.6	6.1	4.4	3.8
Carbamazepine	High	0.2	0.3	0.3	0.3	0.1	0.2	0.1	0.1	0.2	0.3	0.3	0.3
Ciprofloxacin	NS	88.4	93.6	91.1	91.1	80.4	87.5	83.8	84.0	84.5	90.6	87.4	87.6
Ciprofloxacin	Low	6.1	11.1	8.6	8.6	11.9	18.9	15.5	15.3	8.9	15.0	12.2	11.9
Ciprofloxacin	Moderate	0.2	0.5	0.3	0.3	0.4	1.1	0.6	0.6	0.3	0.8	0.4	0.4
Ciprofloxacin	High	0.0	0.0	0.0	0.0	0.0	0.1	0.0	0.0	0.0	0.1	0.0	0.0
Gabapentin	NS	32.6	47.3	41.2	40.9	40.8	56.3	50.6	49.9	53.4	69.3	63.8	63.3
Gabapentin	Low	45.0	50.8	49.7	48.8	39.1	48.0	44.0	43.7	28.7	40.8	33.5	33.6
Gabapentin	Moderate	6.9	16.0	9.7	9.8	4.0	10.7	5.9	6.1	1.8	5.6	2.6	2.9
Gabapentin	High	0.4	0.8	0.4	0.5	0.3	0.5	0.3	0.3	0.2	0.3	0.2	0.2
Metformin	NS	43.4	59.7	53.0	52.3	47.7	63.9	58.1	57.2	32.1	46.8	38.6	38.8
Metformin	Low	37.8	50.1	44.0	44.2	34.1	47.3	39.6	40.1	47.7	57.3	54.1	53.5
Metformin	Moderate	2.1	6.2	3.1	3.3	1.6	4.7	2.3	2.5	4.8	12.8	7.0	7.3
Metformin	High	0.1	0.3	0.2	0.2	0.1	0.3	0.2	0.2	0.2	0.6	0.3	0.3
Sulfamethoxazole	NS	88.5	95.2	91.9	92.1	72.0	83.5	77.6	77.9	81.8	90.9	86.2	86.5
Sulfamethoxazole	Low	4.6	10.9	7.7	7.6	15.7	25.9	21.0	20.7	8.7	17.2	13.1	12.8
Sulfamethoxazole	Moderate	0.3	0.5	0.4	0.4	0.7	2.0	1.3	1.2	0.4	0.9	0.6	0.6
Sulfamethoxazole	High	0.0	0.0	0.0	0.0	0.1	0.2	0.1	0.1	0.0	0.1	0.1	0.1
Mixture	NS	17.4	27.2	20.9	21.3	20.7	31.9	25.0	25.2	17.8	28.9	21.9	22.2
Mixture	Low	48.6	57.2	52.6	52.9	52.8	59.0	55.3	55.8	50.4	58.3	54.9	54.6
Mixture	Moderate	18.9	31.5	25.7	24.6	13.5	25.0	19.2	18.3	16.6	29.1	23.4	22.3
Mixture	High	0.7	2.1	1.0	1.1	0.5	1.3	0.7	0.8	0.6	1.6	0.8	0.9

[1] Nonsignificant (NS), low, moderate, and high risk are classified with the range of $RQ < 0.01$, $0.01 \leq RQ < 0.1$, $0.1 \leq RQ < 1$ and $RQ \geq 1$, respectively (RQ: risk quotient); [2] Min: minimum; [3] Max: maximum.

Table S7: Mean and 95% confidence intervals (CI) of species-specific risk fractions across seasons and pharmaceuticals.

Parameter	Risk level [1]	Species	Cool (%) Mean (95% CI)	Warm (%) Mean (95% CI)
Carbamazepine	NS	Algae	54.9 (51.5–58.7)	45.0 (41.3–49.1)
Carbamazepine	NS	Daphnia	79.3 (76.1–82.4)	70.0 (66.3–73.8)
Carbamazepine	NS	Fish	58.5 (55.0–62.4)	48.5 (44.6–52.7)
Carbamazepine	Low	Algae	41.4 (38.4–44.3)	48.5 (45.3–51.1)
Carbamazepine	Low	Daphnia	19.8 (16.9–22.9)	28.6 (25.1–32.0)
Carbamazepine	Low	Fish	38.5 (35.3–41.6)	46.2 (42.9–49.2)
Carbamazepine	Moderate	Algae	3.4 (2.6–4.2)	6.2 (5.0–7.5)
Carbamazepine	Moderate	Daphnia	0.8 (0.6–0.9)	1.2 (1.0–1.5)
Carbamazepine	Moderate	Fish	2.7 (2.1–3.3)	5.0 (4.0–6.1)
Carbamazepine	High	Algae	0.3 (0.2–0.3)	0.3 (0.3–0.4)
Carbamazepine	High	Daphnia	0.1 (0.1–0.1)	0.1 (0.1–0.2)
Carbamazepine	High	Fish	0.2 (0.2–0.3)	0.3 (0.2–0.3)
Ciprofloxacin	NS	Algae	91.9 (90.5–93.2)	90.4 (89.0–91.7)
Ciprofloxacin	NS	Daphnia	85.4 (83.3–87.4)	82.7 (80.7–84.7)
Ciprofloxacin	NS	Fish	88.7 (87.0–90.4)	86.5 (84.8–88.2)
Ciprofloxacin	Low	Algae	7.7 (6.4–9.0)	9.4 (8.1–10.8)
Ciprofloxacin	Low	Daphnia	13.8 (11.9–15.6)	16.8 (14.8–18.7)
Ciprofloxacin	Low	Fish	10.7 (9.1–12.3)	13.1 (11.5–14.8)
Ciprofloxacin	Moderate	Algae	0.4 (0.3–0.4)	0.2 (0.2–0.2)
Ciprofloxacin	Moderate	Daphnia	0.8 (0.6–0.9)	0.5 (0.4–0.6)

Continued on next page

Parameter	Risk level [1]	Species	Cool (%) Mean (95% CI)	Warm (%) Mean (95% CI)
Ciprofloxacin	Moderate	Fish	0.5 (0.4–0.7)	0.3 (0.3–0.4)
Ciprofloxacin	High	Algae	0.0 (0.0–0.0)	0.0 (0.0–0.0)
Ciprofloxacin	High	Daphnia	0.1 (0.1–0.1)	0.0 (0.0–0.0)
Ciprofloxacin	High	Fish	0.0 (0.0–0.1)	0.0 (0.0–0.0)
Gabapentin	NS	Algae	41.6 (37.8–45.5)	40.3 (37.3–43.8)
Gabapentin	NS	Daphnia	50.6 (46.5–54.8)	49.2 (46.0–52.8)
Gabapentin	NS	Fish	64.2 (59.9–68.4)	62.4 (59.2–65.7)
Gabapentin	Low	Algae	48.4 (46.2–50.4)	49.1 (46.9–51.0)
Gabapentin	Low	Daphnia	43.1 (40.2–45.9)	44.2 (41.5–46.6)
Gabapentin	Low	Fish	32.8 (29.3–36.3)	34.5 (31.6–37.2)
Gabapentin	Moderate	Algae	9.6 (7.4–11.9)	10.1 (8.7–11.6)
Gabapentin	Moderate	Daphnia	6.0 (4.5–7.7)	6.3 (5.3–7.3)
Gabapentin	Moderate	Fish	2.9 (2.0–3.8)	2.9 (2.4–3.4)
Gabapentin	High	Algae	0.5 (0.4–0.6)	0.5 (0.4–0.5)
Gabapentin	High	Daphnia	0.3 (0.3–0.4)	0.3 (0.3–0.3)
Gabapentin	High	Fish	0.2 (0.2–0.2)	0.2 (0.2–0.2)
Metformin	NS	Algae	53.2 (49.2–57.4)	51.3 (47.5–55.4)
Metformin	NS	Daphnia	57.9 (53.8–62.1)	56.4 (52.7–60.3)
Metformin	NS	Fish	40.4 (36.8–44.1)	37.2 (33.7–41.3)
Metformin	Low	Algae	43.2 (39.6–46.5)	45.1 (41.6–48.3)
Metformin	Low	Daphnia	39.3 (35.6–42.9)	41.0 (37.4–44.2)
Metformin	Low	Fish	52.1 (49.5–54.4)	54.9 (51.8–57.5)

Continued on next page

Parameter	Risk level [1]	Species	Cool (%) Mean (95% CI)	Warm (%) Mean (95% CI)
Metformin	Moderate	Algae	3.3 (2.5–4.3)	3.4 (2.8–4.0)
Metformin	Moderate	Daphnia	2.5 (1.9–3.2)	2.5 (2.0–2.9)
Metformin	Moderate	Fish	7.1 (5.4–9.1)	7.5 (6.3–8.7)
Metformin	High	Algae	0.3 (0.2–0.3)	0.2 (0.1–0.2)
Metformin	High	Daphnia	0.2 (0.2–0.2)	0.1 (0.1–0.2)
Metformin	High	Fish	0.4 (0.3–0.4)	0.3 (0.2–0.3)
Sulfamethoxazole	NS	Algae	93.6 (92.4–94.6)	90.5 (88.9–92.0)
Sulfamethoxazole	NS	Daphnia	80.7 (78.1–83.2)	75.1 (72.1–78.0)
Sulfamethoxazole	NS	Fish	88.7 (86.8–90.4)	84.3 (82.1–86.4)
Sulfamethoxazole	Low	Algae	6.1 (5.0–7.2)	9.0 (7.6–10.5)
Sulfamethoxazole	Low	Daphnia	18.2 (15.9–20.6)	23.2 (20.5–25.8)
Sulfamethoxazole	Low	Fish	10.8 (9.2–12.5)	14.9 (12.8–16.9)
Sulfamethoxazole	Moderate	Algae	0.3 (0.3–0.4)	0.4 (0.4–0.5)
Sulfamethoxazole	Moderate	Daphnia	1.0 (0.8–1.2)	1.5 (1.2–1.8)
Sulfamethoxazole	Moderate	Fish	0.5 (0.4–0.6)	0.7 (0.6–0.9)
Sulfamethoxazole	High	Algae	0.0 (0.0–0.0)	0.0 (0.0–0.0)
Sulfamethoxazole	High	Daphnia	0.1 (0.1–0.1)	0.1 (0.1–0.2)
Sulfamethoxazole	High	Fish	0.1 (0.0–0.1)	0.1 (0.1–0.1)
Mixture	NS	Algae	22.9 (20.7–25.3)	19.7 (17.6–22.2)
Mixture	NS	Daphnia	27.0 (24.3–29.8)	23.4 (21.0–26.3)
Mixture	NS	Fish	24.3 (22.0–26.8)	20.2 (18.0–22.8)
Mixture	Low	Algae	53.5 (51.7–55.2)	52.4 (50.8–53.9)

Continued on next page

Parameter	Risk level [1]	Species	Cool (%) Mean (95% CI)	Warm (%) Mean (95% CI)
Mixture	Low	Daphnia	55.6 (54.0–57.1)	56.0 (54.5–57.3)
Mixture	Low	Fish	54.6 (53.0–56.1)	54.6 (52.9–56.2)
Mixture	Moderate	Algae	22.4 (19.1–25.9)	26.7 (23.7–29.6)
Mixture	Moderate	Daphnia	16.7 (13.8–19.7)	19.8 (17.3–22.2)
Mixture	Moderate	Fish	20.2 (17.0–23.5)	24.3 (21.3–27.2)
Mixture	High	Algae	1.1 (0.9–1.4)	1.1 (1.0–1.3)

[1] Nonsignificant (NS), low, moderate, and high risk are classified with the range of $RQ < 0.01$, $0.01 \leq RQ < 0.1$, $0.1 \leq RQ < 1$, and $RQ \geq 1$, respectively (RQ: risk quotient).

Table S8: Frequency of river segments exposed to at-risk ecological toxicity for three trophic level species.

Parameter	Risk level [1]	Algae (%)				Daphnia (%)				Fish (%)			
		Min [2]	Max [3]	Median	Mean	Min	Max	Median	Mean	Min	Max	Median	Mean
Carbamazepine	NS	0	100.0	44.0	49.6	0	100.0	92.9	74.3	0	100.0	53.6	53.2
Carbamazepine	Low	0	100.0	48.8	45.2	0	100.0	7.1	24.6	0	100.0	41.7	42.7
Carbamazepine	Moderate	0	97.6	0.0	4.9	0	92.9	0.0	1.0	0	97.6	0.0	3.9
Carbamazepine	High	0	96.4	0.0	0.3	0	61.9	0.0	0.1	0	95.2	0.0	0.2
Ciprofloxacin	NS	0	100.0	100.0	91.1	0	100.0	100.0	84.1	0	100.0	100.0	87.6
Ciprofloxacin	Low	0	100.0	0.0	8.6	0	98.8	0.0	15.2	0	100.0	0.0	11.9
Ciprofloxacin	Moderate	0	79.8	0.0	0.3	0	96.4	0.0	0.6	0	84.5	0.0	0.4
Ciprofloxacin	High	0	40.5	0.0	0.0	0	67.9	0.0	0.0	0	56.0	0.0	0.0
Gabapentin	NS	0	100.0	23.8	40.8	0	100.0	45.2	49.8	0	100.0	79.8	63.1
Gabapentin	Low	0	100.0	54.8	48.8	0	100.0	44.0	43.7	0	100.0	17.9	33.8
Gabapentin	Moderate	0	96.4	0.0	10.0	0	96.4	0.0	6.2	0	96.4	0.0	2.9
Gabapentin	High	0	100.0	0.0	0.4	0	100.0	0.0	0.3	0	98.8	0.0	0.2
Metformin	NS	0	100.0	48.8	52.1	0	100.0	61.9	57.0	0	100.0	20.2	38.7
Metformin	Low	0	98.8	46.4	44.4	0	98.8	35.7	40.4	0	100.0	64.3	53.6
Metformin	Moderate	0	97.6	0.0	3.4	0	97.6	0.0	2.5	0	95.2	0.0	7.4
Metformin	High	0	86.9	0.0	0.2	0	83.3	0.0	0.2	0	96.4	0.0	0.3
Sulfamethoxazole	NS	0	100.0	100.0	91.9	0	100.0	97.6	77.7	0	100.0	100.0	86.3
Sulfamethoxazole	Low	0	98.8	0.0	7.7	0	98.8	2.4	20.9	0	97.6	0.0	13.0
Sulfamethoxazole	Moderate	0	97.6	0.0	0.4	0	95.2	0.0	1.3	0	98.8	0.0	0.6
Sulfamethoxazole	High	0	57.1	0.0	0.0	0	94.0	0.0	0.1	0	75.0	0.0	0.1
Mixture	NS	0	100.0	1.2	21.3	0	100.0	3.6	25.2	0	100.0	2.4	22.2
Mixture	Low	0	100.0	59.5	52.8	0	100.0	65.5	55.7	0	100.0	61.9	54.4
Mixture	Moderate	0	100.0	3.6	24.8	0	100.0	1.2	18.4	0	98.8	2.4	22.5
Mixture	High	0	100.0	0.0	1.1	0	100.0	0.0	0.7	0	100.0	0.0	0.9

[1] Nonsignificant (NS), low, moderate, and high risk are classified with the range of $RQ < 0.01$, $0.01 \leq RQ < 0.1$, $0.1 \leq RQ < 1$ and $RQ \geq 1$, respectively (RQ: risk quotient); [2] Min: minimum; [3] Max: maximum.

Table S9: Comparison of pharmaceutical fate models with spatial, temporal, pathway characteristics, and emission inputs.

Model	Spatial scale (model output)	Spatial resolution (model output)	Temporal scale (model output)	Temporal resolution (model output)	Spatio-temporal resolution (model input)	Data input[1]	Environmental pathways[2]	Anthropogenic pathways[3]
HydroFATE [44]	Globe	500 m	2015	Yearly	Country; yearly	E_{w_c}, E_{w_d}	f_{bio}	f_{exc}, f_{w_c}, f_{w_d}
hydroROUT [45]	St. Lawrence River (Canada)	500 m	2006	Yearly	Country; yearly	E_{w_c}, E_{ubb}	f_{bio}	f_{exc}, f_{w_c}
ePiE [46]	Ouse River (UK)	1 km	2016	Yearly	Country; yearly	E_{w_c}	f_{bio}, f_{pho}, f_{hyd}, f_{sed}, f_{vol}	f_{exc}, f_{w_c}
Model [47]	Yangtze River (China)	500 m	2015	Yearly	Country; yearly	E_{w_c}, E_{w_d}	f_{pho}, f_{bio}, f_{hyd}	f_{exc}, f_{w_c}
GREAT-ER [48,49]	Ruhr River (Germany)	2 km	2009	Yearly	Country; yearly	E_{w_c}	f_{bio}, f_{pho}, f_{hyd}, f_{sed}, f_{vol}	f_{w_c}
STREAM-EU [50]	Swedish rivers	—	2011	Yearly	Country; yearly	E_{w_c}	f_{per}, f_{dis}, f_{vol}, f_{ion}	f_{exc}, f_{w_c}
This study	Saxony (Germany)	1 km	2008–2014	Monthly	Postal area; weekly to monthly	E_{w_c}, E_{w_d}, E_{ubb}	f_{pho}, f_{bio}, f_{hyd}	f_{exc}, f_{hcp}, f_{ipd}, f_{w_c}, f_{w_d}

[1] Data input: E_{w_c}: Emission from WWTP, E_{w_d}: Emission from DWTS, E_{ubb}: Emission from upstream trans-boundary basins.

[2] Environmental pathways: f_{bio}: biodegradation, f_{dis}: dissolution, f_{hyd}: hydrolysis, f_{ion}: ionization, f_{pho}: photolysis, f_{per}: precipitation, f_{sed}: sedimentation, f_{vol}: volatilization.

[3] Anthropogenic pathways: f_{exc}: human excretion, f_{hcp}: human compliance, f_{ipd}: improper disposal, f_{w_c}: removal in centralized wastewater treatment plant (WWTP), f_{w_d}: removal in decentralized wastewater treatment system (DWTS).

Table S10: Scoring scheme for the OPBT criteria based on proxy indicators.

Score	**Occurrence (O)** Concentration c ($ng\,L^{-1}$)	**Persistence (P)** Removal in WWTP R (%)	**Bioaccumulation (B)** $\log K_{\mathrm{ow}}$ [1]	**Toxicity (T)** PNEC [2] ($ng\,L^{-1}$)
1	$c < 50$	$R > 80$	$\log K_{\mathrm{ow}} < 1$	$\mathrm{PNEC} > 1 \times 10^5$
2	$50 \leq c < 100$	$60 < R \leq 80$	$1 \leq \log K_{\mathrm{ow}} < 2$	$1 \times 10^4 < \mathrm{PNEC} \leq 1 \times 10^5$
3	$100 \leq c < 500$	$40 < R \leq 60$	$2 \leq \log K_{\mathrm{ow}} < 3$	$1 \times 10^3 < \mathrm{PNEC} \leq 1 \times 10^4$
4	$500 \leq c < 1000$	$20 < R \leq 40$	$3 \leq \log K_{\mathrm{ow}} < 4.5$	$1 \times 10^2 < \mathrm{PNEC} \leq 1 \times 10^3$
5	$c \geq 1000$ *or no value*	$R \leq 20$ *or no value*	$\log K_{\mathrm{ow}} \geq 4.5$ *or no value*	$\mathrm{PNEC} \leq 1 \times 10^2$ *or no value*

[1] Logarithm of the octanol–water partition coefficient; [2] Predicted no-effect concentration.

3 Supporting Figures

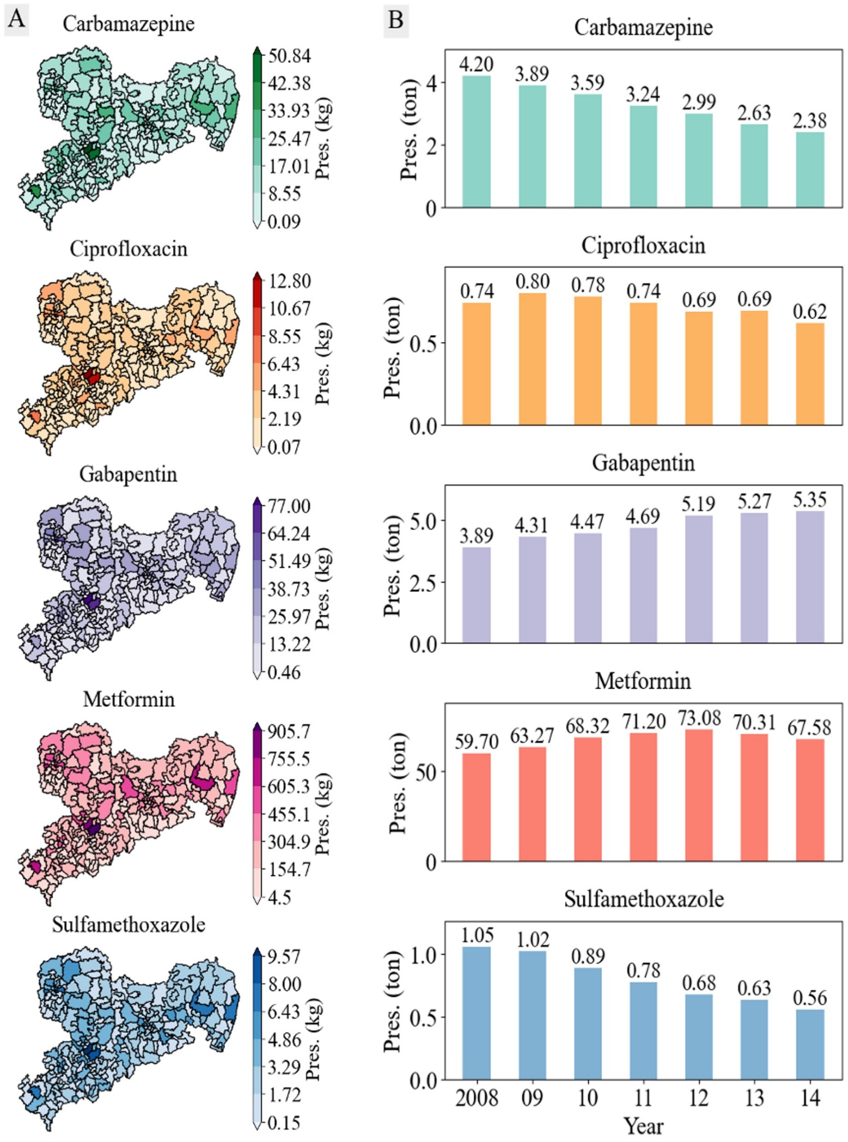

Figure S1: Spatial distribution (A) and temporal trend (B) of pharmaceutical consumption from the primary care sector in Saxony. Shown are the multi-year mean annual prescription (Pres.) during 2008 and 2014 at per postcode area.

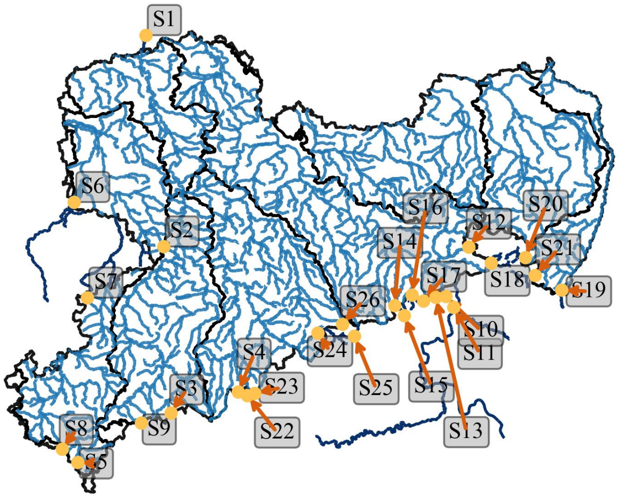

Figure S2: Geographical locations of trans-boundary stations. Station labels on the map are abbreviated for clarity; corresponding full water quality station identifiers from the Saxon State Agency for Environment, Agriculture and Geology (LfULG)[1] are listed in the Table S2.

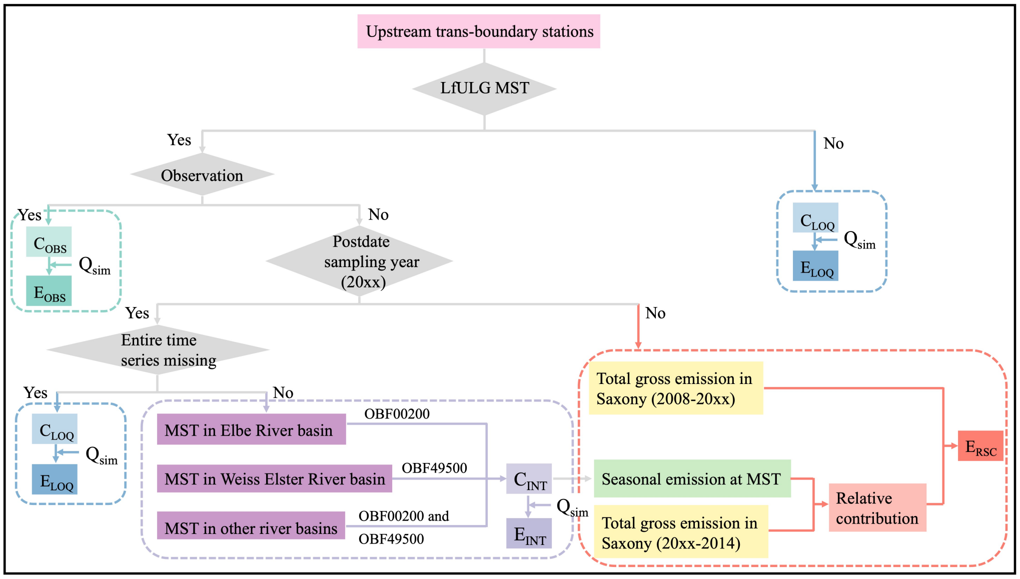

Figure S3: Flowchart for estimating pharmaceutical emissions from upstream trans-boundary basins. LfULG: the Saxon State Agency for Environment, Agriculture and Geology.[1] MST: water quality station. Q_{sim}: simulated river discharge from the mHM model.[18,19] The placeholder "20xx" denotes the starting year of pharmaceutical sampling by LfULG, specifically: 2008 for carbamazepine, 2010 for gabapentin and sulfamethoxazole, 2011 for ciprofloxacin, and 2012 for metformin. "OBS", "INT", "RSC", and "LOQ" indicates different approaches applied to calculate the emissions from the upstream trans-boundary basins, with further details provided in the Supporting Text 1.1.

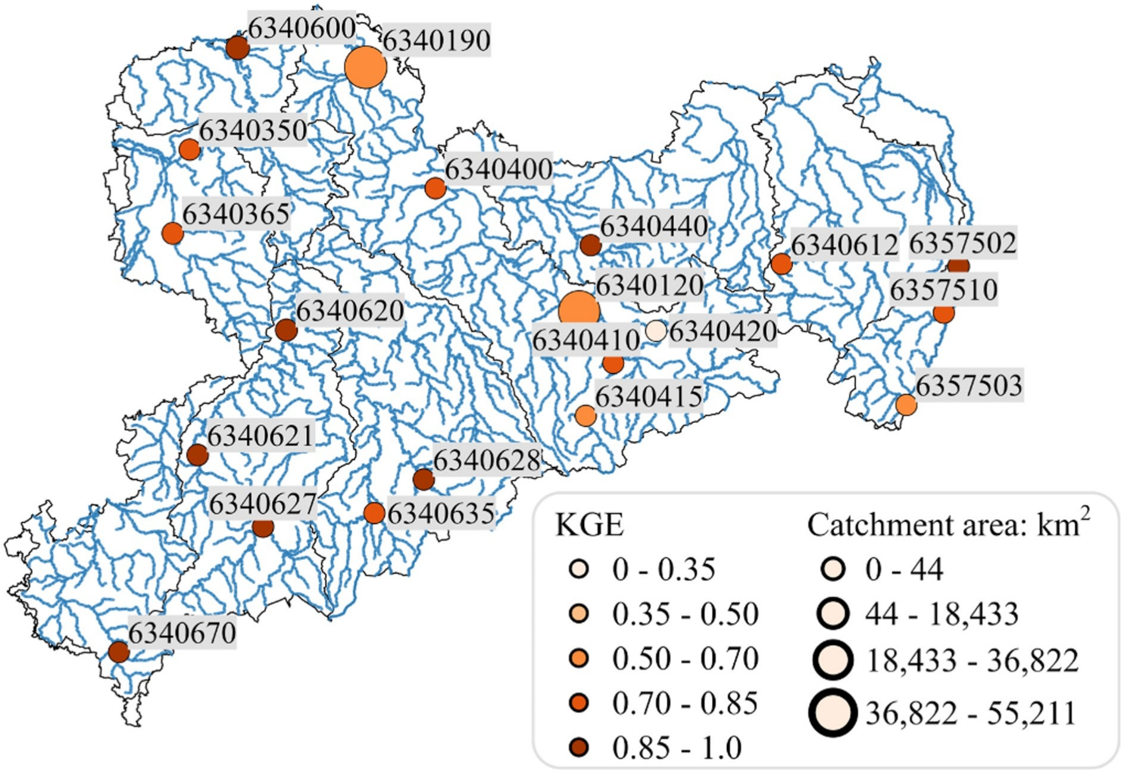

Figure S4: Spatial distribution of gauge stations for river discharge simulation. The size of each point corresponds to the basin area (km^2), while the color indicates simulation efficiency based on Kling-Gupta efficiency (KGE) values. The identifiers of the gauge stations are listed in the data set by Global Runoff Data Centre (GRDC, https://portal.grdc.bafg.de).

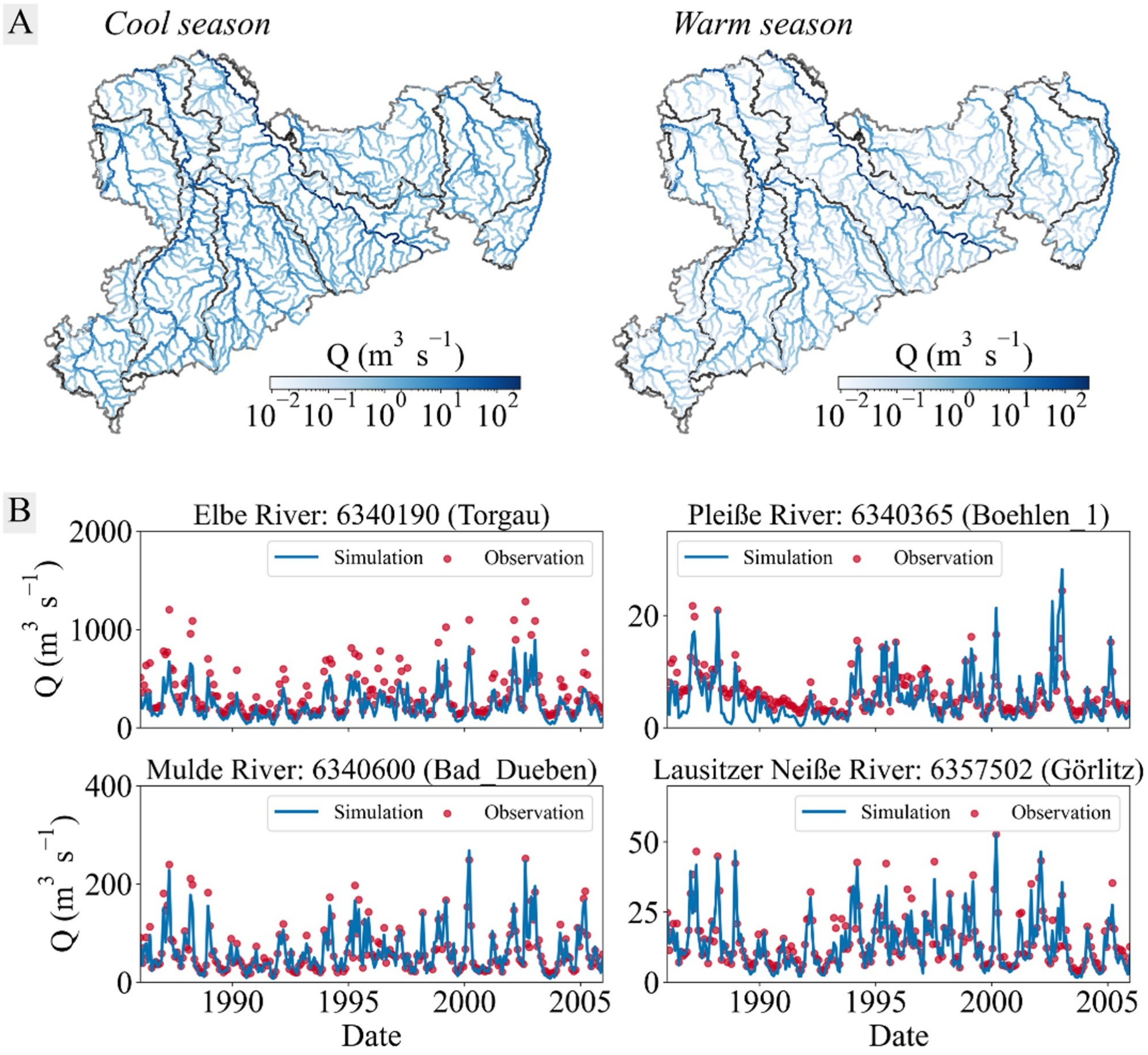

Figure S5: Long-term mean river discharges from the mesoscale hydrologic model (mHM) (A); Observed and simulated monthly river discharges at four stations located in different river basins (B). See Figure S4 for the respective identifiers.

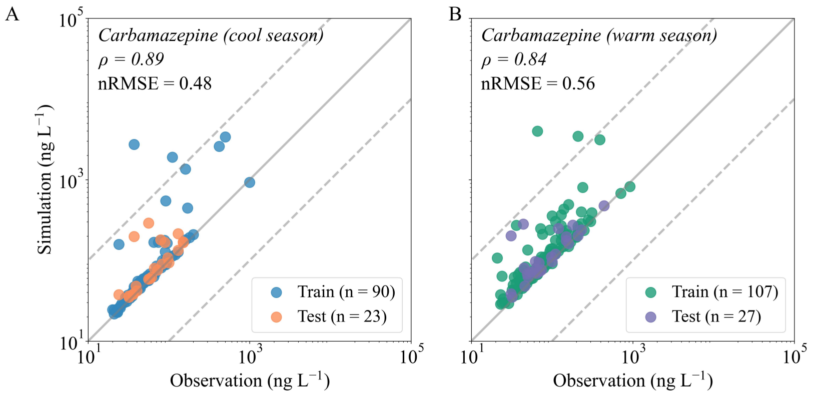

Figure S6: Evaluation of model environmental factors for carbamazepine using training and testing data sets in (A) cool and (B) warm season. Observation data are retrieved from the LfULG.[1] Training and testing data sets were obtained by randomly splitting the full data set into training (calibration; 80%) and testing (validation; 20%) subsets. The values shown on the plot represent the overall performance metrics across the entire data set, including the Spearman's rank correlation coefficient (ρ) and the normalized root-mean-square error (nRMSE). In the cool season, the training data set yielded $\rho = 0.91$ and nRMSE $= 0.54$, while the testing data set showed $\rho = 0.72$ and nRMSE $= 0.54$. In the warm season, the training data set achieved $\rho = 0.86$ and nRMSE $= 0.63$, while the testing data set resulted in $\rho = 0.62$ and nRMSE $= 0.16$.

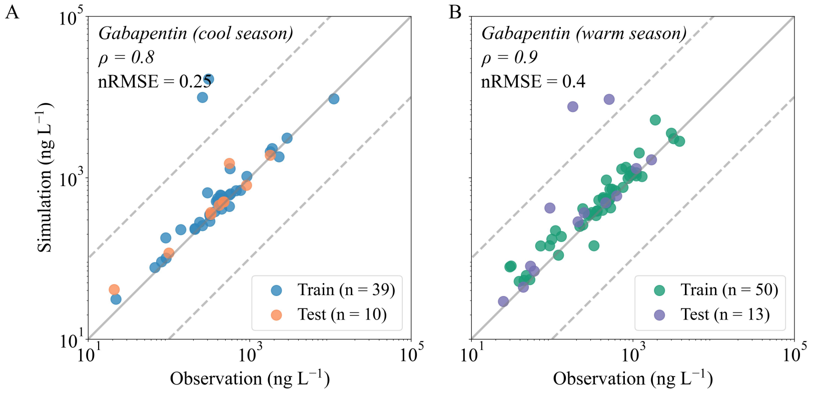

Figure S7: Evaluation of model environmental factors for gabapentin using training and testing data sets in (A) cool and (B) warm season. Observation data are retrieved from the LfULG.[1] Training and testing data sets were obtained by randomly splitting the full data set into training (calibration; 80%) and testing (validation; 20%) subsets. The values shown on the plot represent the overall performance metrics across the entire data set, including the Spearman's rank correlation coefficient (ρ) and the normalized root-mean-square error (nRMSE). In the cool season, the training data set yielded $\rho = 0.78$ and nRMSE $= 0.28$, while the testing data set showed $\rho = 0.98$ and nRMSE $= 0.17$. In the warm season, the training data set achieved $\rho = 0.96$ and nRMSE $= 0.14$, while the testing data set resulted in $\rho = 0.80$ and nRMSE $= 1.91$.

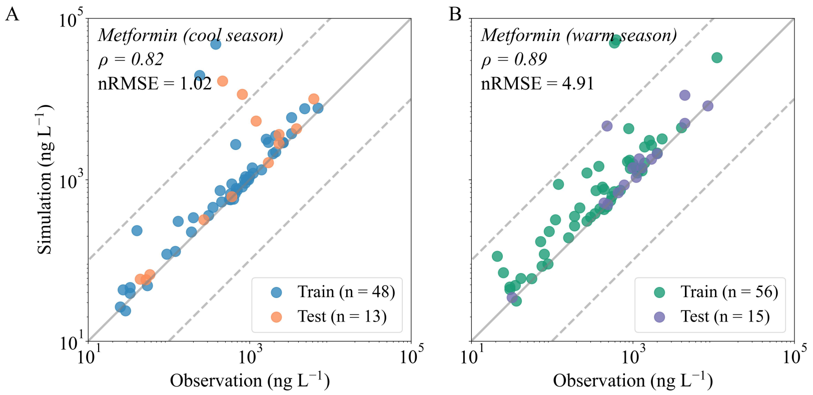

Figure S8: Evaluation of model environmental factors for metformin using training and testing data sets in (A) cool and (B) warm season. Observation data are retrieved from the LfULG.[1] Training and testing data sets were obtained by randomly splitting the full data set into training (calibration; 80%) and testing (validation; 20%) subsets. The values shown on the plot represent the overall performance metrics across the entire data set, including the Spearman's rank correlation coefficient (ρ) and the normalized root-mean-square error (nRMSE). In the cool season, the training data set yielded $\rho = 0.85$ and nRMSE = 1.07, while the testing data set showed $\rho = 0.62$ and nRMSE = 0.91. In the warm season, the training data set achieved $\rho = 0.90$ and nRMSE = 5.53, while the testing data set resulted in $\rho = 0.80$ and nRMSE = 0.24.

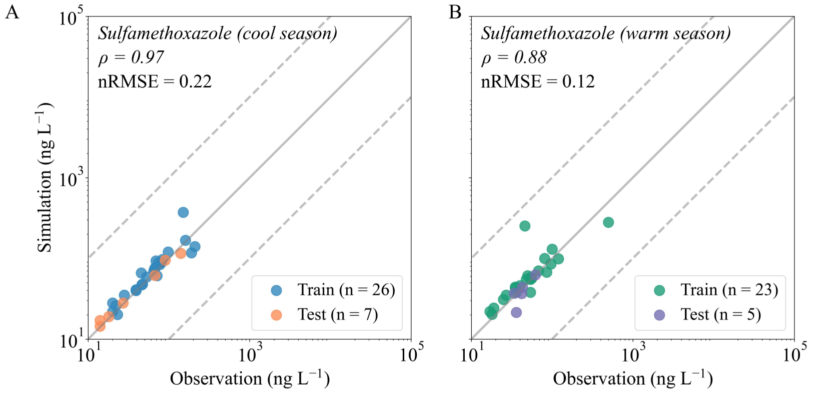

Figure S9: Evaluation of model environmental factors for sulfamethoxazole using training and testing data sets in (A) cool and (B) warm season. Observation data are retrieved from the LfULG.[1] Training and testing data sets were obtained by randomly splitting the full data set into training (calibration; 80%) and testing (validation; 20%) subsets. The values shown on the plot represent the overall performance metrics across the entire data set, including the Spearman's rank correlation coefficient (ρ) and the normalized root-mean-square error (nRMSE). In the cool season, the training data set yielded $\rho = 0.96$ and nRMSE = 0.26, while the testing data set showed $\rho = 0.99$ and nRMSE = 0.08. In the warm season, the training data set achieved $\rho = 0.87$ and nRMSE = 0.13, while the testing data set resulted in $\rho = 0.70$ and nRMSE = 0.25.

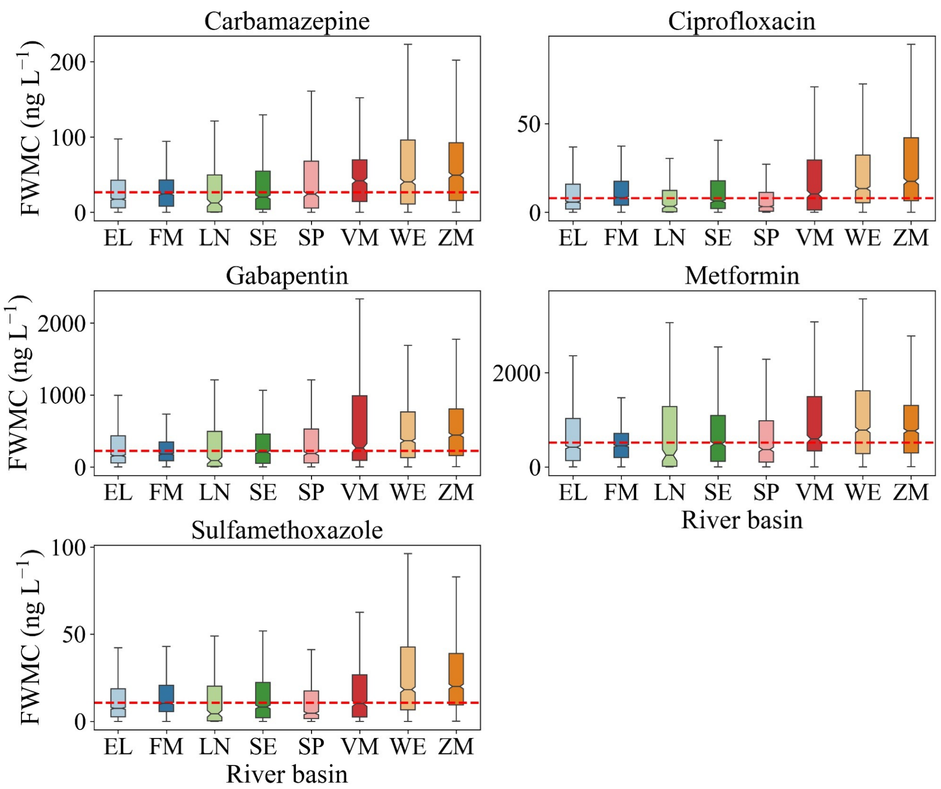

Figure S10: Simulation of flow-weighted mean pharmaceutical concentrations (FWMC) in the main river basin of Saxony. EL: Elbe; FM: Freiberger Mulde; LN: Lausitzer Neiße; SE: Schwarze Elster; SP: Spree; VM: Vereinigte Mulde; WE: Weiße Elster; ZM: Zwickauer Mulde. Outliers (defined as values beyond 1.5 times the interquartile range from the quartiles) were excluded from the plot.

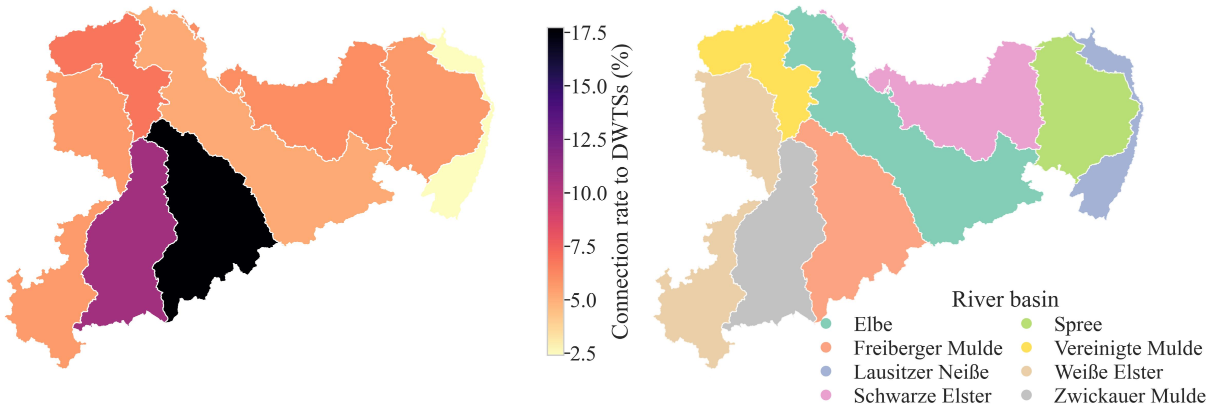

Figure S11: Decentralized wastewater treatment system (DWTS) connection rates across river basins. Values were derived by spatially aggregating community-level DWTS connection data to the river basin level using area-weighted averaging.

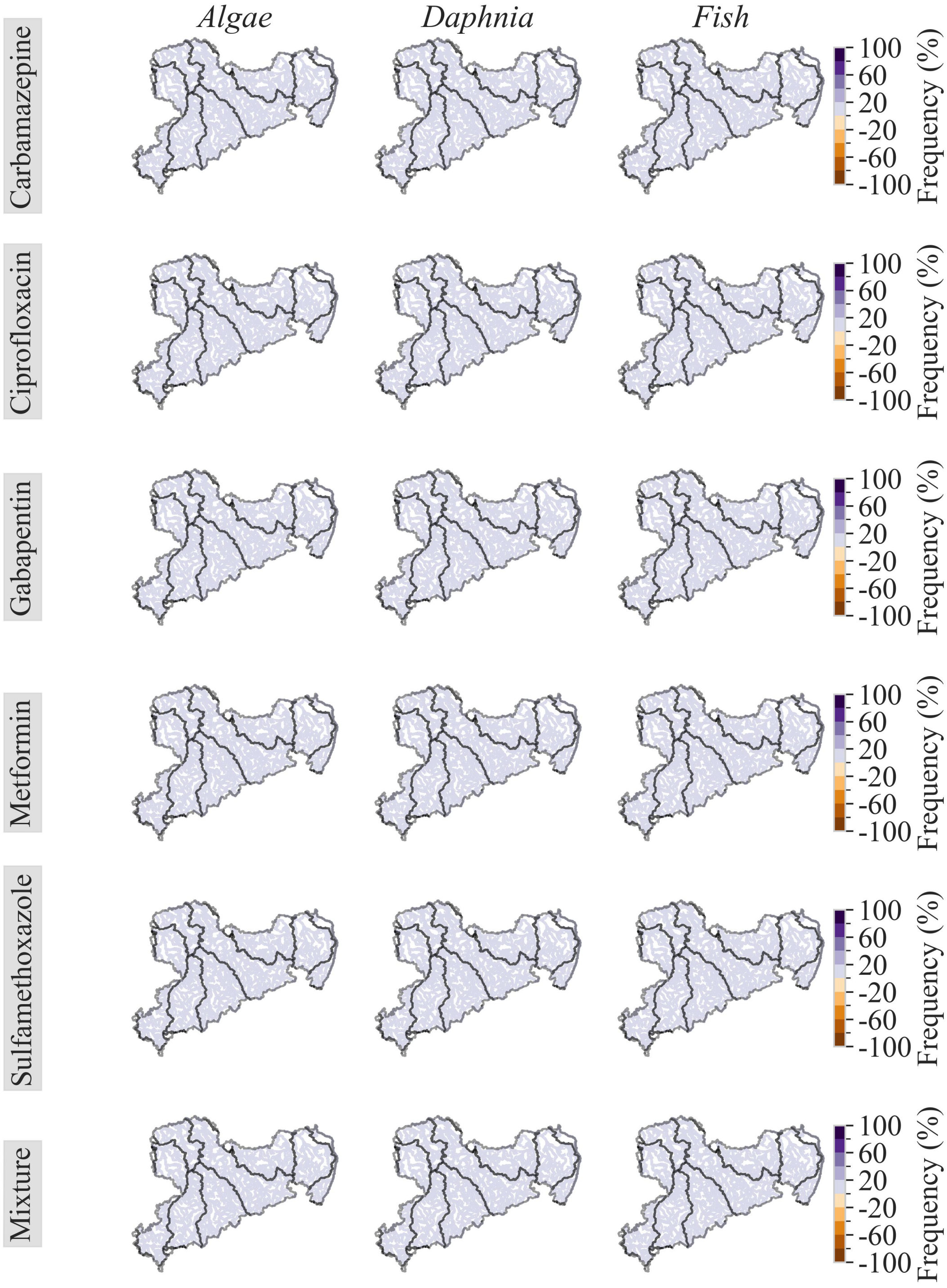

Figure S12: Difference in the frequency of at-risk ecological toxicity caused by single pharmaceuticals and their mixture, based on curated data versus the 5th percentile of experimental toxicity values.[21] The plot shows no difference in at-risk frequency between the two data sets, which applies to single pharmaceuticals and in mixtures.

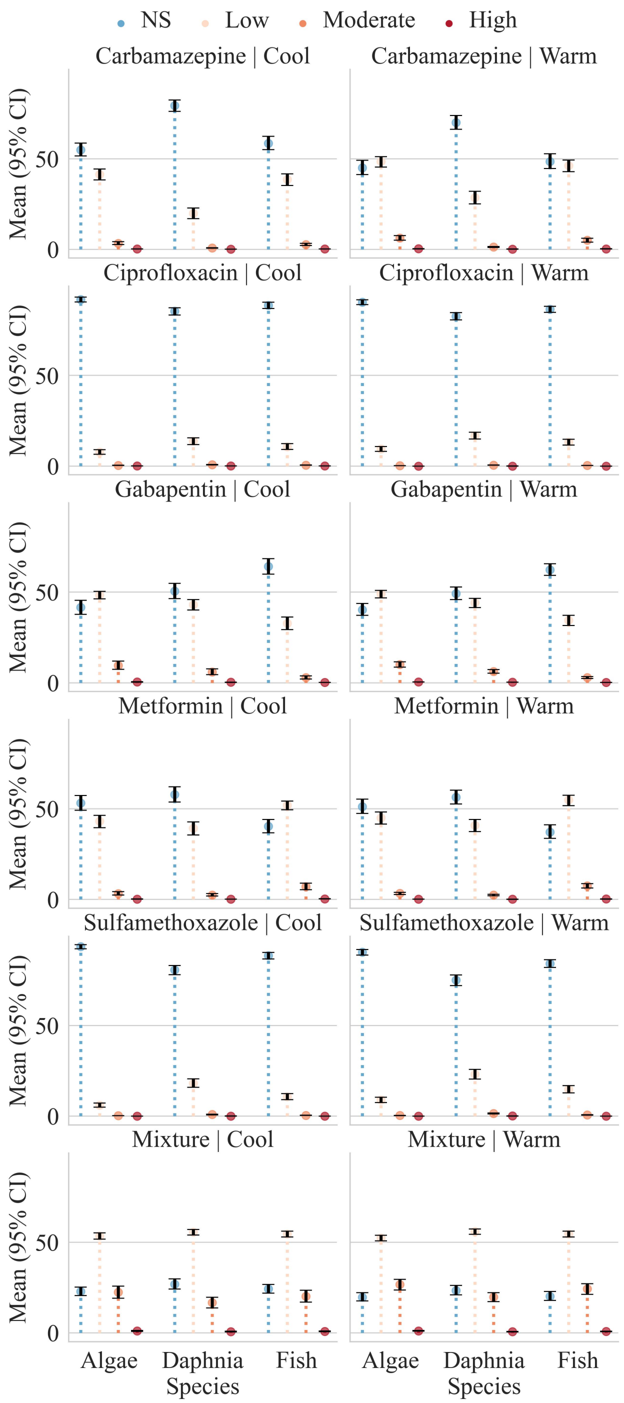

Figure S13: Mean and 95% confidence intervals (CI) of species-specific risk fractions across seasons (cool and warm) and pharmaceuticals (single and mixture). Points represent the mean contribution of each risk level to overall ecotoxicological risk, while vertical error bars indicate the 95% confidence intervals of the mean values based on 10,000 bootstrap samples. Risk levels are categorized as NS (nonsignificant), low, moderate, and high, corresponding to thresholds of 0.01, 0.1, and 1 times the predicted no-effect concentration (PNEC), respectively.

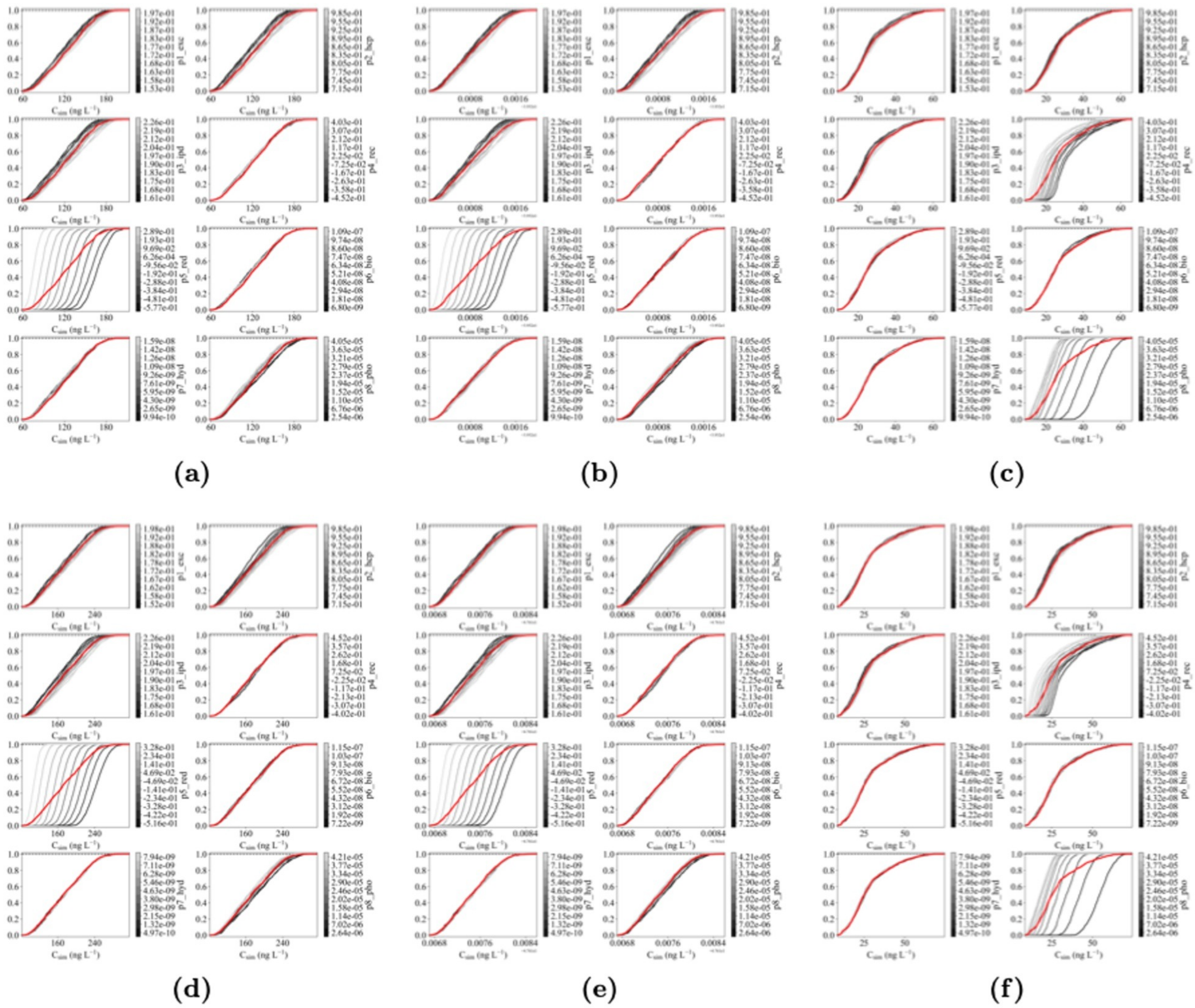

Figure S14: Cumulative distribution functions (CDFs) of the model factors for carbamazepine simulations at node $Linestring\_5000$**(a,d), node** MST_8584 **(b,e),node** $WWTP\_5746$ **(c,f) in the cool (a,b,c) and warm (d,e,f) seasons.**

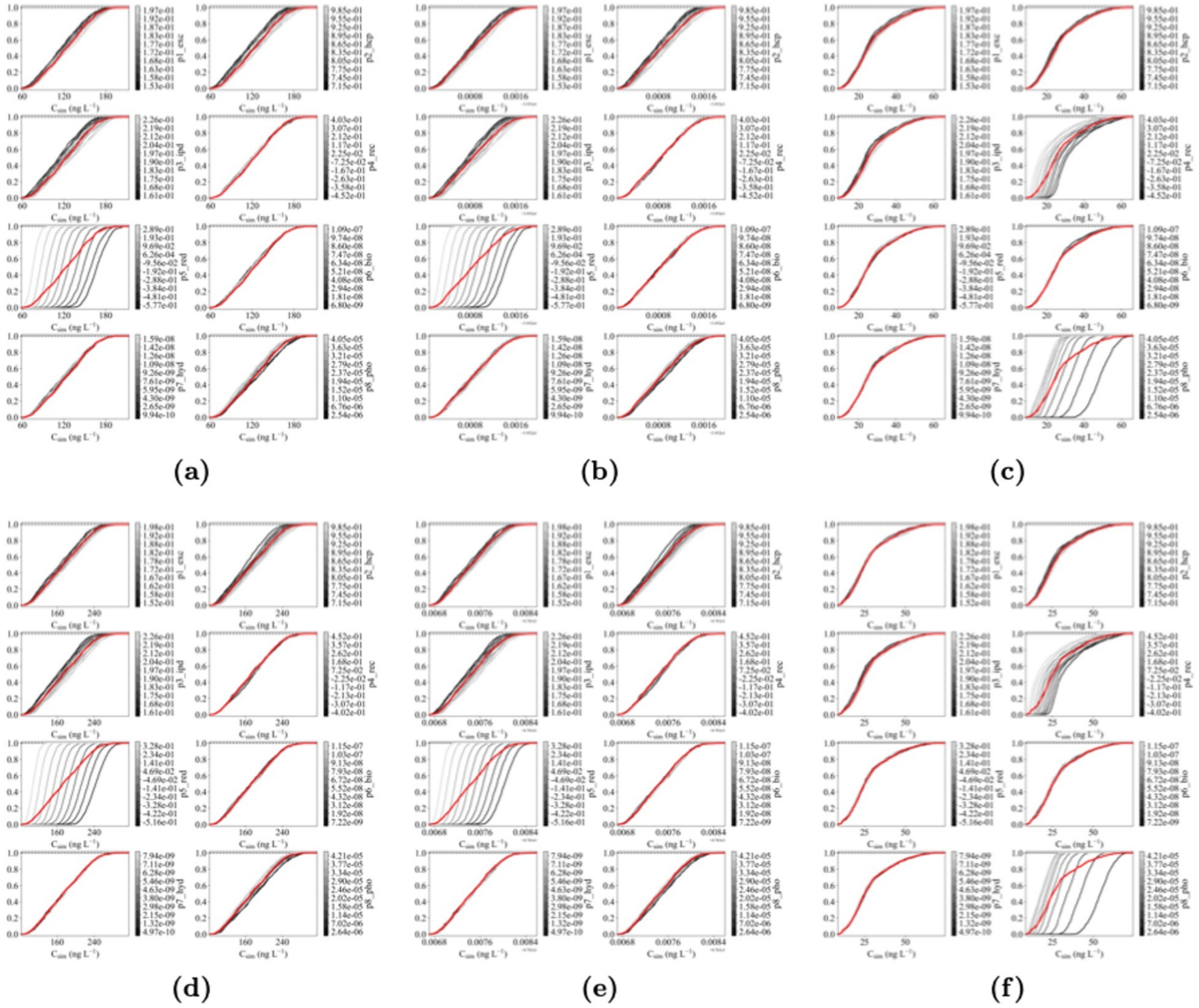

Figure S15: Cumulative distribution functions (CDFs) of the model factors for ciprofloxacin simulations at node $Linestring\_5000$ (a,d), node MST_8584 (b,e),node $WWTP\_5746$ (c,f) in the cool (a,b,c) and warm (d,e,f) seasons.

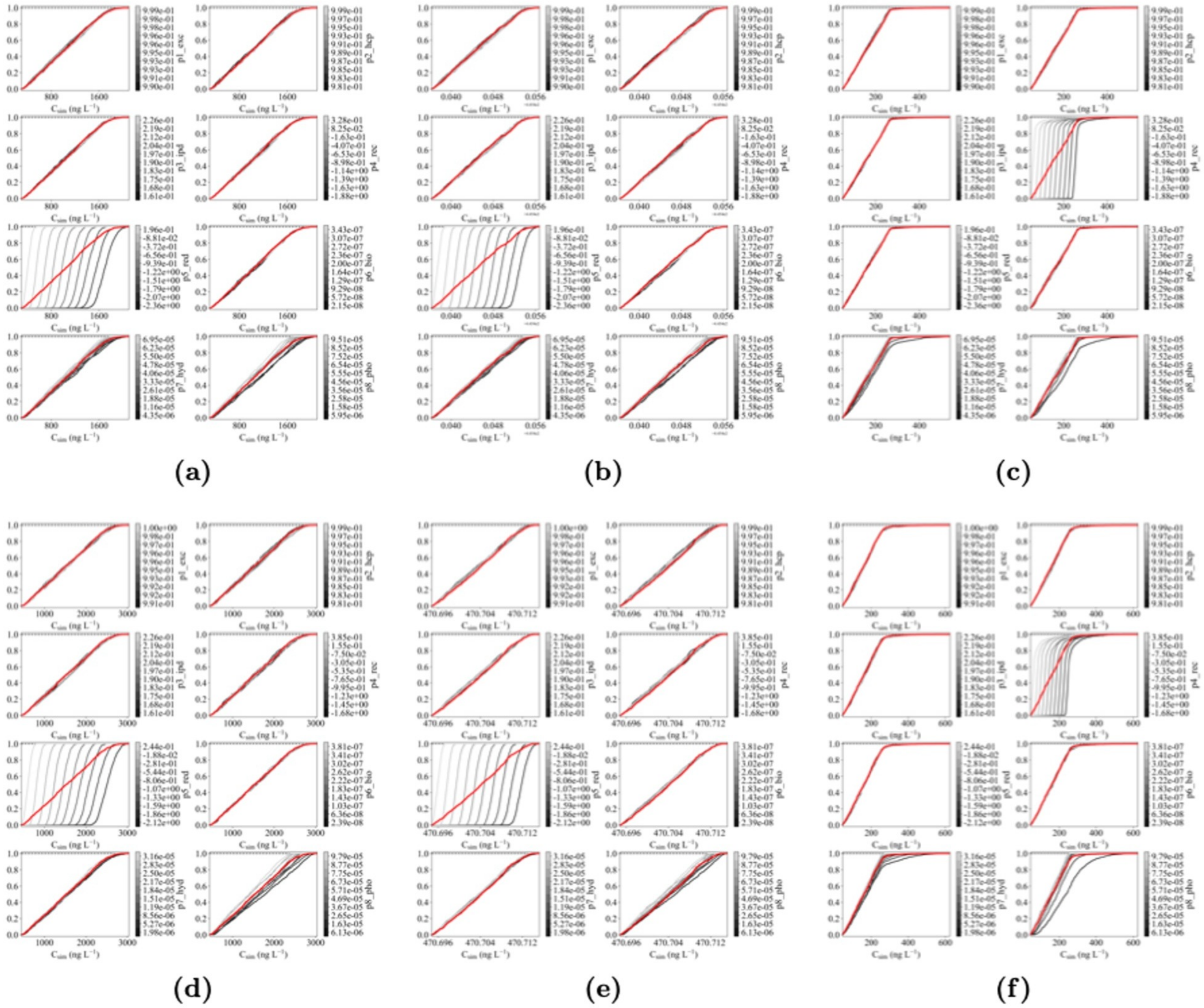

Figure S16: Cumulative distribution functions (CDFs) of the model factors for gabapentin simulations at node $Linestring\_5000$ (a,d), node MST_8584 (b,e),node $WWTP\_5746$ (c,f) in the cool (a,b,c) and warm (d,e,f) seasons.

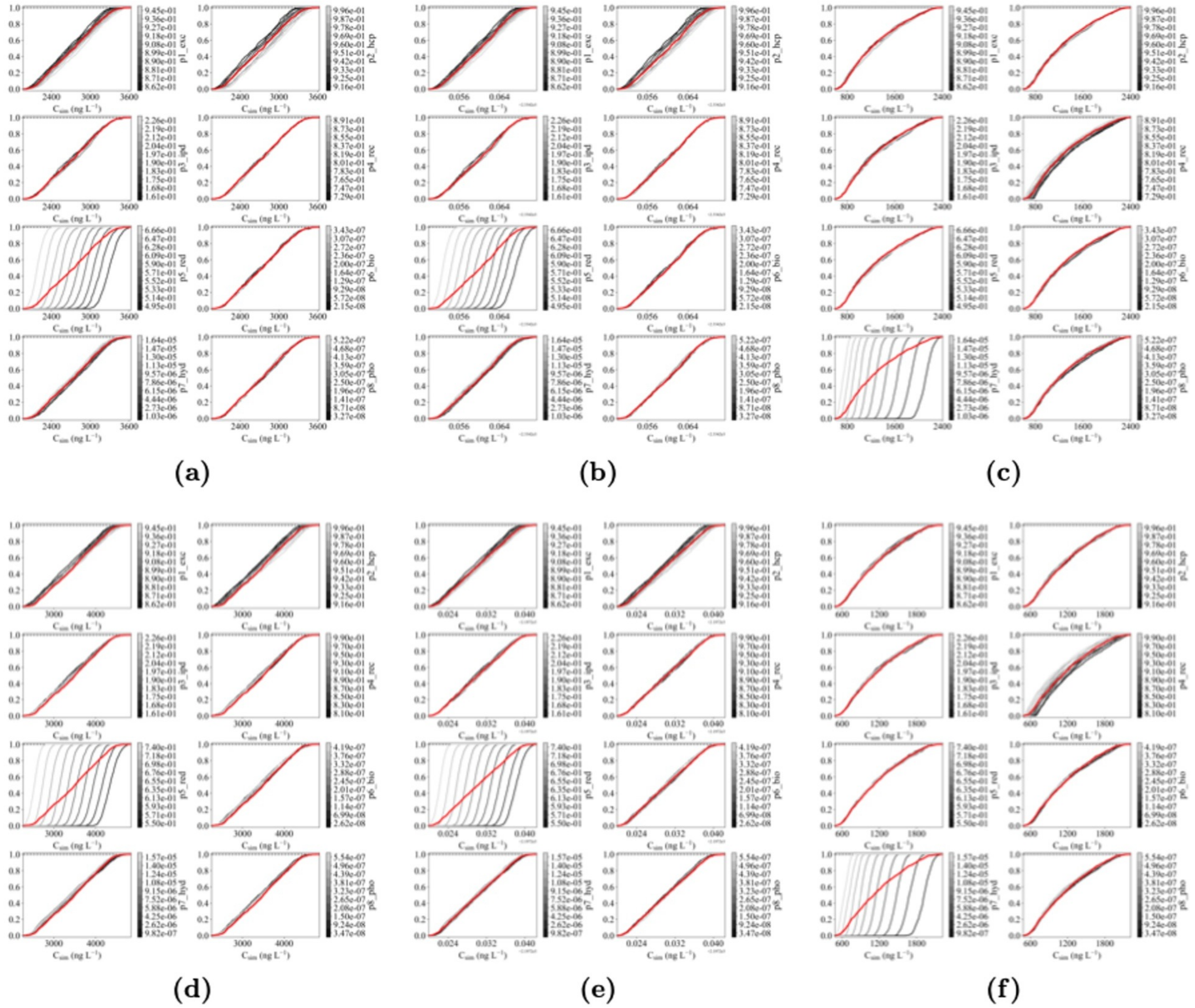

Figure S17: Cumulative distribution functions (CDFs) of the model factors for metformin simulations at node $Linestring\_5000$ **(a,d), node** MST_8584 **(b,e),node** $WWTP\_5746$ **(c,f) in the cool (a,b,c) and warm (d,e,f) seasons.**

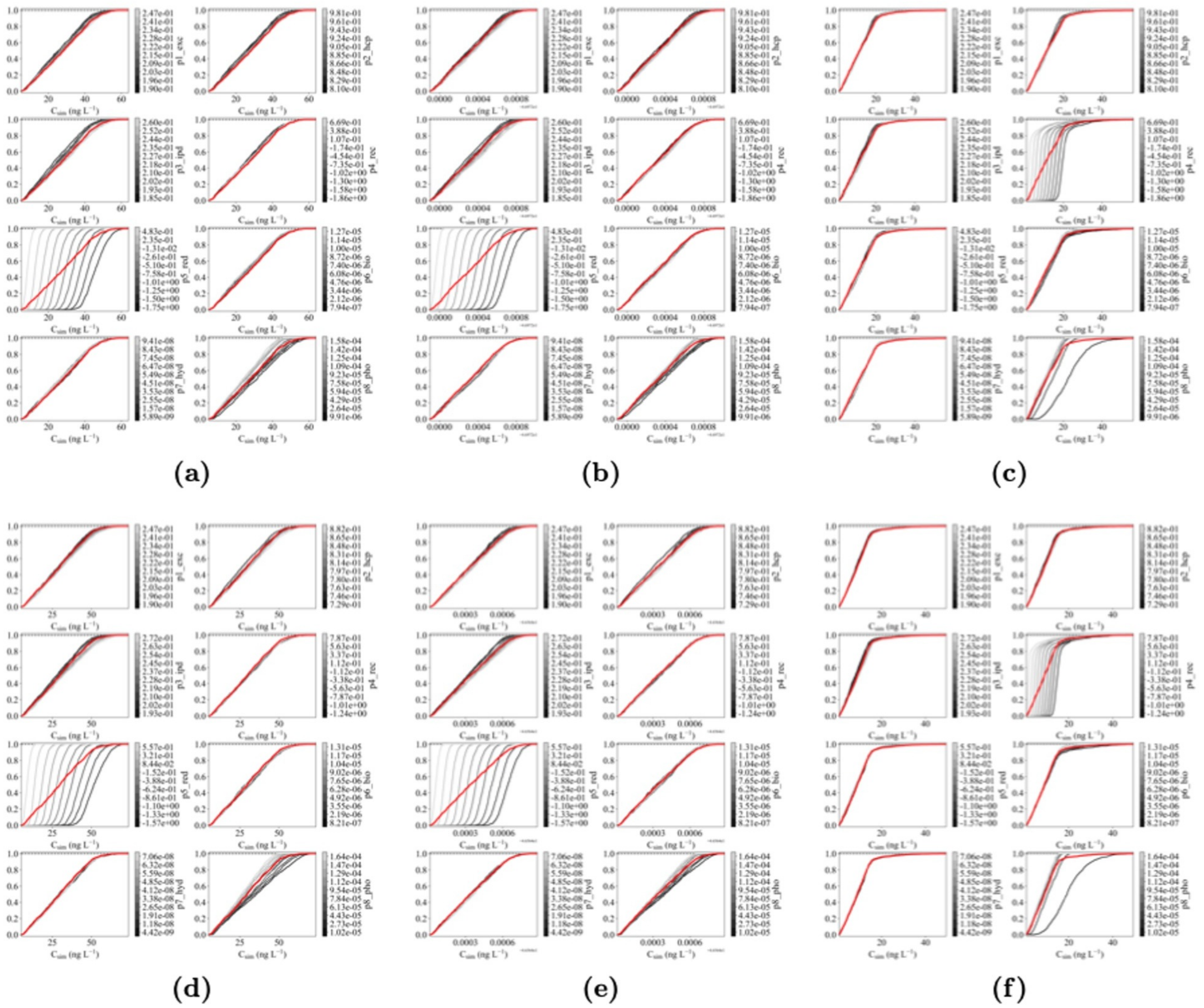

Figure S18: Cumulative distribution functions (CDFs) of the model factors for sulfamethoxazole simulations at node $Linestring\_5000$ (a,d), node MST_8584 (b,e),node $WWTP\_5746$ (c,f) in the cool (a,b,c) and warm (d,e,f) seasons.

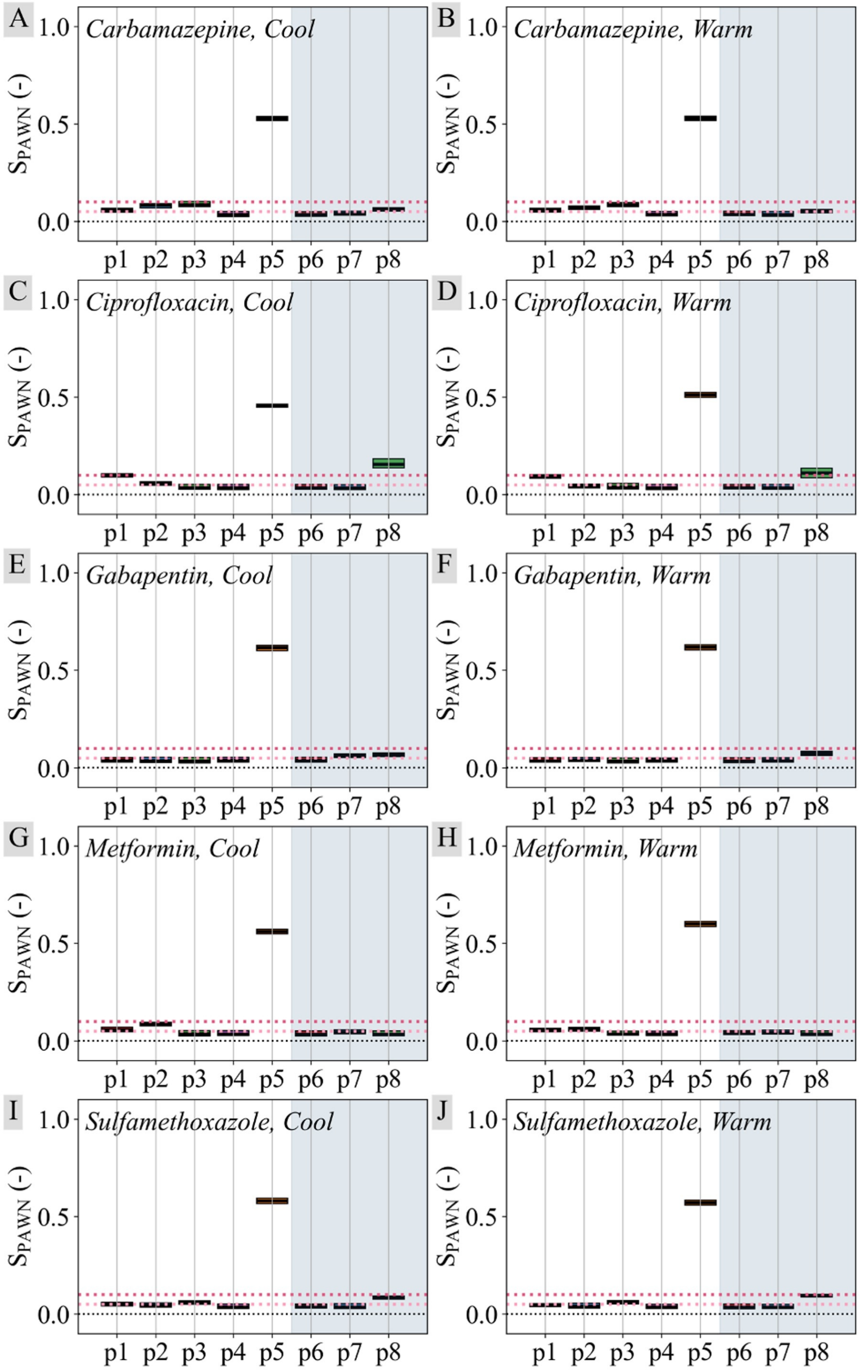

Figure S19: PAWN sensitivity indices S_{PAWN} of the eight model factors and their 95% bootstrap confidence intervals at node *Linestring*_5000**.** Warm season: May−October; Cool season: November−April. The dashed light pink and dashed dark pink lines represent 0.05 and 0.1, respectively, which are adopted to classify low, moderate, and high sensitivity level. $p1$: human excretion rate; $p2$: human compliance rate; $p3$: improper disposal rate; $p4$: removal efficiency in WWTPs (centralized wastewater treatment plants); $p5$: removal efficiency in DWTSs (decetralized wastewater treatment systems); $p6$: biodegradation; $p7$: hydrolysis; $p8$: photolysis.

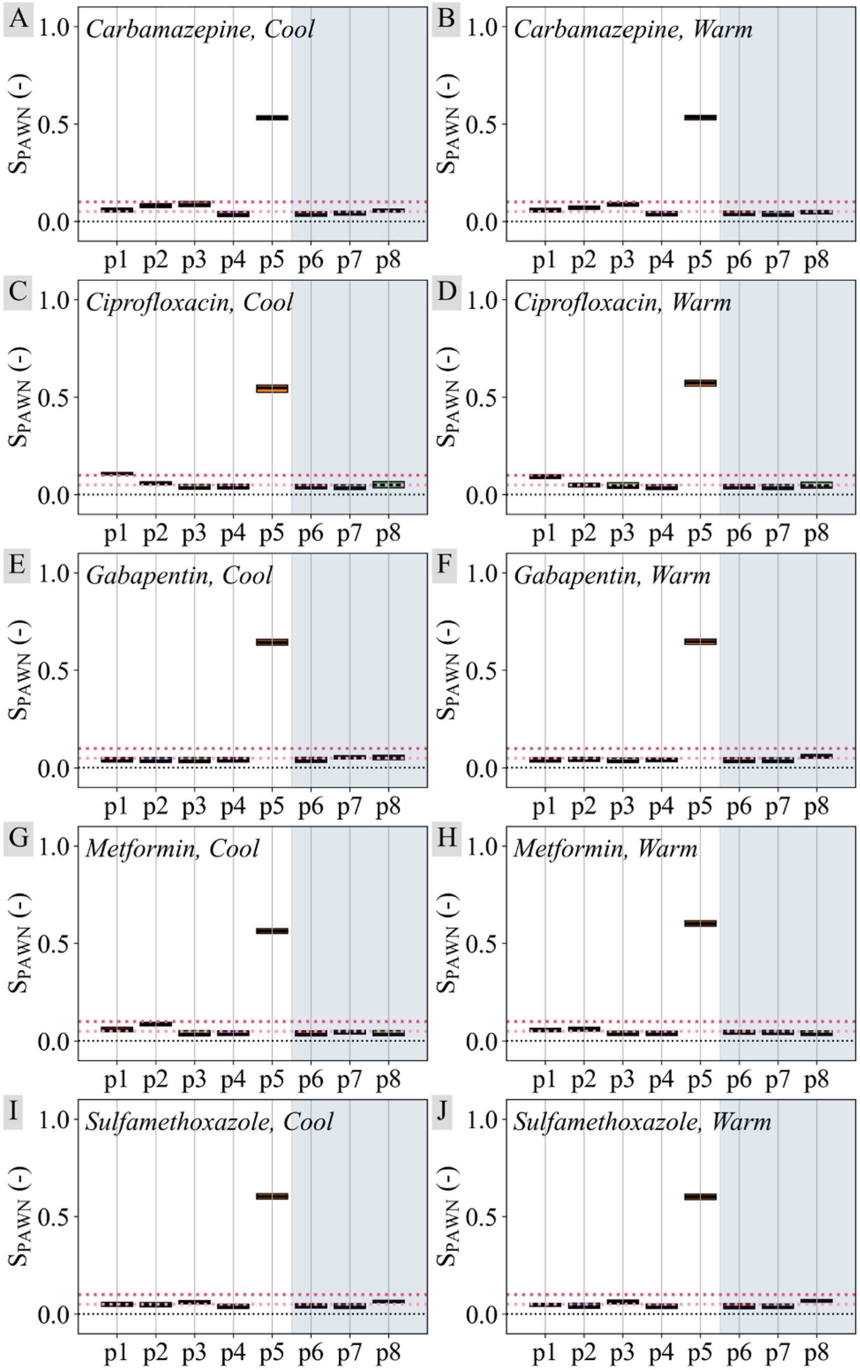

Figure S20: PAWN sensitivity indices S_{PAWN} of the eight model factors and their 95% bootstrap confidence intervals at node MST_8584. Warm season: May−October; Cool season: November−April. The dashed light pink and dashed dark pink lines represent 0.05 and 0.1, respectively, which are adopted to classify low, moderate, and high sensitivity level. $p1$: human excretion rate; $p2$: human compliance rate; $p3$: improper disposal rate; $p4$: removal efficiency in WWTPs (centralized wastewater treatment plants); $p5$: removal efficiency in DWTSs (decentralized wastewater treatment systems); $p6$: biodegradation; $p7$: hydrolysis; $p8$: photolysis.

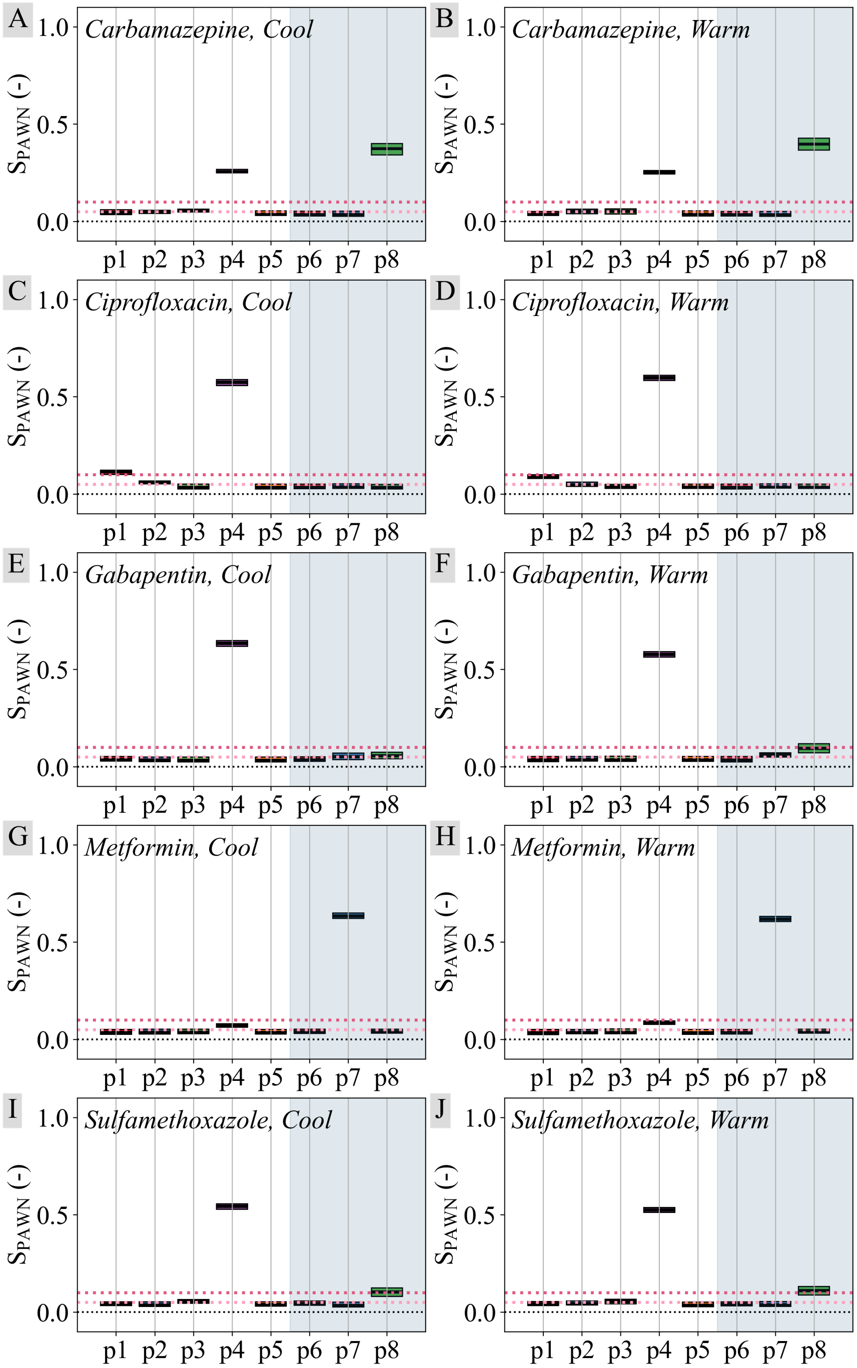

Figure S21: PAWN sensitivity indices S_{PAWN} of the eight model factors and their 95% bootstrap confidence intervals at node $WWTP\_5746$. Warm season: May−October; Cool season: November−April. The dashed light pink and dashed dark pink lines represent 0.05 and 0.1, respectively, which are adopted to classify low, moderate, and high sensitivity level. $p1$: human excretion rate; $p2$: human compliance rate; $p3$: improper disposal rate; $p4$: removal efficiency in WWTPs (centralized wastewater treatment plants); $p5$: removal efficiency in DWTSs (decentralized wastewater treatment systems); $p6$: biodegradation; $p7$: hydrolysis; $p8$: photolysis.

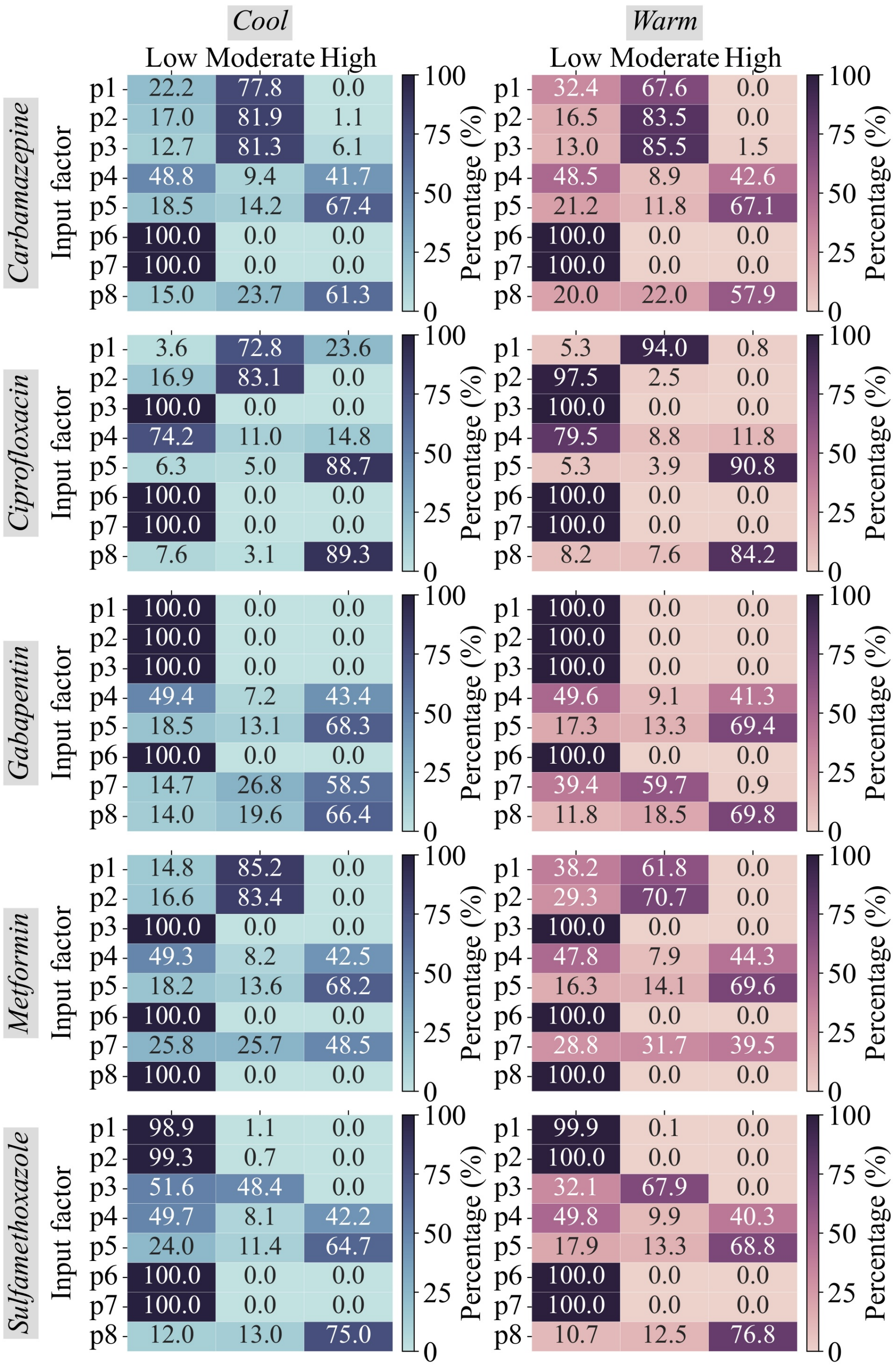

Figure S22: Percentage of rivers exhibiting different sensitivity levels for each input factor. Low, moderate, and high sensitivity are divided by the threshold of S_{PAWN} (-) 0.05 and 0.1, respectively. Input factors include, $p1 - fexc$: human excretion rate; $p2 - f_{hcp}$: human compliance rate; $p3 - f_{ipd}$: improper disposal rate; $p4 - fw\_c$: removal efficiency in centralized WWTPs; $p5 - f_{w\_d}$: removal efficiency in decentralized DWTSs; $p6 - f_{bio}$: biodegradation; $p7 - f_{hyd}$: hydrolysis; $p8 - f_{pho}$: photolysis. Refer to Table S3 for more details on the input factors. Warm season: May-October; Cool season: November-April.

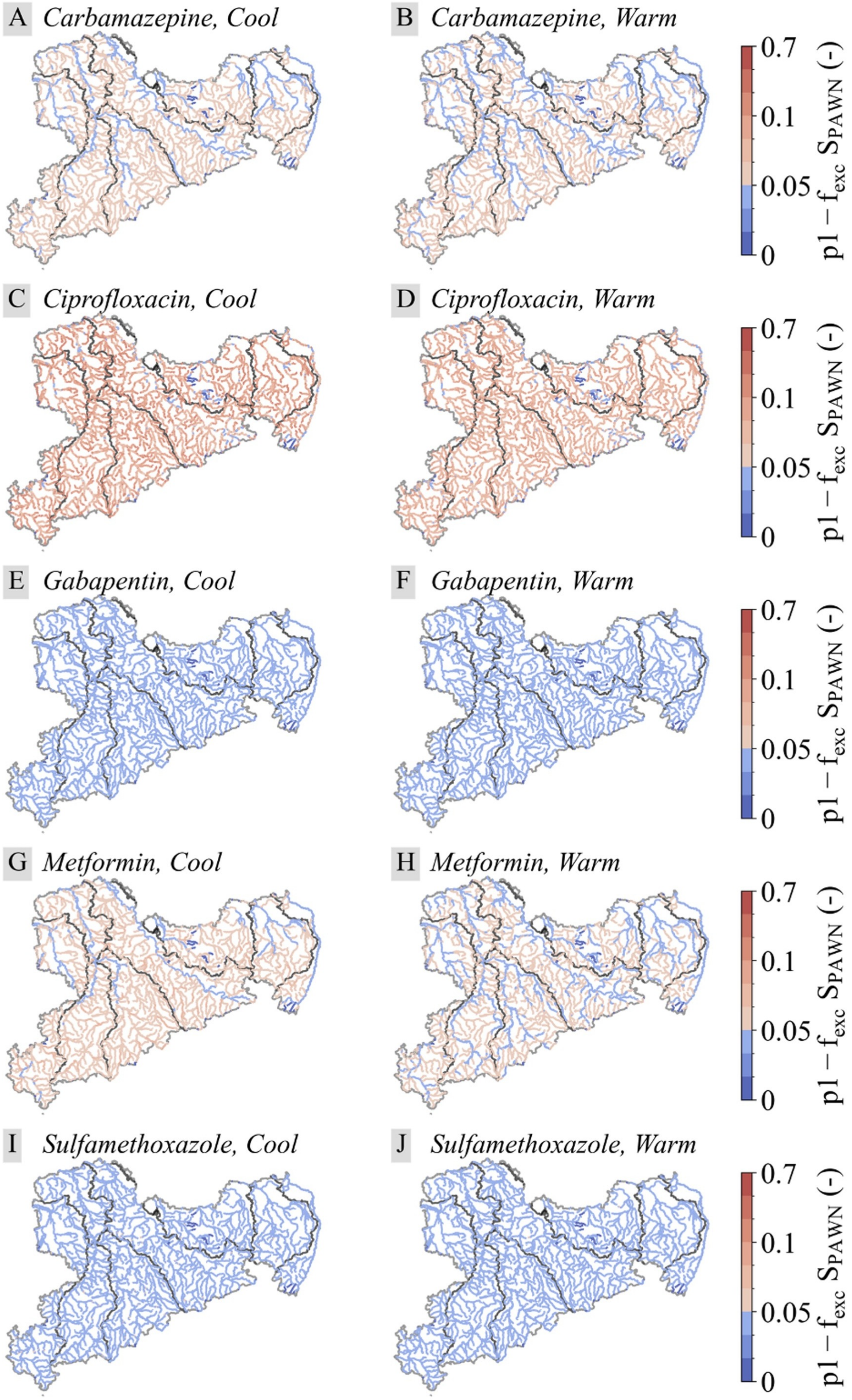

Figure S23: PAWN sensitivity indices S_{PAWN} of parameter human excretion rate ($p1 - f_{exc}$). Warm season: May−October; Cool season: November−April.

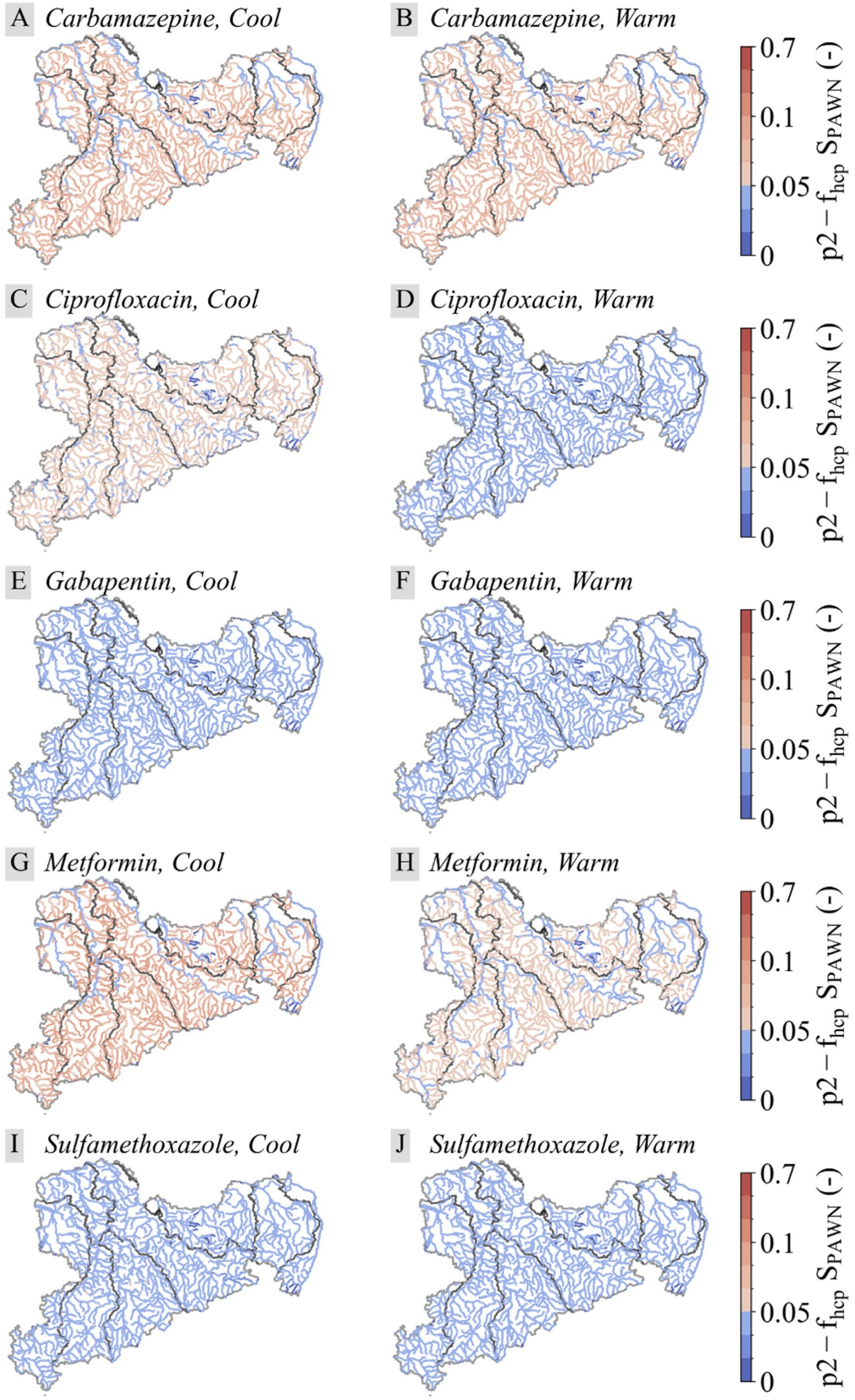

Figure S24: PAWN sensitivity indices S_{PAWN} of parameter human compliance rate ($p2 - f_{hcp}$). Warm season: May−October; Cool season: November−April.

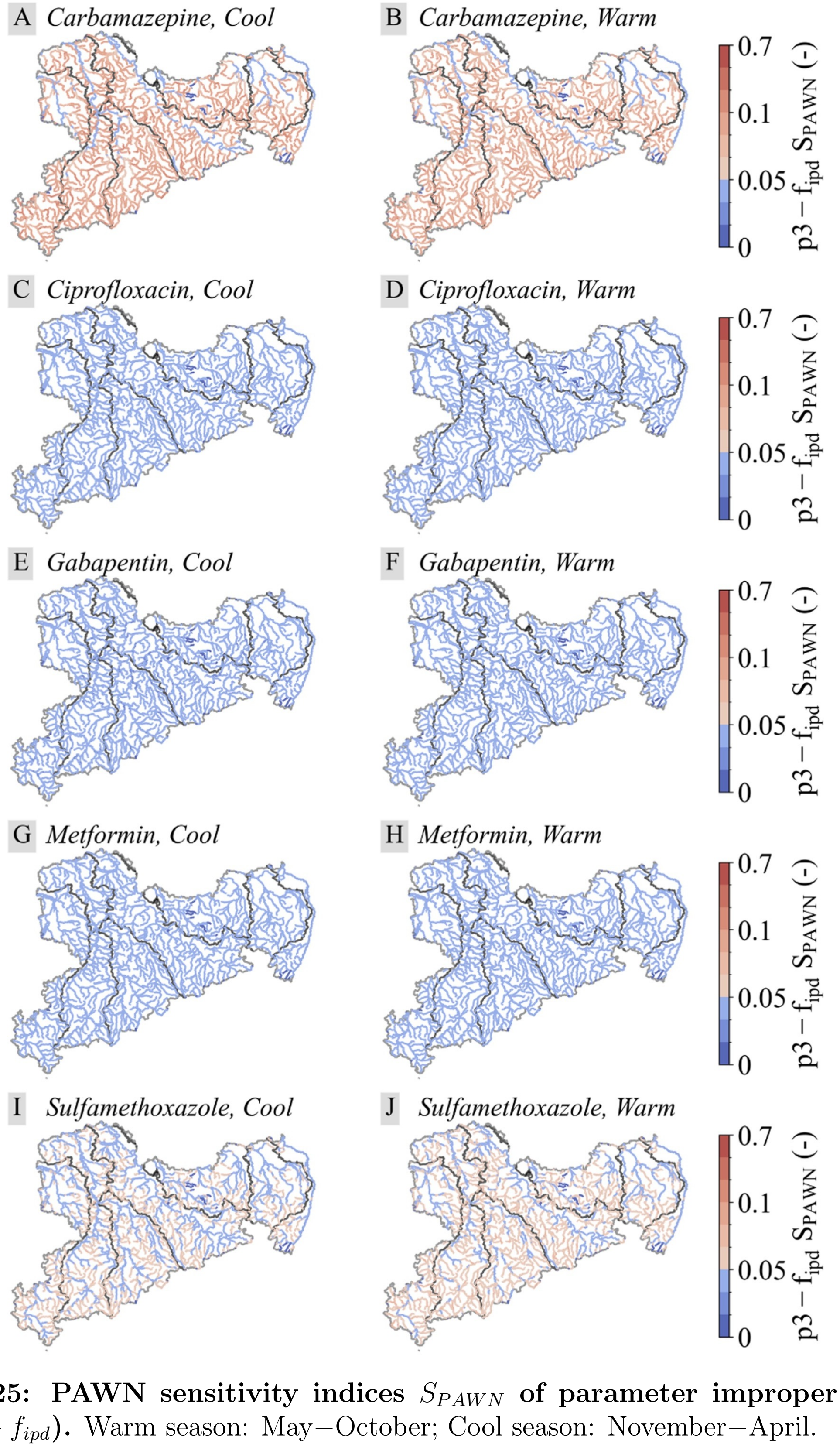

Figure S25: PAWN sensitivity indices S_{PAWN} of parameter improper disposal rate ($p3 - f_{ipd}$). Warm season: May−October; Cool season: November−April.

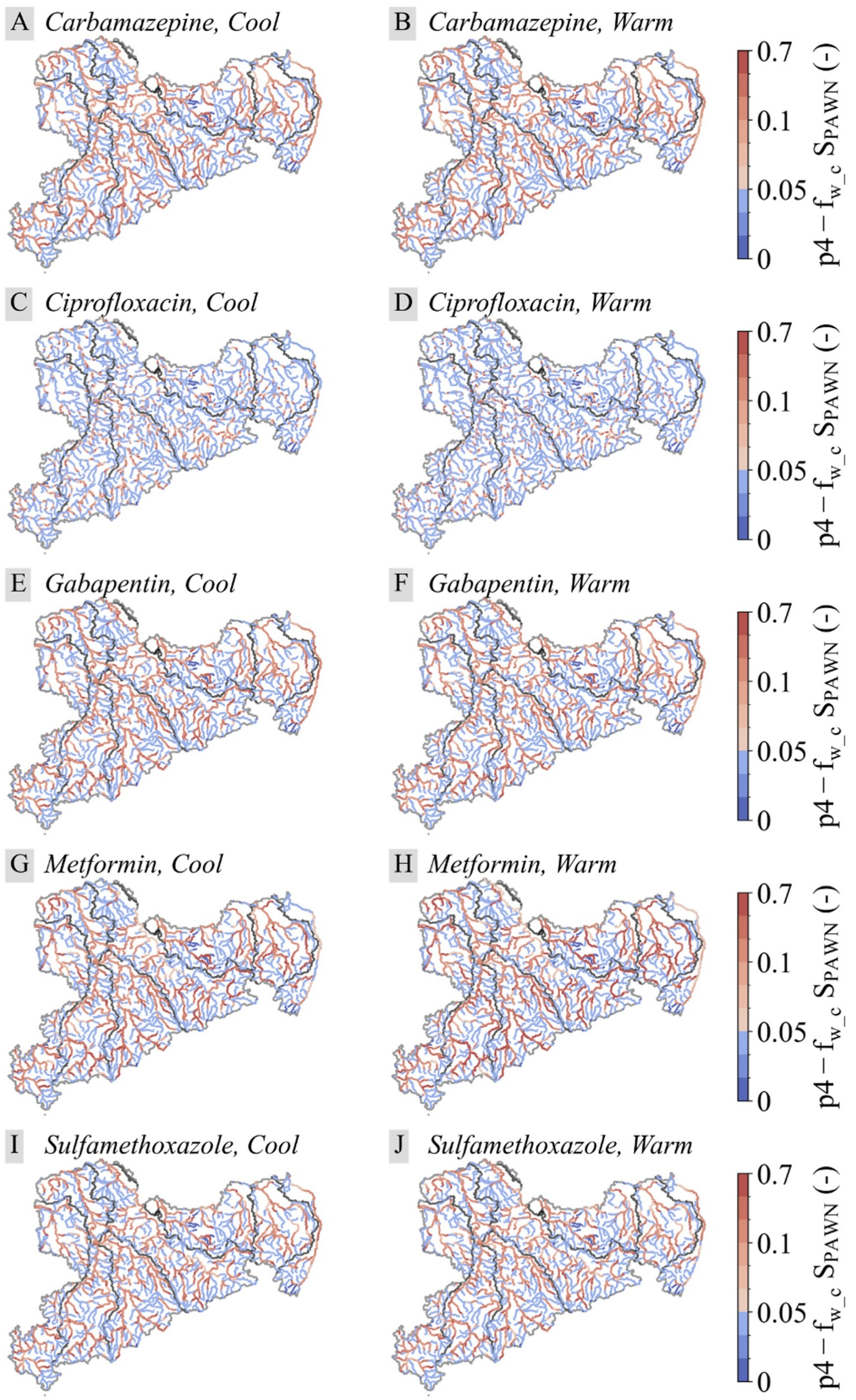

Figure S26: PAWN sensitivity indices S_{PAWN} of parameter removal efficiency in centralized wastewater treatment plants ($p4 - f_{w\_c}$). Warm season: May−October; Cool season: November−April.

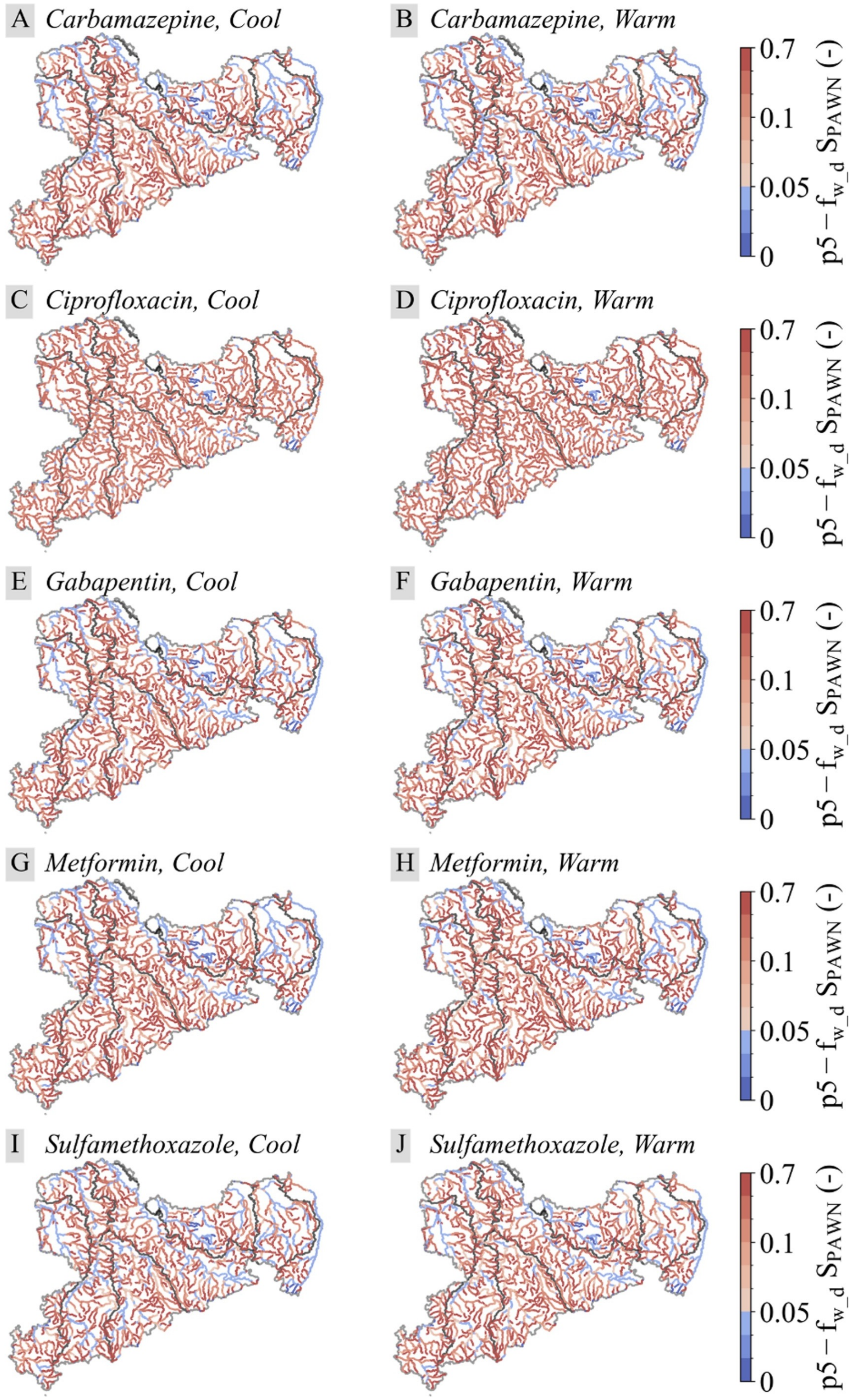

Figure S27: PAWN sensitivity indices S_{PAWN} of parameter removal efficiency in decentralized wastewater treatment plants ($p5-f_{w\_d}$). Warm season: May−October; Cool season: November−April.

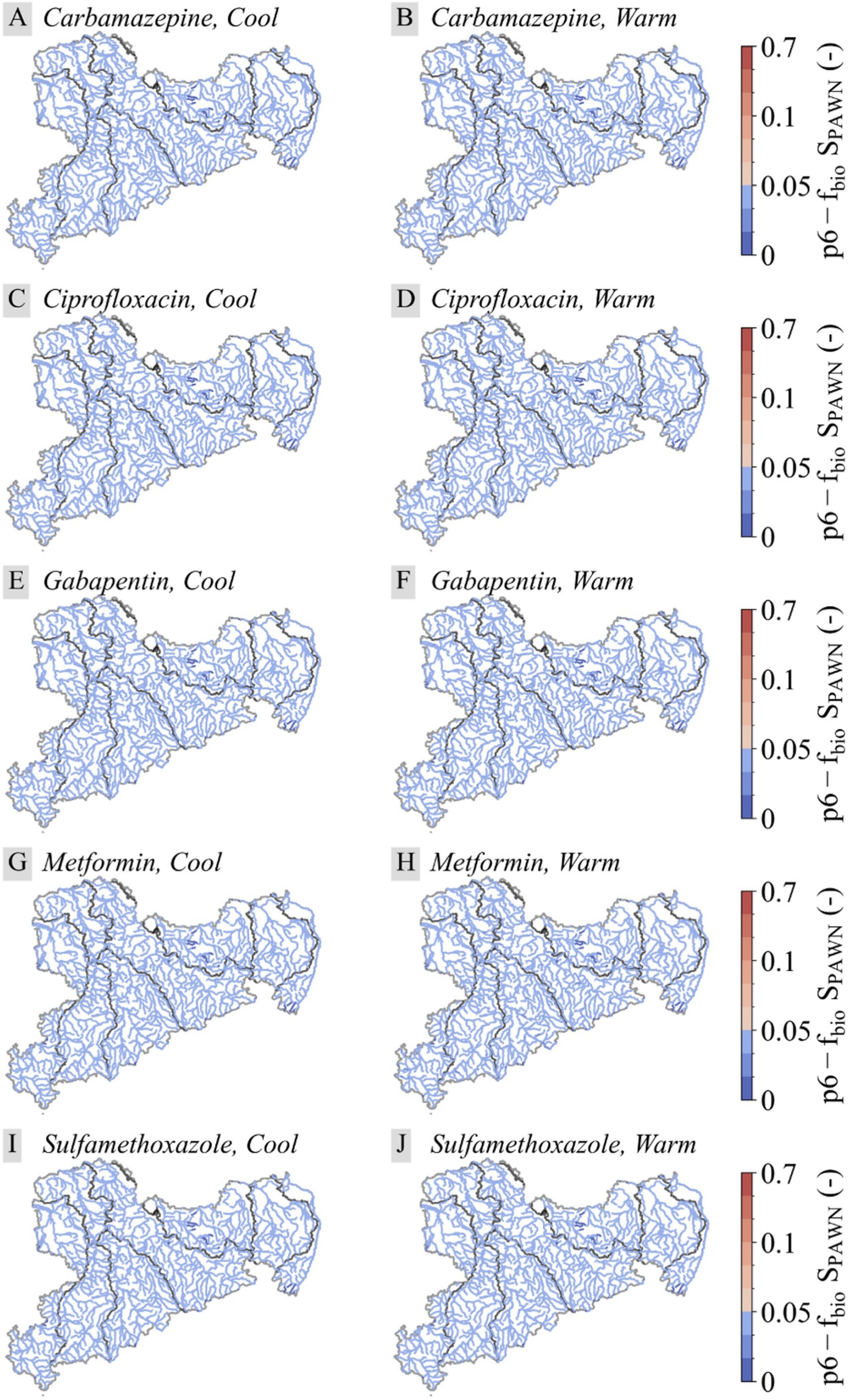

Figure S28: PAWN sensitivity indices S_{PAWN} of parameter biodegradation rate ($p6 - f_{bio}$). Warm season: May−October; Cool season: November−April.

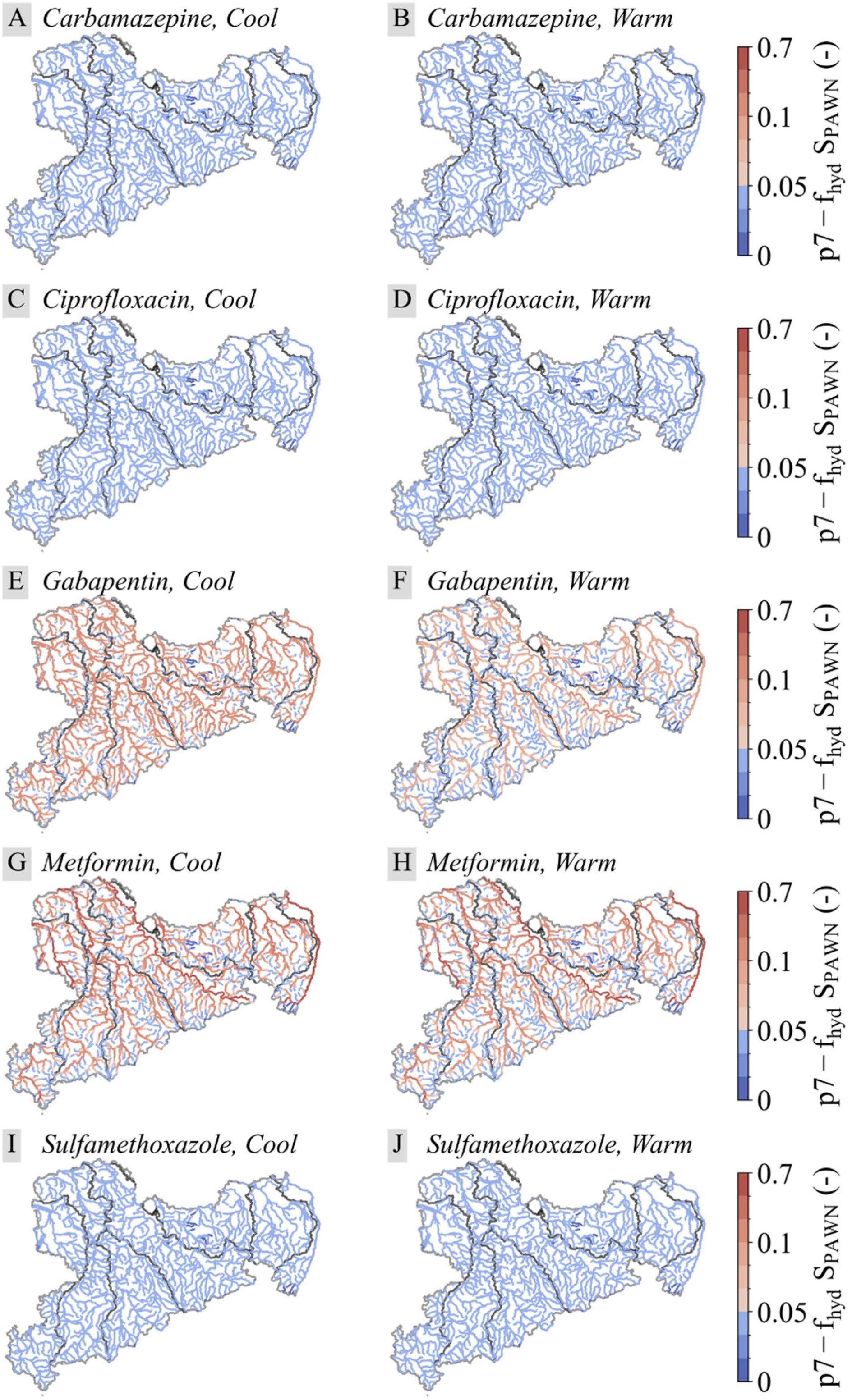

Figure S29: PAWN sensitivity indices S_{PAWN} of parameter hydrolysis rate ($p7-f_{hyd}$). Warm season: May−October; Cool season: November−April.

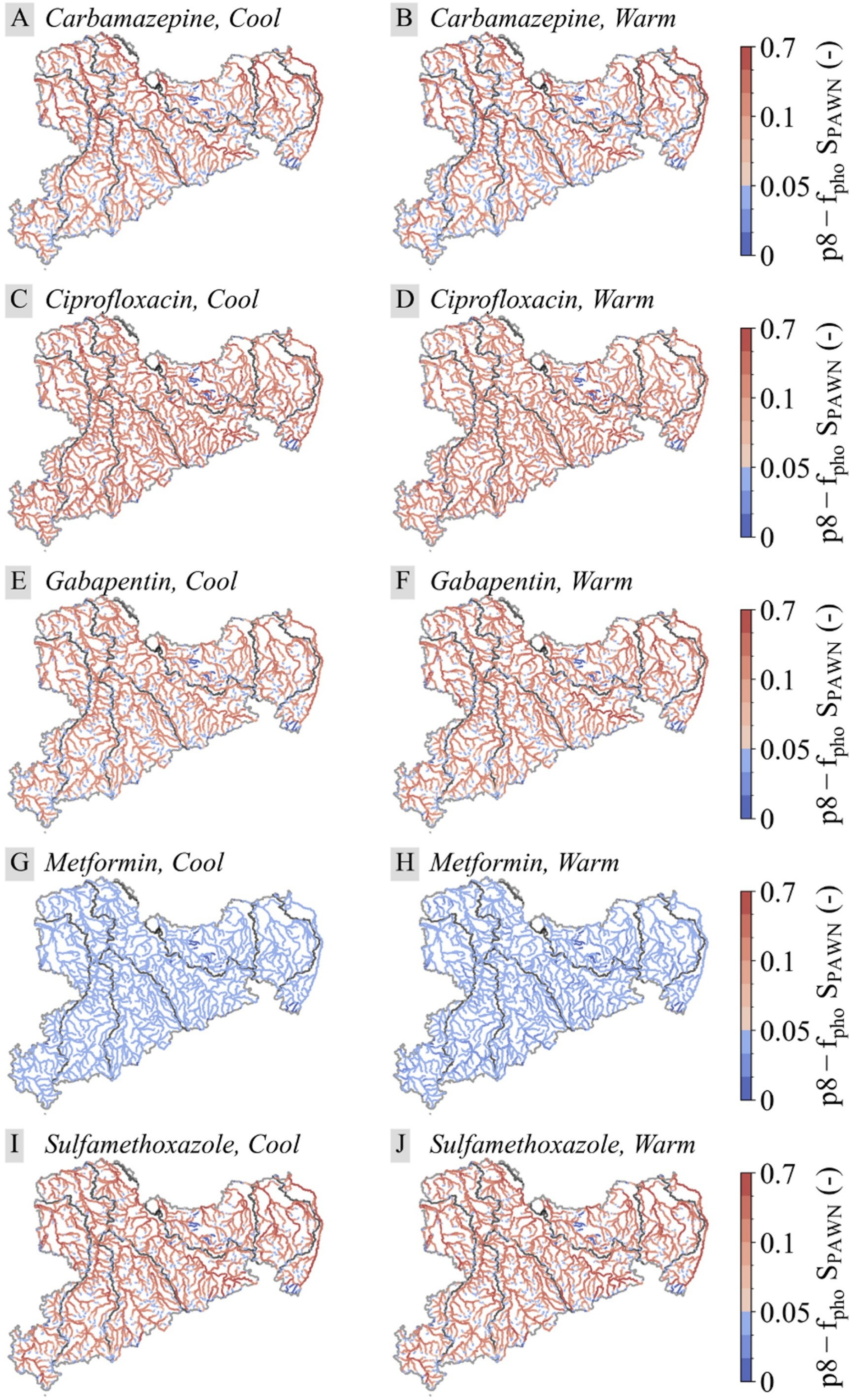

Figure S30: PAWN sensitivity indices S_{PAWN} of parameter photolysis rate ($p8 - f_{pho}$). Warm season: May−October; Cool season: November−April.

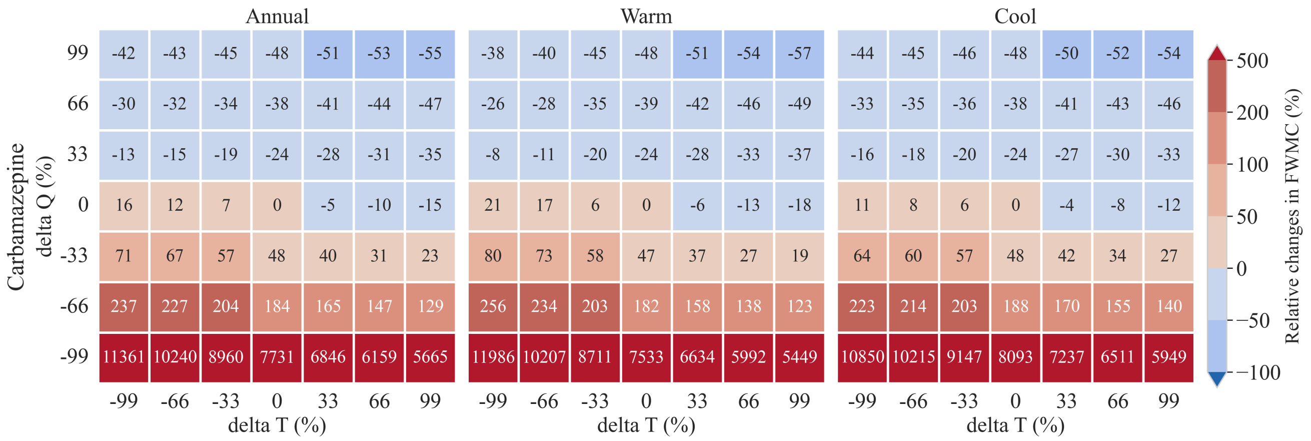

Figure S31: Relative changes (%) in median flow-weighted mean carbamazepine concentrations (FWMC) under varying hydrological (Q) and temperature-related (T, represented by biodegradation and photolysis rates) scenarios, compared to current model values.

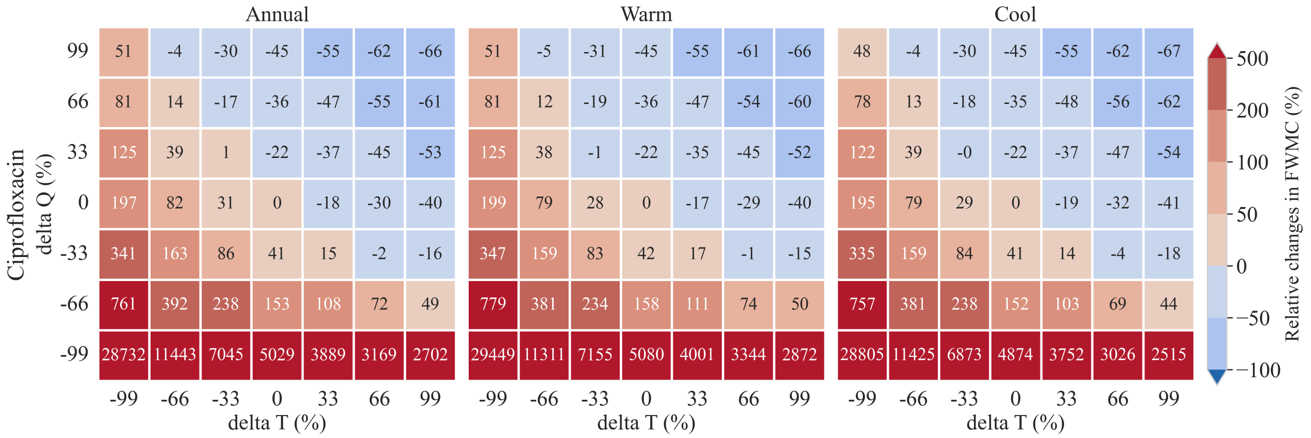

Figure S32: Relative changes (%) in median flow-weighted mean ciprofloxacin concentrations (FWMC) under varying hydrological (Q) and temperature-related (T, represented by biodegradation and photolysis rates) scenarios, compared to current model values.

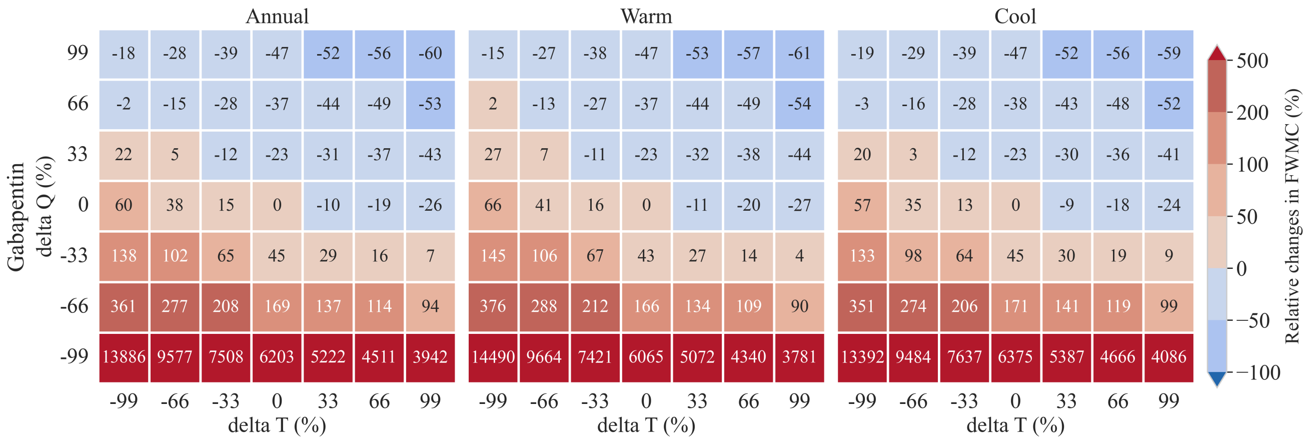

Figure S33: Relative changes (%) in median flow-weighted mean gabapentin concentrations (FWMC) under varying hydrological (Q) and temperature-related (T, represented by biodegradation and photolysis rates) scenarios, compared to current model values.

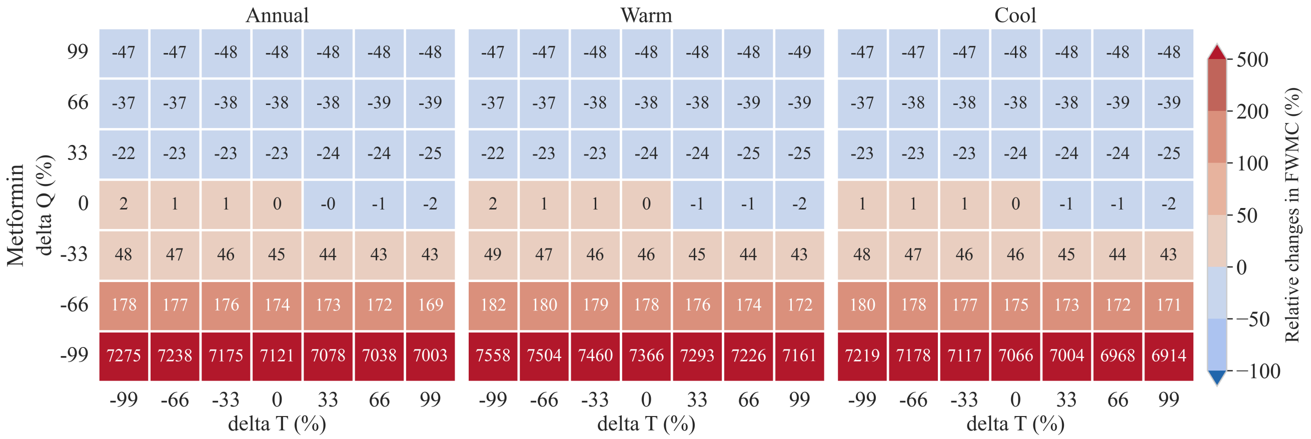

Figure S34: Relative changes (%) in median flow-weighted mean metformin concentrations (FWMC) under varying hydrological (Q) and temperature-related (T, represented by biodegradation and photolysis rates) scenarios, compared to current model values.

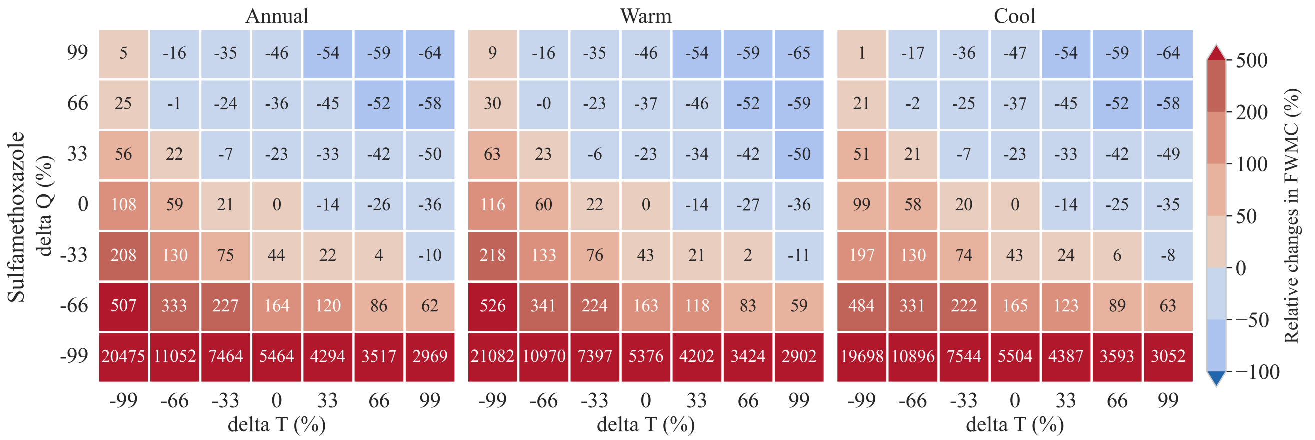

Figure S35: Relative changes (%) in median flow-weighted mean sulfamethoxazole concentrations (FWMC) under varying hydrological (Q) and temperature-related (T, represented by biodegradation and photolysis rates) scenarios, compared to current model values.

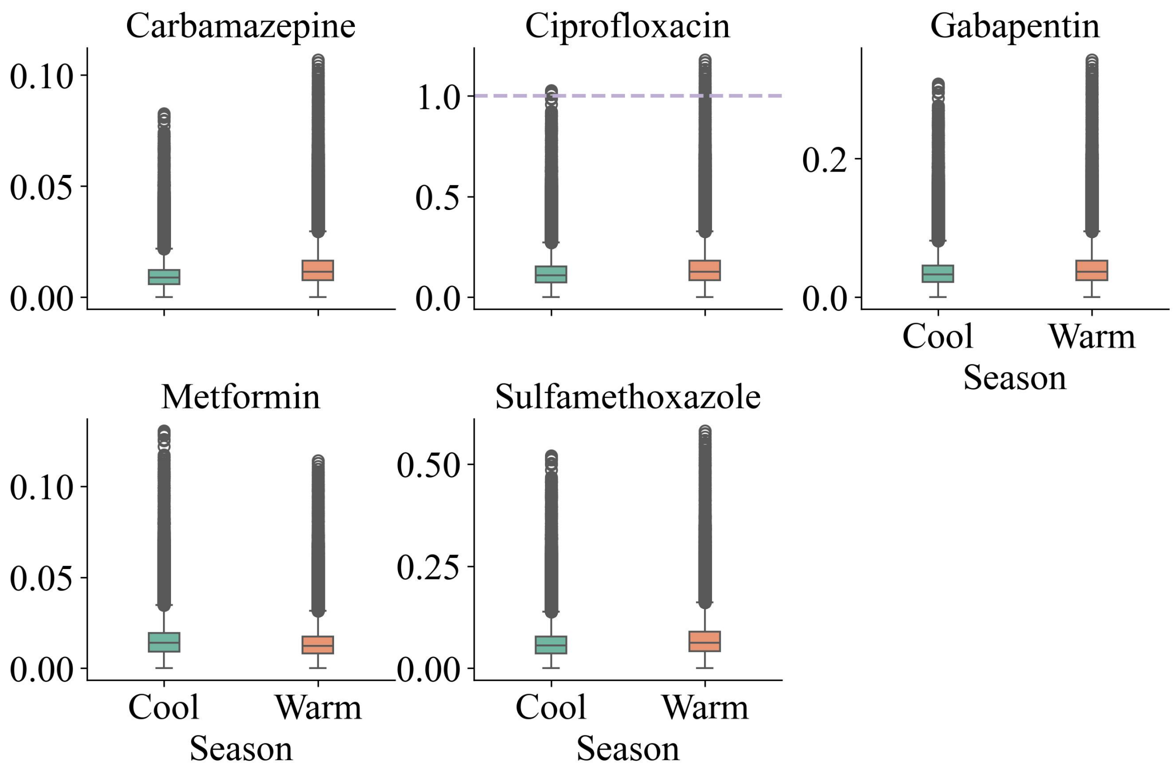

Figure S36: Damköhler number in the cool and warm seasons across pharmaceuticals.

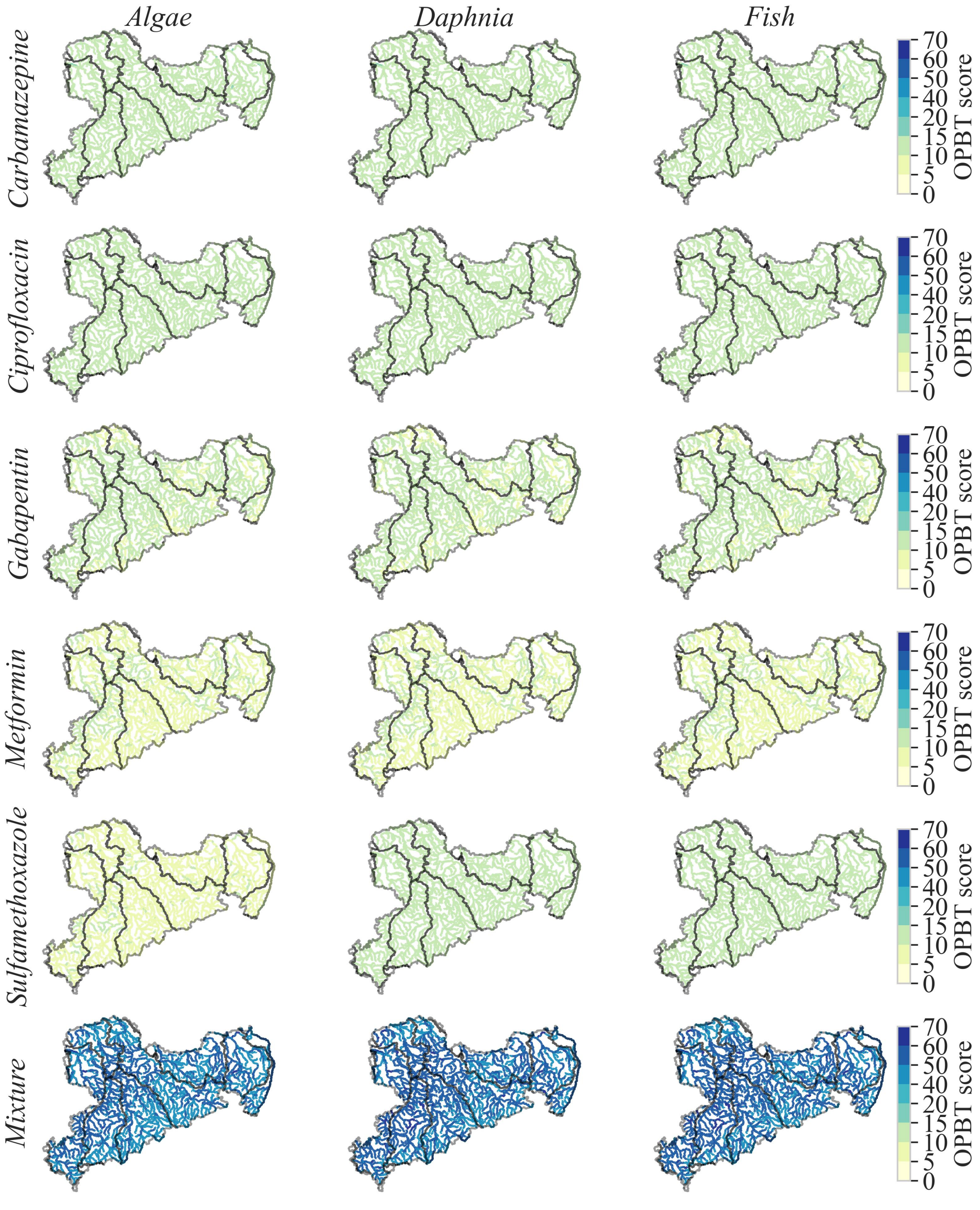

Figure S37: Spatial distribution of the total OPBT score across pharmaceuticals and species in Saxon rivers during the cool (November–April) season. O: occurrence, P: persistence, B: bioaccumulation, T: toxicity.

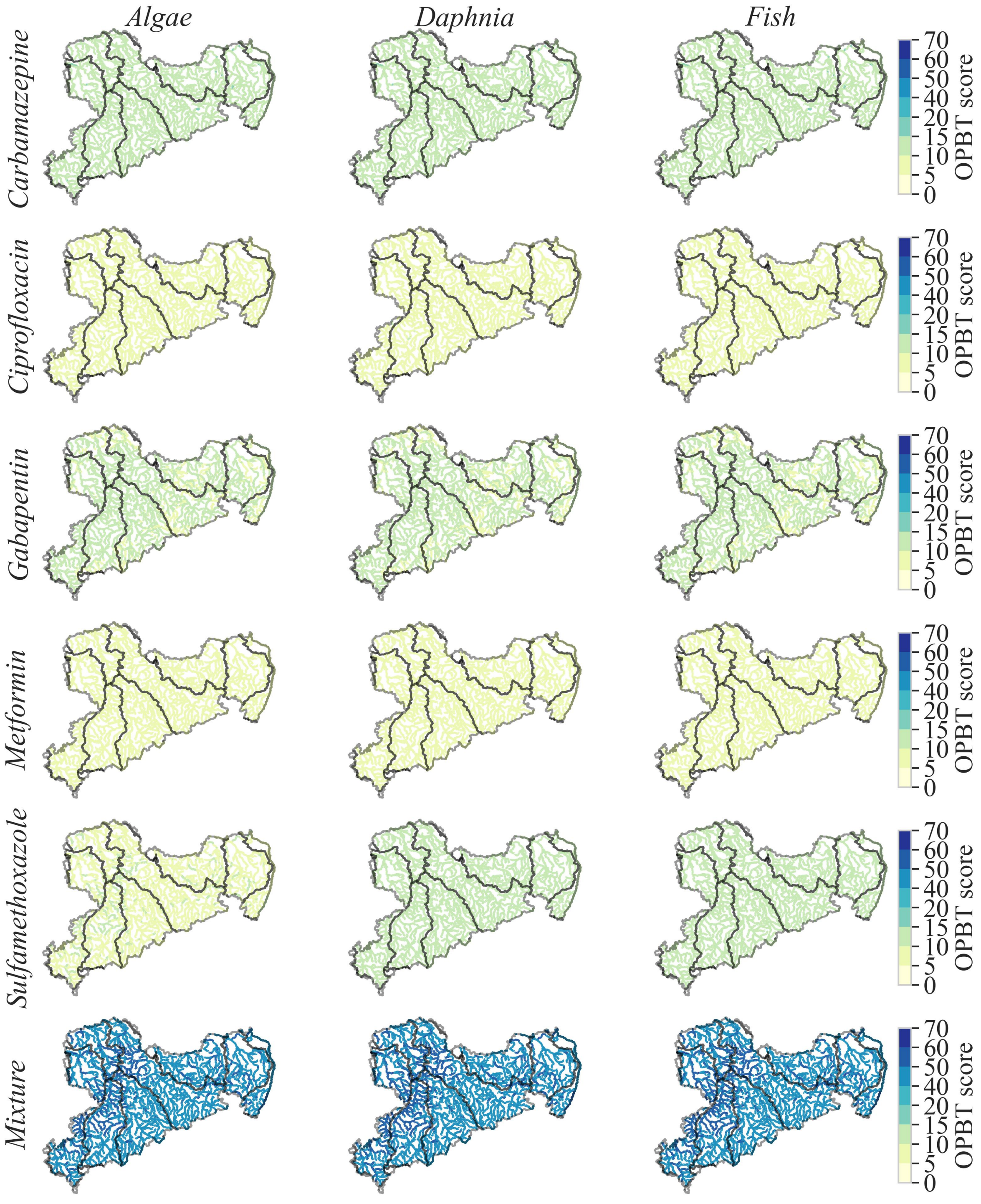

Figure S38: Spatial distribution of the total OPBT score across pharmaceuticals and species in Saxon rivers during the warm (May–October) season. O: occurrence, P: persistence, B: bioaccumulation, T: toxicity.

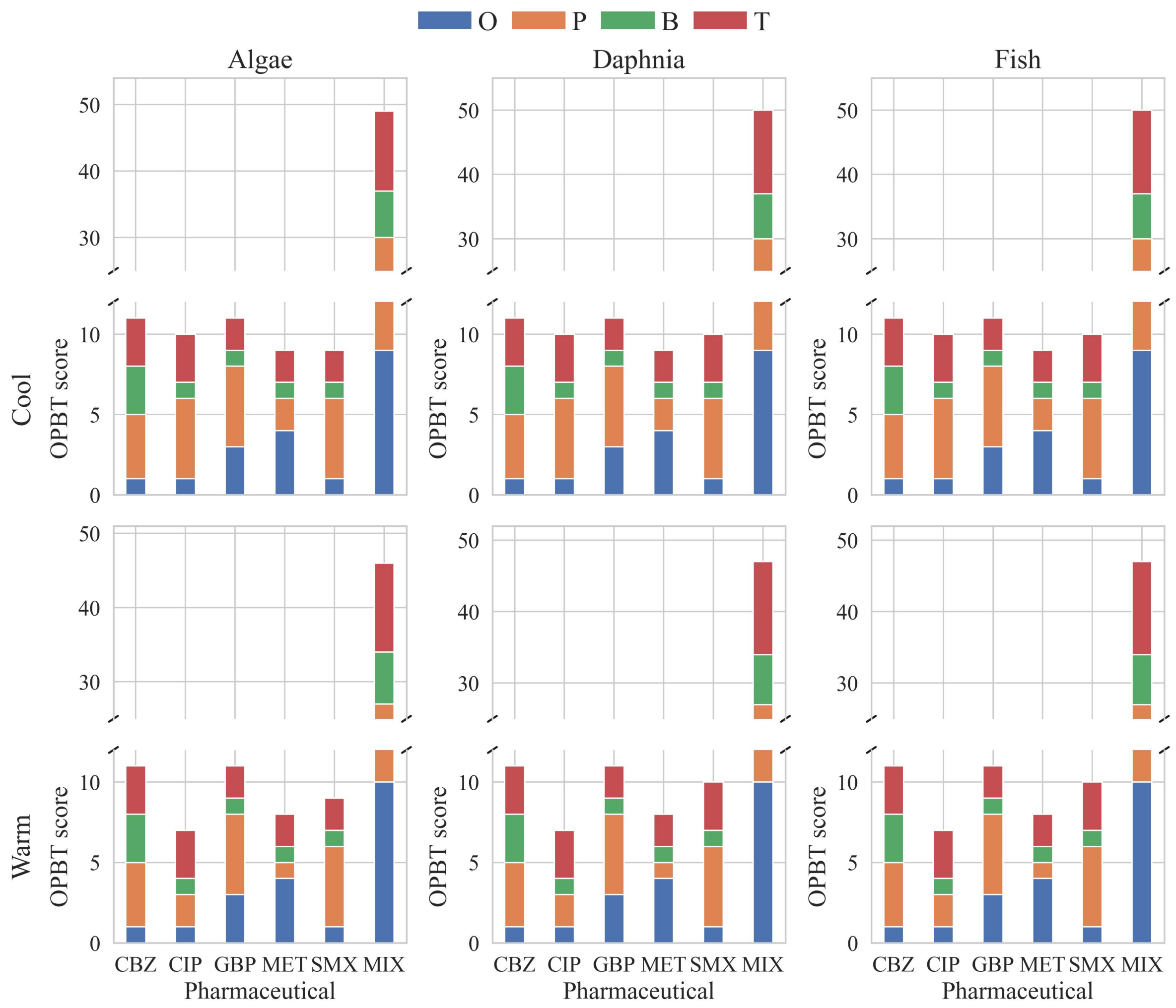

Figure S39: Seasonal comparison of median OPBT component scores for each pharmaceutical and species in Saxon rivers. Bars represent the stacked median values of occurrence (O), persistence (P), bioaccumulation (B), and toxicity (T), illustrating the composition of the total OPBT score in the cool (November–April) and warm (May–October) seasons. Pharmaceutical abbreviations: CBZ (carbamazepine), CIP (ciprofloxacin), GBP (gabapentin), MET (metformin), SMX (sulfamethoxazole), MIX (mixture).

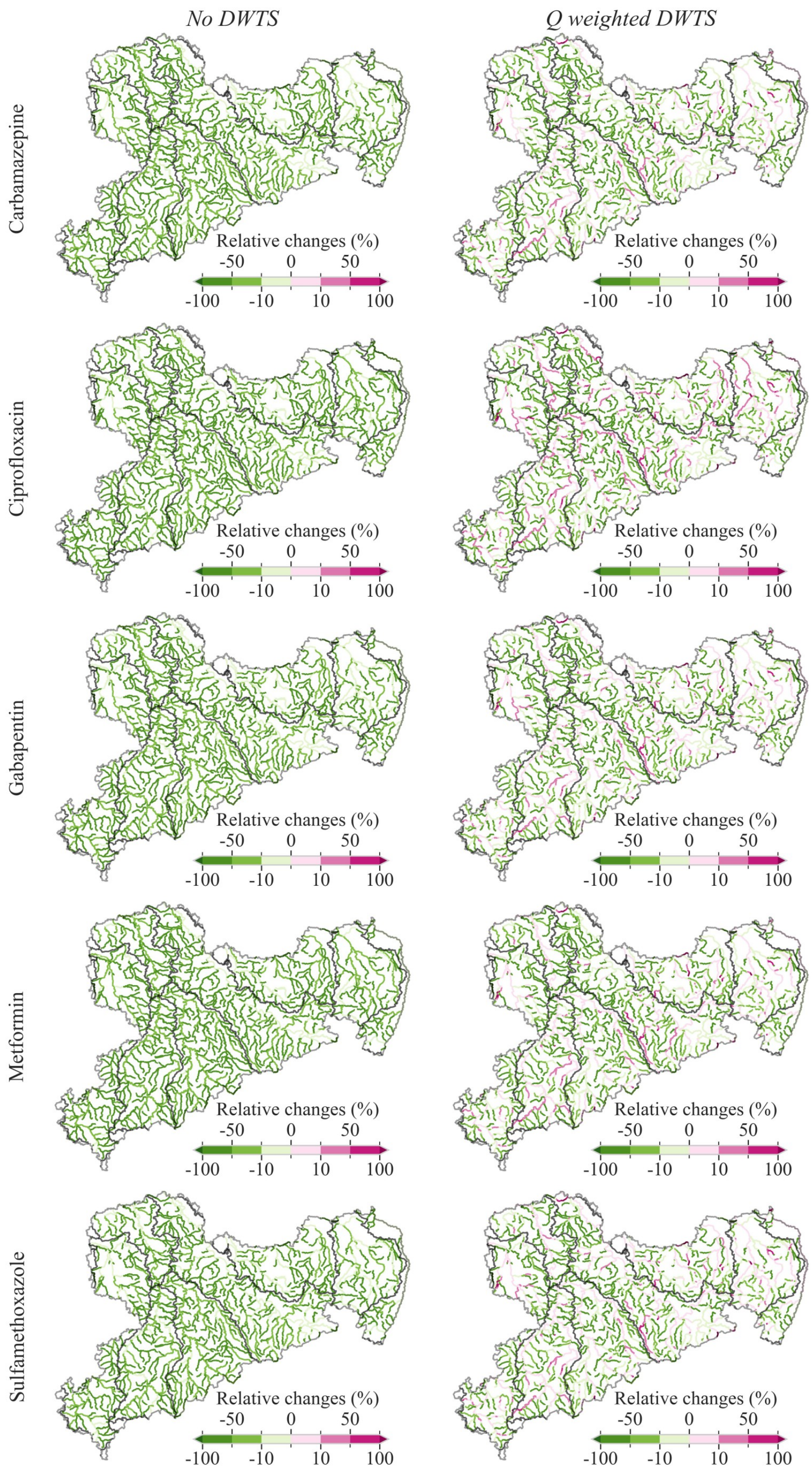

Figure S40: Spatial distribution of relative differences (%) in pharmaceutical concentrations for two DWTS scenarios compared to the original uniform distribution assumption. Left: Scenario excluding all DWTS emissions. Right: Scenario with DWTS emissions redistributed to river nodes using discharge augmented (Q-weighted) allocation.

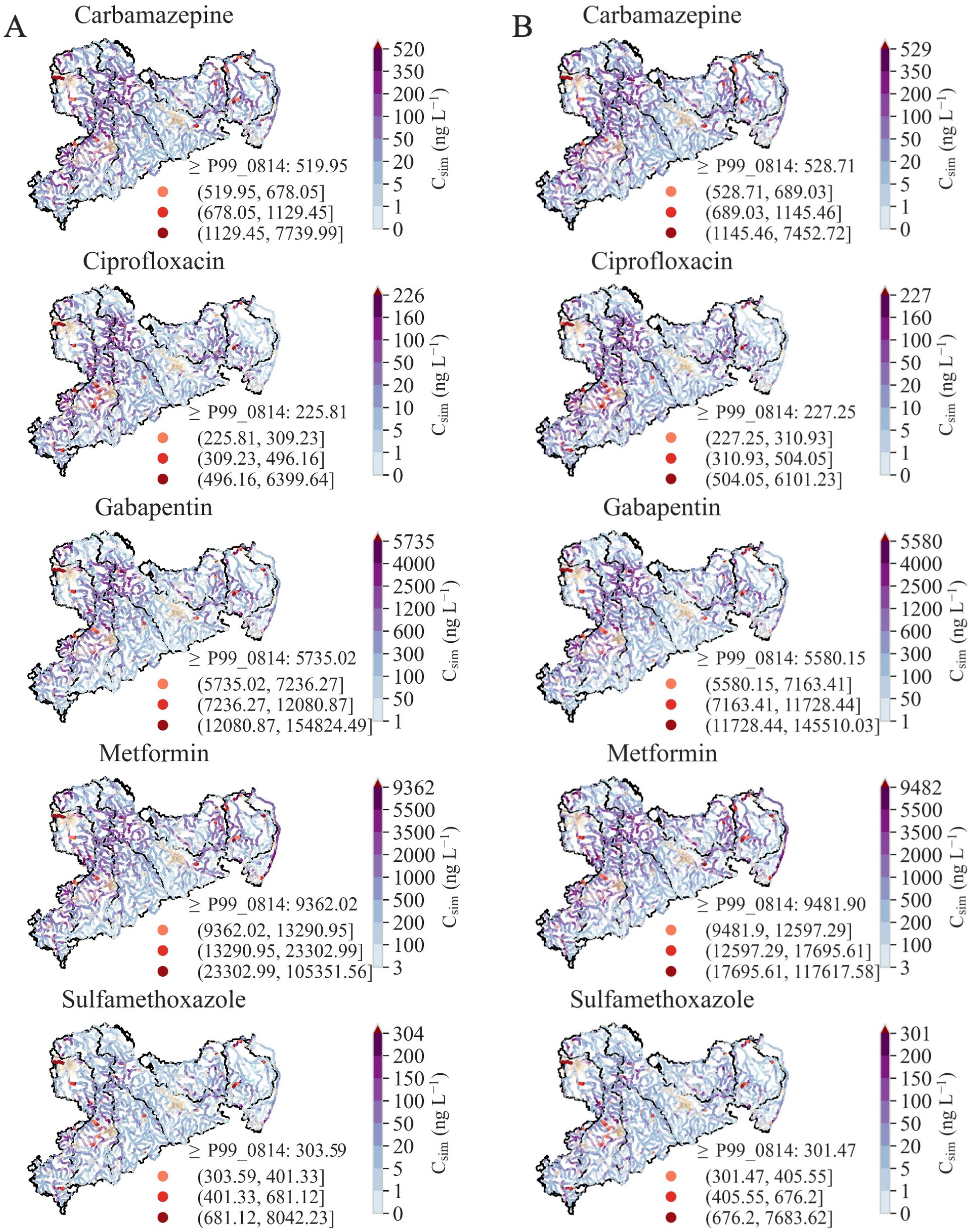

Figure S41: Spatial distribution of simulated concentrations (C_{sim}) in Saxon rivers under Scenario 1 (A) and Scenario 2 (B), reflecting changes in WWTP removal efficiencies. In the panel A, $P99\_0814$ was provided to indicate extreme concentrations, which represents the 99th percentile of the simulations between 2008 and 2014. The orange shaded areas are populated urban regions from the LfULG [1]. Scenario 1 applies removal efficiency adjustments of –10%, –5%, 0%, +5%, and +10% for WWTP classes K1 to K5, respectively. Scenario 2 applies –20%, –10%, 0%, +10%, and +20% for WWTP classes K1 to K5, respectively. Adjustments were applied separately across all pharmaceuticals and seasons. WWTP: centralized wastewater treatment plants.

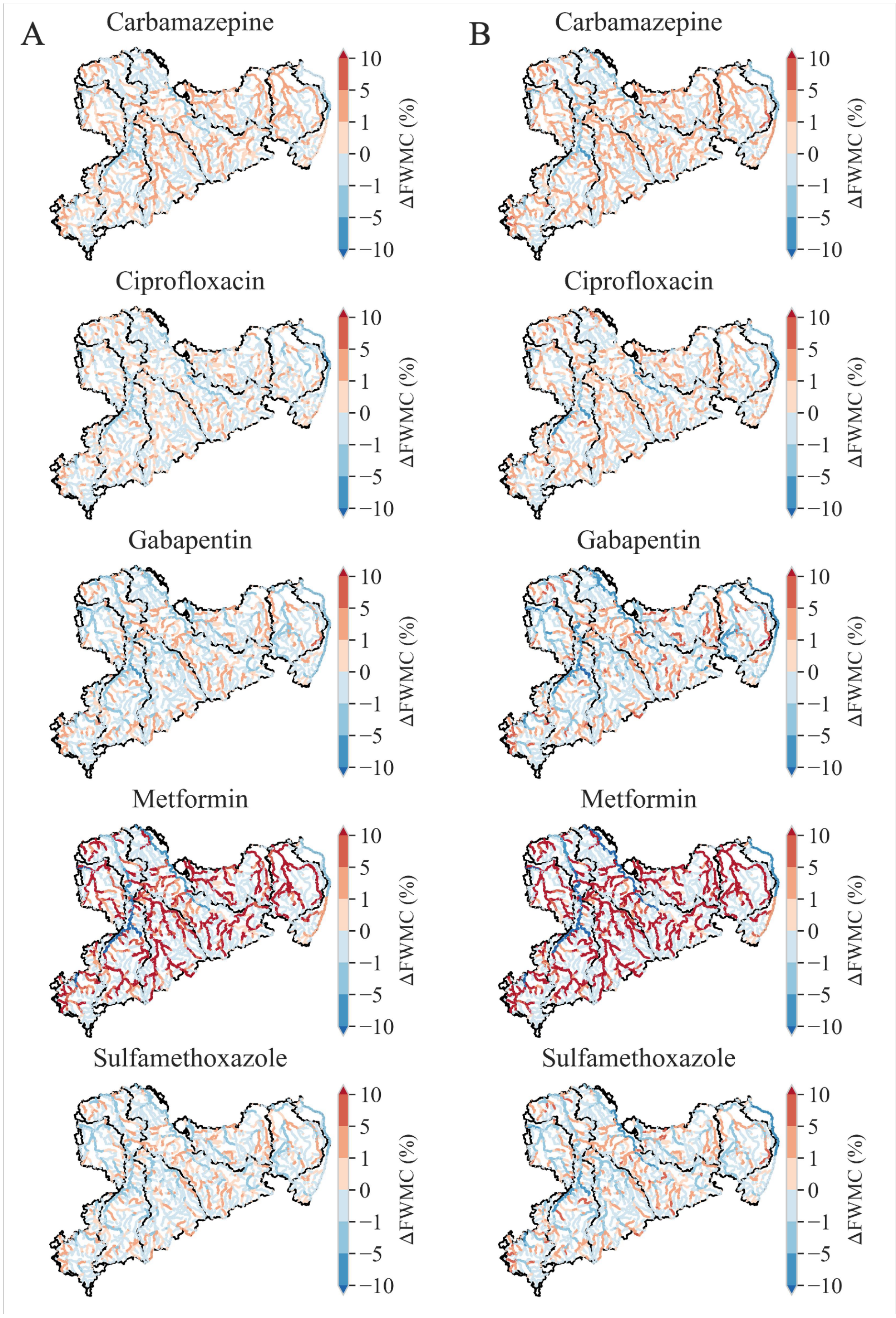

Figure S42: Spatial distribution of relative changes in simulated concentrations ($\Delta FWMC$) across Saxon rivers under Scenario 1 (A) and Scenario 2 (B), reflecting changes in WWTP removal efficiencies. Scenario 1 applies removal efficiency adjustments of –10%, –5%, 0%, +5%, and +10% for WWTP classes K1 to K5, respectively. Scenario 2 applies –20%, –10%, 0%, +10%, and +20% for WWTP classes K1 to K5, respectively. Adjustments were applied separately across all pharmaceuticals and seasons. WWTP: centralized wastewater treatment plants.

References

(1) LfULG LfULG iDA (Interdisciplinary Data and Analyses of Saxon State Agency for Environment, Agriculture and Geology): Water Quality domain, available at:. https://www.umwelt.sachsen.de/umwelt/infosysteme/ida, 2023; [Accessed: 20-June-2023].

(2) Pianosi, F.; Wagener, T. A simple and efficient method for global sensitivity analysis based on cumulative distribution functions. *Environmental Modelling & Software* **2015**, *67*, 1–11.

(3) Pianosi, F.; Wagener, T. Distribution-based sensitivity analysis from a generic input-output sample. *Environmental Modelling & Software* **2018**, *108*, 197–207.

(4) SAFE python the python version of SAFE (Sensitivity Analysis For Everybody) Toolbox: Global Sensitivity Analysis domain, available at:. https://github.com/SAFEtoolbox/SAFE-python, 2024; [Accessed: 3-July-2024].

(5) SMEKUL *30 Jahre kommunale Abwasser-beseitigung im Freistaat Sachsen*; 2020.

(6) Chen, C.; Cowger, W.; Nava, V.; van Emmerik, T. H. M.; Leoni, B.; Guo, Z.-f.; Liu, D.; He, Y.-q.; Xu, Y.-y. Wastewater Discharge Transports Riverine Microplastics over Long Distances. *Environmental Science & Technology* **2024**, *58*, 15147–15158.

(7) Kumar, R.; Hesse, F.; Rao, P. S. C.; Musolff, A.; Jawitz, J. W.; Sarrazin, F.; Samaniego, L.; Fleckenstein, J. H.; Rakovec, O.; Thober, S.; others Strong hydro-climatic controls on vulnerability to subsurface nitrate contamination across Europe. *Nature communications* **2020**, *11*, 6302.

(8) de García, S. O.; Pinto, G. P.; García-Encina, P. A.; Mata, R. I. Ranking of concern, based on environmental indexes, for pharmaceutical and personal care products: an

application to the Spanish case. *Journal of Environmental Management* **2013**, *129*, 384–397.

(9) Verlicchi, P.; Grillini, V.; Lacasa, E.; Archer, E.; Krzeminski, P.; Gomes, A. I.; Vilar, V.; Rodrigo, M. A.; Gäbler, J.; Schäfer, L. Selection of indicator contaminants of emerging concern when reusing reclaimed water for irrigation—A proposed methodology. *Science of The Total Environment* **2023**, *873*, 162359.

(10) PubChem PubChem (US National Library of Medicine, National Center for Biotechnology Information): Octanol-water partition coefficient domain, available at:. https://pubchem.ncbi.nlm.nih.gov, 2025; [Accessed: 18-May-2025].

(11) Yang, S.; Büttner, O.; Kumar, R.; Basso, S.; Borchardt, D. An analytical framework for determining the ecological risks of wastewater discharges in river networks under climate change. *Earth's Future* **2022**, *10*, e2021EF002601.

(12) DEFoJ German Federal Office of Justice: Ordinance on Requirements for the Discharge of Wastewater into Waters, Wastewater Ordinance - AbwV (Verordnung über Anforderungen an das Einleiten von Abwasser in Gewässer, Abwasserverordnung - AbwV): Wastewater treatment plant classification domain, available at:. https://www.gesetze-im-internet.de/abwv/anhang_1.html, 2004; [Accessed: 01-July-2018].

(13) Ahnert, M.; Arndt, M.; Barth, M.; Beil, S.; Börnick, H.; Braeckevelt, M.; Fauler, J.; Fritsche, G.; Günther, E.; Helm, B.; others *Entwicklung eines Stoffflussmodells und Leitfadens zur Emissionsminderung von Mikroschadstoffen im Hinblick auf die Wasserqualität*; 2021; p 33.

(14) DKV DKV (German Hospital Directory): Hospital Locations and Bed Numbers domain, available at:. https://www.german-hospital-directory.com/, 2023; [Accessed: 15-December-2023].

(15) StatsSN Stats-SN (Statistical Agency of the Free State of Saxony): Demographic Data domain, available at:. https://www.statistik.sachsen.de, 2023; [Accessed: 17-June-2023].

(16) GeoSN Geo-SN (Saxon State Agency for Geospatial Information): Terrain Elevation Data domain, available at:. https://www.geodaten.sachsen.de, 2023; [Accessed: 17-June-2023].

(17) USGS USGS GMTED2010 (U.S. Geological Survey, Global Multi-resolution Terrain Elevation Data): Terrain Elevation Data domain, available at:. https://www.usgs.gov/coastal-changes-and-impacts/gmted2010, 2010; [Accessed: 17-June-2023].

(18) Kumar, R.; Samaniego, L.; Attinger, S. Implications of distributed hydrologic model parameterization on water fluxes at multiple scales and locations. *Water Resources Research* **2013**, *49*, 360–379.

(19) Samaniego, L.; Kumar, R.; Attinger, S. Multiscale parameter regionalization of a grid-based hydrologic model at the mesoscale. *Water Resources Research* **2010**, *46*, W05523.

(20) CLMS CLMS (Copernicus Land Monitoring Service): EU-Hydro River Network domain, available at:. https://land.copernicus.eu/en/products/eu-hydro/eu-hydro-river-network-database, 2023; [Accessed: 15-December-2023].

(21) Kramer, L.; Schulze, T.; Klüver, N.; Altenburger, R.; Hackermüller, J.; Krauss, M.; Busch, W. Curated mode-of-action data and effect concentrations for chemicals relevant for the aquatic environment. *Scientific Data* **2024**, *11*, 60.

(22) FDA US FDA (U.S. Food and Drug Administration): Pharmaceutical Pharmacokinetics domain, available at:. https://www.fda.gov/, 2023; [Accessed: 17-June-2023].

(23) Šíma, M.; Bobek, D.; Cihlářová, P.; Ryšánek, P.; Roušarová, J.; Beroušek, J.; Kuchař, M.; Vymazal, T.; Slanař, O. Factors affecting the metabolic conversion of ciprofloxacin and exposure to its main active metabolites in critically ill patients: population pharmacokinetic analysis of desethylene ciprofloxacin. *Pharmaceutics* **2022**, *14*, 1627.

(24) Al-Omar, M. A. *Profiles of drug Substances, excipients and related methodology*; Elsevier, 2005; Vol. 31; pp 209–214.

(25) Chincholkar, M. Gabapentinoids: pharmacokinetics, pharmacodynamics and considerations for clinical practice. *British Journal of Pain* **2020**, *14*, 104–114.

(26) Graham, G. G.; Punt, J.; Arora, M.; Day, R. O.; Doogue, M. P.; Duong, J.; Furlong, T. J.; Greenfield, J. R.; Greenup, L. C.; Kirkpatrick, C. M.; others Clinical pharmacokinetics of metformin. *Clinical Pharmacokinetics* **2011**, *50*, 81–98.

(27) BIO Intelligence Service *Study on the environmental risks of medicinal products, Final Report prepared for Executive Agency for Health and Consumers*; 2013.

(28) Götz, K.; Keil, F. Medikamentenentsorgung in privaten Haushalten: Ein Faktor bei der Gewässerbelastung mit Arzneimittelwirkstoffen? *Umweltwissenschaften und Schadstoff-Forschung* **2007**, *19*, 180–188.

(29) Alnahas, F.; Yeboah, P.; Fliedel, L.; Abdin, A. Y.; Alhareth, K. Expired Medication: Societal, Regulatory and Ethical Aspects of a Wasted Opportunity. *International Journal of Environmental Research and Public Health* **2020**, *17*.

(30) Teran-Velasquez, G.; Helm, B.; Krebs, P. High Spatiotemporal Model-Based Tracking and Environmental Risk-Exposure of Wastewater-Derived Pharmaceuticals across River Networks in Saxony, Germany. *Water* **2023**, *15*, 2001.

(31) Sun, Q.; Lv, M.; Hu, A.; Yang, X.; Yu, C. P. Seasonal variation in the occurrence and removal of pharmaceuticals and personal care products in a wastewater treatment plant in Xiamen, China. *Journal of Hazardous Materials* **2014**, *277*, 69–75.

(32) Malato, S.; Giménez, J.; Oller, I.; Agüera, A.; Sánchez Pérez, J. A. *Removal and Degradation of Pharmaceutically Active Compounds in Wastewater Treatment*; Springer, 2020; pp 299–326.

(33) Wang, J.; Wang, S. Removal of pharmaceuticals and personal care products (PPCPs) from wastewater: A review. *Journal of Environmental Management* **2016**, *182*, 620–640.

(34) Patel, M.; Kumar, R.; Kishor, K.; Mlsna, T.; Pittman, C. U.; Mohan, D. Pharmaceuticals of emerging concern in aquatic systems: Chemistry, occurrence, effects, and removal methods. *Chemical Reviews* **2019**, *119*, 3510–3673.

(35) Nas, B.; Dolu, T.; Koyuncu, S. Behavior and Removal of Ciprofloxacin and Sulfamethoxazole Antibiotics in Three Different Types of Full-Scale Wastewater Treatment Plants: A Comparative Study. *Water, Air, & Soil Pollution* **2021**, *232*, 127.

(36) Wiest, L.; Gosset, A.; Fildier, A.; Libert, C.; Hervé, M.; Sibeud, E.; Giroud, B.; Vulliet, E.; Bastide, T.; Polomé, P.; others Occurrence and removal of emerging pollutants in urban sewage treatment plants using LC-QToF-MS suspect screening and quantification. *Science of The Total Environment* **2021**, *774*, 145779.

(37) Burns, E. E.; Carter, L. J.; Kolpin, D. W.; Thomas-Oates, J.; Boxall, A. B. Temporal and spatial variation in pharmaceutical concentrations in an urban river system. *Water Research* **2018**, *137*, 72–85.

(38) Björlenius, B.; Ripszám, M.; Haglund, P.; Lindberg, R. H.; Tysklind, M.; Fick, J. Pharmaceutical residues are widespread in Baltic Sea coastal and offshore waters–Screening

for pharmaceuticals and modelling of environmental concentrations of carbamazepine. *Science of the Total Environment* **2018**, *633*, 1496–1509.

(39) Scheurer, M.; Sacher, F.; Brauch, H.-J. J. Occurrence of the antidiabetic drug metformin in sewage and surface waters in Germany. *Journal of Environmental Monitoring* **2009**, *11*, 1608–1613.

(40) Henning, N.; Kunkel, U.; Wick, A.; Ternes, T. A. Biotransformation of gabapentin in surface water matrices under different redox conditions and the occurrence of one major TP in the aquatic environment. *Water Research* **2018**, *137*, 290–300.

(41) Li, Z.; McLachlan, M. S. Biodegradation of chemicals in unspiked surface waters downstream of wastewater treatment plants. *Environmental Science & Technology* **2019**, *53*, 1884–1892.

(42) Baena-Nogueras, R. M.; González-Mazo, E.; Lara-Martín, P. A. Degradation kinetics of pharmaceuticals and personal care products in surface waters: photolysis vs biodegradation. *Science of The Total Environment* **2017**, *590*, 643–654.

(43) Hanamoto, S.; Nakada, N.; Yamashita, N.; Tanaka, H. Modeling the Photochemical Attenuation of Down-the-Drain Chemicals during River Transport by Stochastic Methods and Field Measurements of Pharmaceuticals and Personal Care Products. *Environmental Science & Technology* **2013**, *47*, 13571–13577.

(44) Ehalt Macedo, H.; Lehner, B.; Nicell, J.; Grill, G. HydroFATE (v1): a high-resolution contaminant fate model for the global river system. *Geoscientific Model Development* **2024**, *17*, 2877–2899.

(45) Grill, G.; Khan, U.; Lehner, B.; Nicell, J.; Ariwi, J. Risk assessment of down-the-drain chemicals at large spatial scales: Model development and application to contaminants

originating from urban areas in the Saint Lawrence River Basin. *Science of the Total Environment* **2016**, *541*, 825–838.

(46) Oldenkamp, R.; Hoeks, S.; Čengić, M.; Barbarossa, V.; Burns, E. E.; Boxall, A. B.; Ragas, A. M. A high-resolution spatial model to predict exposure to pharmaceuticals in European surface waters: EPiE. *Environmental Science & Technology* **2018**, *52*, 12494–12503.

(47) Tong, Y.; Qi, M.; Sun, P.; Qin, W.; Zhu, Y.; Wang, X.; Xu, Y.; Zhang, W.; Yang, J. Estimation of unintended treated wastewater contributions to streams in the Yangtze River Basin and the potential human health and ecological risk analysis. *Environmental Science & Technology* **2022**, *56*, 5590–5601.

(48) Kehrein, N.; Berlekamp, J.; Klasmeier, J. Modeling the fate of down-the-drain chemicals in whole watersheds: New version of the GREAT-ER software. *Environmental Modelling & Software* **2015**, *64*, 1–8.

(49) Lämmchen, V.; Niebaum, G.; Berlekamp, J.; Klasmeier, J. Geo-referenced simulation of pharmaceuticals in whole watersheds: application of GREAT-ER 4.1 in Germany. *Environmental Science and Pollution Research* **2021**, *28*, 21926–21935.

(50) Lindim, C.; Van Gils, J.; Cousins, I.; Kühne, R.; Georgieva, D.; Kutsarova, S.; Mekenyan, O. Model-predicted occurrence of multiple pharmaceuticals in Swedish surface waters and their flushing to the Baltic Sea. *Environmental Pollution* **2017**, *223*, 595–604.
